# Sea Cucumber Intestinal Peptide Induces the Apoptosis of MCF-7 Cells by Inhibiting PI3K/AKT Pathway

**DOI:** 10.3389/fnut.2021.763692

**Published:** 2021-12-14

**Authors:** Wei Wei, Xiao-Man Fan, Shao-Hui Jia, Xi-Ping Zhang, Zhao Zhang, Xue-Jun Zhang, Jiu-Xun Zhang, Ye-Wang Zhang

**Affiliations:** ^1^School of Pharmacy, Jiangsu University, Zhenjiang, China; ^2^Shandong Tianjiu Industry Group, Heze, China; ^3^Hubei Key Laboratory of Sport Training and Monitoring, Tianjiu Research and Development Center for Exercise Nutrition and Foods, College of Health Science, Wuhan Sports University, Wuhan, China

**Keywords:** sea cucumber intestinal peptide, apoptosis, MCF-7, PI3K/AKT, inhibition

## Abstract

Sea cucumbers are one of many marine echinoderm animals that contain valuable nutrients and medicinal compounds. The bioactive substances in sea cucumbers make them have promising biological and pharmacological properties, including antioxidant, anti-bacterial, and anti-tumor effects. In this study, sea cucumber intestinal peptide (SCIP) is a small molecular oligopeptide (<1,000 Da) extracted from sea cucumber intestines hydrolyzed by alkaline protease. The analysis of amino acid composition showed that hydrophobic amino acids and branched-chain amino acids were rich in SCIP. Nowadays, although increasing studies have revealed the biological functions of the sea cucumber active substances, there are few studies on the function of SCIP. Furthermore, due to the anti-cancer activity being an essential characteristic of sea cucumber active substances, we also investigated the anti-cancer potential and the underlying mechanism of SCIP *in vivo* and *in vitro*. The results indicate that SCIP inhibits the growth of MCF-7 tumor cells in zebrafish and increases the apoptosis of human breast cancer MCF-7 cells. Further mechanism studies confirm that SCIP promotes the expression of apoptosis-related proteins and thus promotes the breast cancer cells (MCF-7) apoptosis via inhibition of PI3K/AKT signal transduction pathway.

## Introduction

Sea cucumber, also called trepang, beach-demer, gamat, balate, or haishen, belonging to the *Phylum echinodermata*, has been utilized for centuries as a nutritious and functional food with various bioactivities in China and other Asian countries (1, 2). It has various active substances and plays an important biological function, including antioxidant, antibacterial, anti-tumor, and so on (3, 4). A synthetic derivative of antimicrobial peptide Holothuroidea 2 from Mediterranean Sea cucumber (*Holothuria tubulosa*) has been considered to have an inhibitory effect on *Listeria monocytogenes* (5). Very recently, it was found that the sea cucumber tegument extract inhibits 99% of the virus during the absorption and viral inactivation phase (6). Anti-cancer effect is the most attractive function of the sea cucumber active substances. Frondoside A is a natural glycoside extracted from the sea cucumber, which has been used as a traditional remedy. This compound was found to inhibit tumor growth and reduce tumor volume by 87% in an athymic mouse model using MDA-MB-231 breast cancer cells (7). Similarly, sea cucumber extract TBL-12 has been reported to inhibit the proliferation, migration, and invasion of human prostate cancer cells through the p38 mitogen-activated protein kinase and intrinsic caspase apoptosis pathway (8). Triterpene glycosides are characteristic secondary metabolites of sea cucumbers (*Holothuroidea, Echinodermata*), and intraperitoneal administration of solutions of these glycosides in rodents significantly reduces both tumor burden and metastasis (9). The previous research regarding the anti-cancer mechanism of the sea cucumber substances mainly focused on cytotoxic activity, induction of apoptosis, cell cycle arrest, inhibition of tumor growth, antimetastatic and anti-angiogenic properties, and inhibition of drug resistance (10).

Previous reports of sea cucumber mainly focused on non-protein bioactive compounds and their various bioactivities. Recently, bioactive peptides are considered one of the important potential therapeutic substrates in sea cucumber (4). The bioactivities of enzymatic hydrolysates from the viscera of Atlantic Sea cucumber were reported to have antiviral and antioxidant activities (5). Similarly, hydrolysates produced from the whole animal by protamex have been found to have anti-aging properties (11). However, up to now, the anti-cancer function and underlying mechanism of sea cucumber intestinal peptide (SCIP) are still unclear. In this study, we firstly obtained SCIP from the intestine tissue of sea cucumber by alkaline enzymatic hydrolysis and then investigated the effects and mechanisms of SCIP on tumor cells *in vivo* and *in vitro*.

## Results

### The Molecular Weight Distribution of SCIP

**Figure 1:**
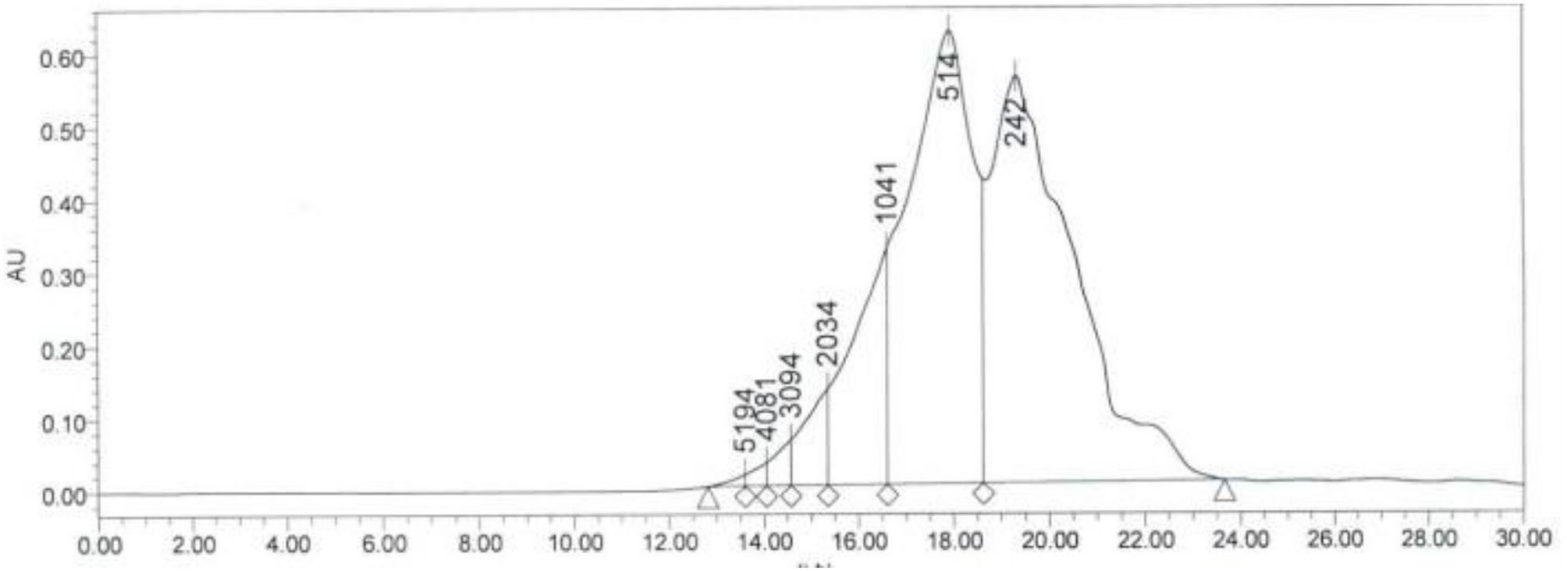
The molecular weight distribution of SCIP investigated by high-performance liquid chromatography (HPLC).

In order to further purify and determine the amino acid distribution of SCIP, the prepared peptides were further separated by high-performance liquid chromatography (HPLC). As shown in Figure 1, the SCIP consists of a series of oligopeptides. In addition, it was found that there was 98.41% of SCIP with a relative molecular weight <2,000 Da, and there was around 95.36% of SCIP distributed in the molecular weight range <1,041 Da.

### The Amino Acid Composition of SCIP

According to the results of amino acid analysis (Table 1), the content of amino acids in SCIP is up to 80.5%. The contents of Ser and Gly were 8.66 and 8.07%, respectively, which were the most abundant amino acids in SCIP. Moreover, the branched-chain amino acids (Val, Ile, and Leu) were also relatively rich compared with the others. In general, the contents of hydrophobic amino acids including Ala, Val, Leu, Gly, Phe, and Met are rich in SCIP, and the ratio is about 22.65%.

**Table 1:** Amino acid composition of the sea cucumber intestinal peptide (SCIP) sample (g/100 g peptide).

**Name**	**Height**	**Area**	**ESTD** **(×100 Conc/nmol)**	**%**
Asp	164,832	2,739,532	5.11	6.85
Thr	89,495	1,471,783	2.92	3.50
Glu	94,317	1,599,105	3.19	4.75
Ser	207,445	4,517,389	8.20	8.66
Gly	197,531	4,616,951	10.78	8.07
Ala	64,352	1,832,353	4.93	4.39
Cys	9,111	137,998	0.22	0.58
Val	96,425	1,957,465	3.79	4.45
Met	22,770	602,353	1.02	1.43
Ile	37,983	1,207,000	2.31	2.95
Leu	53,908	2,015,612	4.57	5.98
Tyr	9,744	302,199	0.68	1.26
Phe	34,042	805,215	1.42	2.33
Lys	143,205	2,705,591	3.88	5.69
NH_3_	221,464	6,124,942	0.00	0.00
His	21,472	508,305	0.79	1.27
Arg	49,213	1,730,797	2.91	5.02

### SCIP Inhibits the Growth of Tumor Cells in Zebrafish

**Figure 2:**
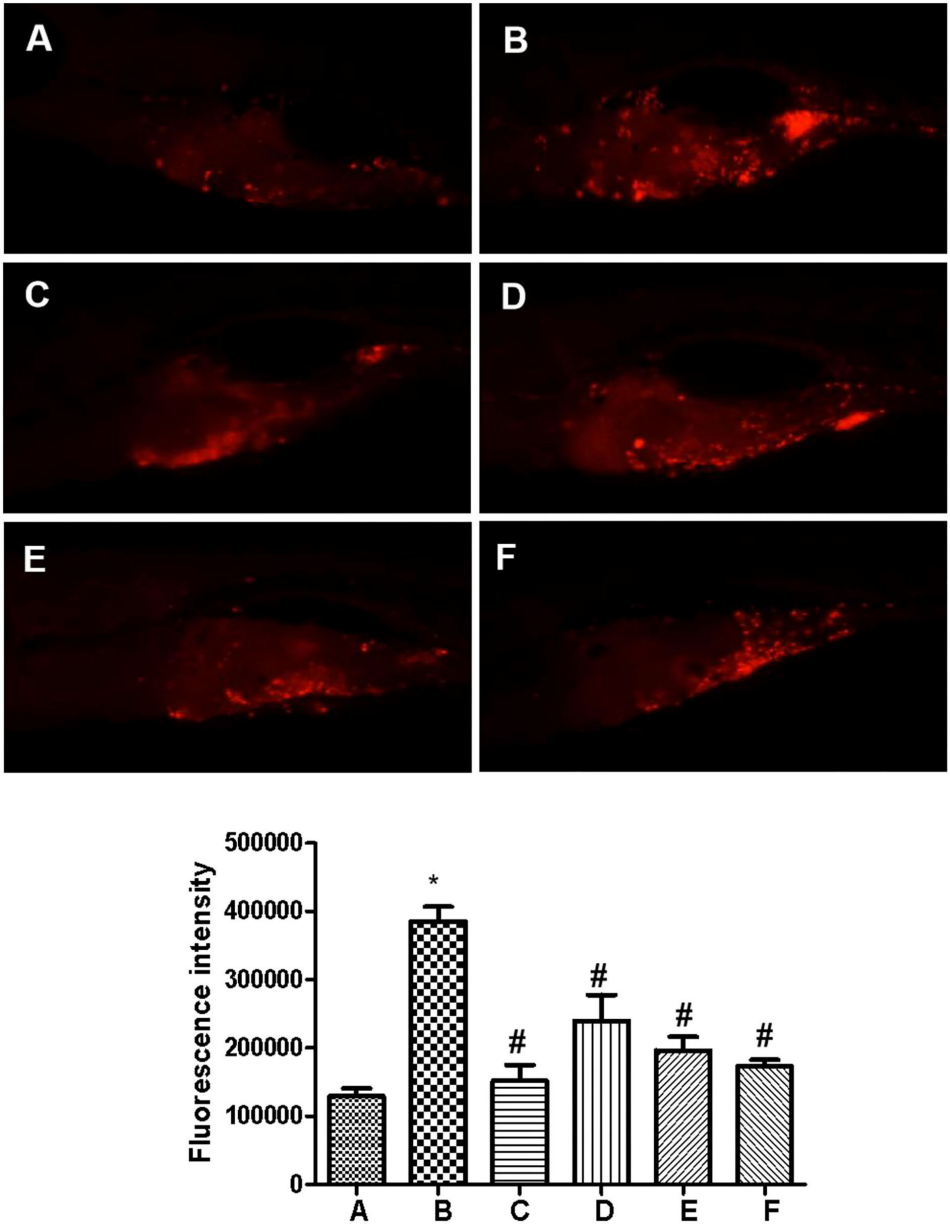
Sea cucumber intestinal peptide inhibits the growth of tumor cells in zebrafish. The wild-type AB zebrafish do not produce red fluorescence. CM-Dil labeled cells injected into the yolk sac of zebrafish can produce red fluorescence at a certain wavelength. The total fluorescence intensity is positively correlated with the number of cancer cells. **(A)** Control group; **(B)** Model group; **(C)** Capecitabine group, administration of 20 μg/ml capecitabine by water-soluble; **(D)** Low concentration SCIP group, administration of 27.8 μg/ml SCIP by water-soluble; **(E)** Middle concentration SCIP group, administration of 83.3 μg/ml SCIP by water-soluble; **(F)** High concentration SCIP group, administration of 250 μg/ml SCIP by water-soluble. “*” means that the experimental group was significant. On the contrary, the “#” means that the experimental group is not significant.

As shown in Figure 2, the fluorescence intensity of zebrafish in the model group was significantly stronger than that in the control group while capecitabine administration significantly reduced the fluorescence intensity in zebrafish, which means inhibiting the growth of zebrafish human breast cancer cell (MCF-7) xenografts. Furthermore, compared to the model group, administration of SCIP obviously decreased the fluorescence intensity and inhibited the growth of human breast cancer cell xenografts in zebrafish in a dose-dependent manner.

### SCIP Inhibits the Proliferation of MCF-7 *in vivo*

**Figure 3:**
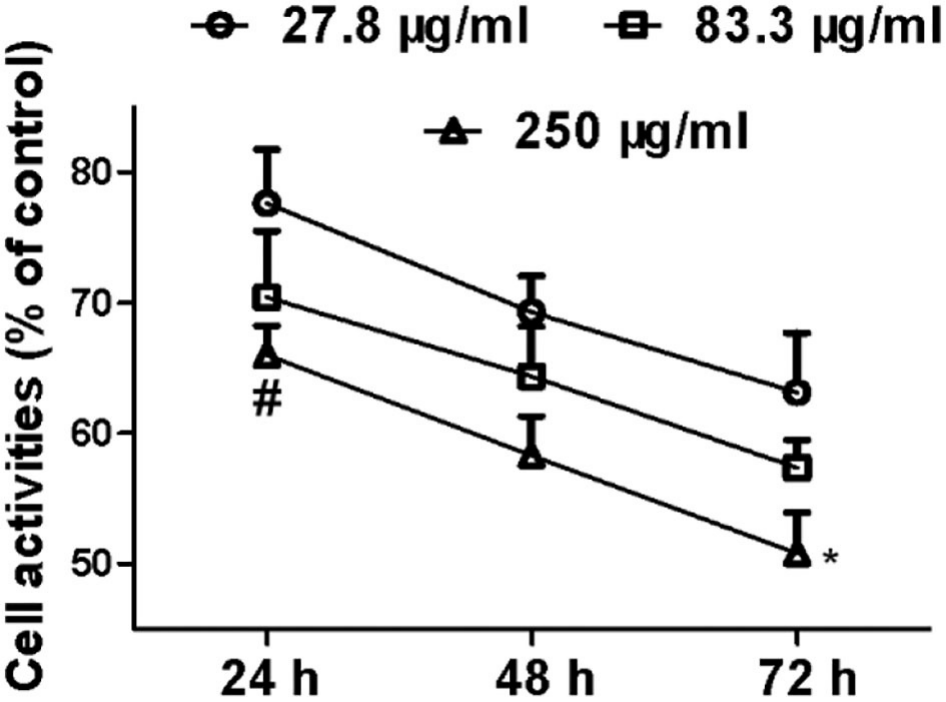
SCIP inhibits the proliferation of MCF-7 cells. Cells were synchronized and incubated with SCIP at various concentrations (27.8, 83.3, and 250 μg/ml) for 24, 48, and 72 h, respectively. Then, the cell samples were harvested and subjected to investigate the proliferation of MCF-7 cells by MTT analysis. Each value was expressed as mean ± SD for three independent experiments by GraphPad Prism Software Version 5.0. ^*^p < 0.05 was considered a significant difference when compared with 24 h, and ^#^*p* < 0.05 was considered a significant difference compared to the low dose group.

After the incubation of MCF-7 with SCIP for 24 h, 48 h, and 72 h (Figure 3), the activities of MCF-7 cells showed a time-dependent downward in all the SCIP groups (27.8, 83.3, and 250 μg/ml). After incubation of 72 h with SCIP, the activity of cells in the high dose group (250 μg/ml) was significantly lower than that in the 24 h of SCIP stimulation, while it was not found in the low dose group (27.8 μg/ml) and the medium-dose group (83.3 μg/ml). After incubation for 24 h, compared with the low dose SCIP group, the cell activities of the high dose SCIP group showed a significant decrease (*p* < 0.05).

### SCIP Promotes the Apoptosis of MCF-7 Cells

**Figure 4:**
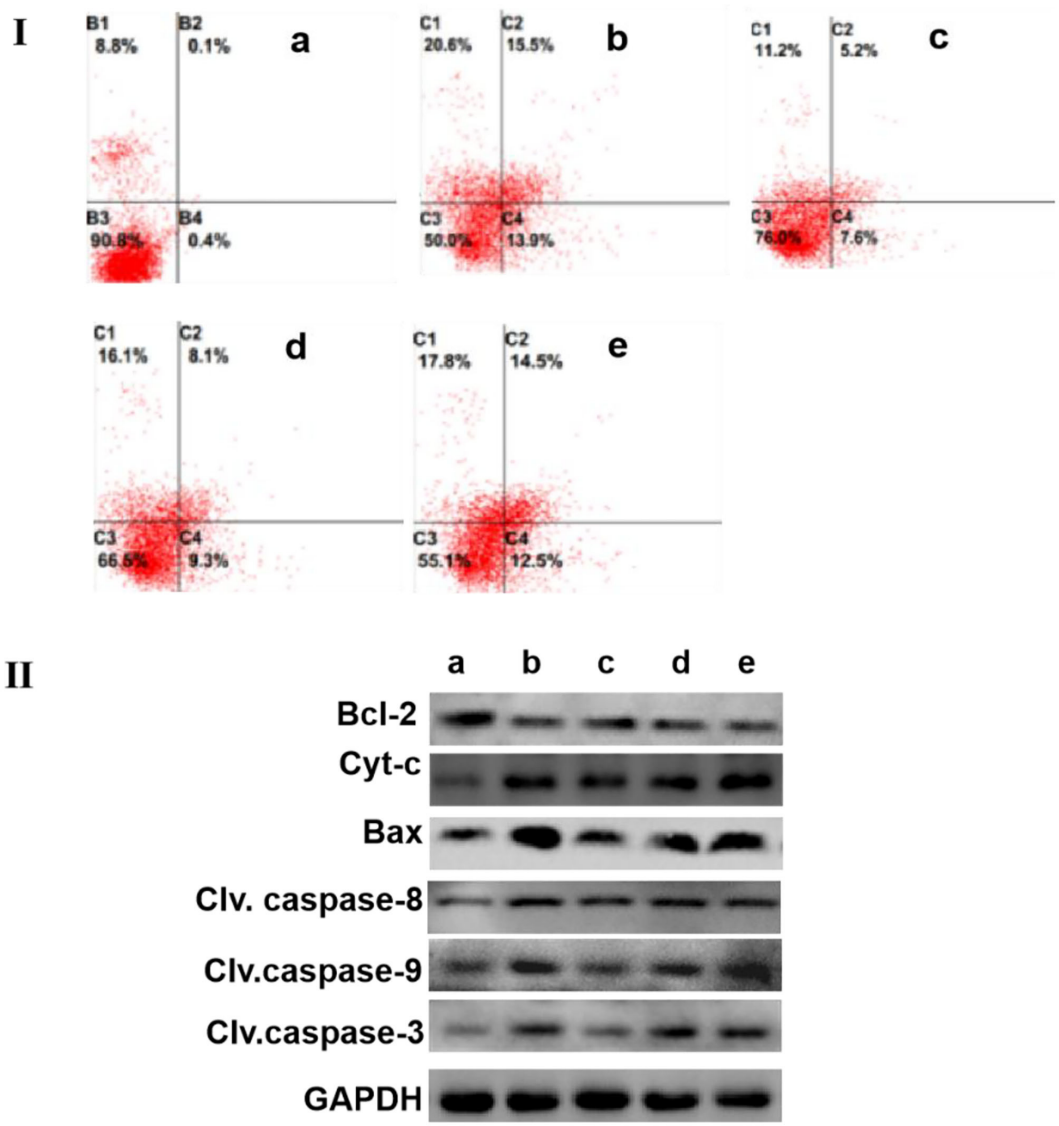
SCIP improves the apoptosis of MCF-7. **(I)** The apoptosis level of MCF-7 cells was determined by flow cytometry analysis. MCF-7 Cells were cultured and incubated with 20 μg/ml of capecitabine and various concentrations of SCIP (27.8, 83.3, and 250 μg/ml) for 12 h, then, MCF-7 cells were stained with annexin V and PI, and flow cytometry was carried out to detect its apoptosis rate. **(II)** The expression of apoptosis-related proteins was investigated by western blots. MCF-7 cells were cultured and incubated with 20 μg/ml of capecitabine and various concentrations of SCIP (27.8, 83.3, and 250 μg/ml) for 12 h; After the treatment, the cells were collected to extract the total proteins and performed the western blot analysis to measure the expression of apoptosis proteins. a: Control group, MCF-7 cells were stimulated by PBS; b: Capecitabine group, MCF-7 cells were incubated by 20 μg/ml of capecitabine for 12 h; c: Low dose SCIP group, MCF-7 cells were incubated by 27.8 μg/ml of SCIP for 12 h; d: Moderate dose SCIP group, MCF-7 cells were incubated by 83.3 μg/ml of SCIP for 12 h; f: High dose SCIP group, MCF-7 cells were incubated by 250 μg/ml of SCIP for 12 h.

The results of flow cytometry showed that the apoptosis of H_2_O_2_-treated MCF-7 cells largely increased when compared with those in the control group. Similarly, the incubation of capecitabine for 12 h significantly enhanced the apoptosis of MCF-7 cells. Meanwhile, when the MCF-7 cells were co-cultured with SCIP overnight, and the apoptosis rate of cells gradually increased in pace with the elevation of SCIP concentration (Figure 4-I).

To further elucidate the potential mechanism of MCF-7 cells apoptosis induced by SCIP, western blots were performed to detect the expression levels of apoptotic-related signaling pathway-related proteins. The results revealed that SCIP incubation for 12 h elevated the expression of pro-apoptosis proteins, including bax, cleaved caspase-9, cleaved caspase-3, and Cyt-c, while it did not up-regulate cleaved caspase-8 expression. On the contrary, it decreased the anti-apoptosis protein expression of bcl-2 in a dose-dependent manner (Figure 4-II).

### SCIP Induced MCF-7 Cells Apoptosis via Inactivation of PI-3K/AKT Signaling Pathway

**Figure 5:**
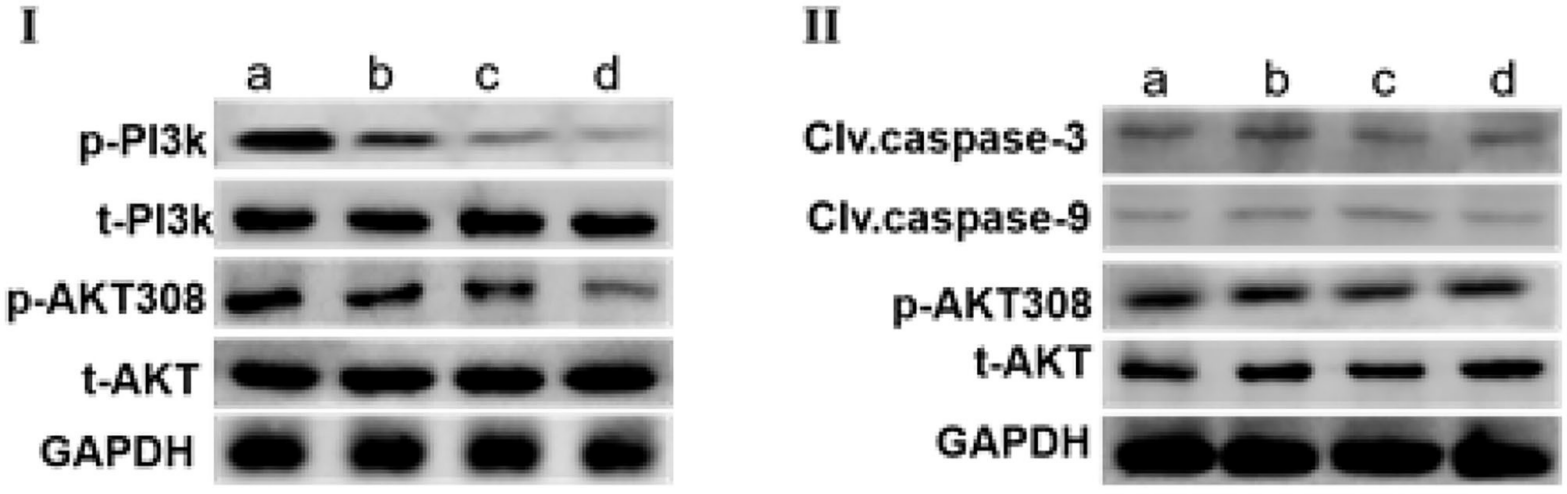
SCIP promotes the apoptosis of MCF-7 by activation of PI3K/AKT signaling pathway. **(I)** MCF-7 cells were treated with various concentrations of SCIP (0, 27.8, 83.3, and 250 μg/ml) for 24 h, then collected the cells to extract the total proteins and western blots were used to investigate the expression of total AKT (t-AKT), p-AKT, t-PI3k, and p-PI3k, respectively (*n* = 3). **(II)** MCF-7 cells were pretreated with wortmannin for 1 h, then incubated with SCIP (0, 27.8, 83.3, and 250 μg/ml) for 24 h. After treatment, the MCF-7 cells were harvested to detect the expression of t-AKT, p-AKT, Clv.caspase-9, and Clv.caspase-3 (*n* = 3).

As shown in Figure 5-I, incubation MCF-7 cells with different concentrations of SCIP for 24 h strongly reduced the expression of p-PI3k and p-AKT308 in MCF-7 in a dose-dependent manner when compared with that in the control group. Whereas, after pretreatment of MCF-7 with PI3K inhibitor wortmannin for 1 h, stimulation of MCF-7 with various concentrations of SCIP for 24 h did not inhibit the phosphorylation of AKT, and decrease the expression of clv.casepase-9 and clv.casepase-3 (Figure 5-II).

## Discussion

Sea cucumbers are one of the marine animals which are valuable sources of nutritious foods for humans, especially in Asian countries. They have been well-recognized as a tonic and traditional remedy in Chinese and Malaysian residents for their effectiveness against hypertension, asthma, rheumatism, cuts and burns, impotence, and constipation (12).

The previous studies have shown that numerous sea cucumber substances have a wide range of biological activities, including antimicrobial, anti-inflammatory, and anti-cancer (13, 14). Accumulating evidence have suggested that anti-cancer activity attributes to the most attractive biological substance extracted from sea cucumber, and the underlying mechanism includes induction of cells apoptosis, promotion of cell cycle arrest, and inhibition of tumor growth (8–10).

Previous studies have confirmed that many amino acids are rich in sea cucumber, especially the essential amino acids, and it is of great benefit to human health (15). Antioxidative peptides from fish have also been reported with a high amount of specific amino acids composition such as tyrosine, tryptophan, methionine, cysteine, and histidine (16). In the present work, the results of amino acid composition analysis demonstrated that hydrophobic amino acids and branched-chain amino acids were abundant in SCIP. However, although we have confirmed that SCIP is a promising anti-cancer bioactive peptide, it is still unclear whether this effect is triggered by its unique amino acid composition.

Owing to lots of studies that have manifested that the active components of sea cucumber can inhibit the growth of cancer cells in a variety of ways (1, 10, 17), we investigated the anti-cancer effect of SCIP. Initially, we estimated the effect of SCIP on the growth of MCF-7 xenografts in zebrafish and found that SCIP obviously suppressed the growth of MCF-7 xenografts in a dose-dependent manner. To further confirm the anti-cancer effect of SCIP, we then detected its ability to induce apoptosis of breast cancer cell MCF-7 *in vitro*. The results demonstrated that SCIP significantly accelerated the apoptosis of breast cancer cells in a dose-dependent manner, especially early apoptosis.

There are two classic apoptosis pathways in mammalian cells, namely, the death receptor-mediated extrinsic pathway and the mitochondria-mediated intrinsic pathway, which are controlled by caspase-8 and caspase-9, respectively. Activated caspase-8 and caspase-9 then activate downstream caspase-3 and/or caspase-7, which in turn cleave poly (ADP)-ribose polymerase (PARP) and other downstream target proteins, finally resulting in cell apoptosis (16–18). As shown in the results of western blots, compared to the control, SCIP treatment largely enhanced the expression of cleaved caspase-9 and the downstream cleaved caspase-3, while SCIP had no effect on cleaved caspase-8 in a dose-dependent manner. These results indicated that SCIP stimulated MCF-7 cells apoptosis *in vitro* mainly by activation of endogenous apoptosis pathway rather than exogenous apoptosis pathway. Subsequently, we observed that SCIP incubation for 12 h markedly up-regulated the expression of bcl-2, and obviously reduced bax expression compared with that of the control group. Bax is the protein acting contrary role to bcl-2 in the process of apoptosis. When Bax forms a homologous dimer, the induction of apoptosis increases. When Bax and bcl-2 form a hybrid heterodimer, the anti-apoptotic function of bcl-2 is activated, and then apoptosis is inhibited (19, 20). Cytochrome c (Cyt-c) is a cellular life and death decision molecule that regulates cellular energy supply and apoptosis through tissue-specific post-translational modifications. Under conditions of cellular stress, Cyt-c release from the mitochondria is a committing step for apoptosis, leading to apoptosome formation, caspase activation, and cell death (21). Our results also revealed that SCIP treatment elevated the Cyt-c expression in cultured MCF-7 cells. It is generally considered that Cyt-c was a key factor of the intrinsic apoptosis pathway. Some stimulating factors, including oxidative stress-induced mitochondrial damage, resulted in Cyt-c release to the cytoplasm. Once released into the cytosol, the elevated Cyt-c and Apaf1 further triggered intrinsic apoptosis via executive molecular caspase-9 and caspase-3 (22). According to the above results, we believe that SCIP-induced apoptosis in breast cancer cell MCF-7 mainly depends on an endogenous apoptotic pathway.

Phosphatidylinositol 3-kinases (PI3Ks) are the crucial coordinators of intracellular signaling in response to the extracellular stimulators. Hyperactivation of PI3K signaling cascades is one of the most ordinary events in human cancers (23). PI3K/AKT pathways are the most frequently altered in human cancers. Aberrant activation of this pathway, as a result of these somatic alterations, is associated with cellular transformation, tumorigenesis, cancer progression, and drug resistance (24). Activation of the PI3K/AKT signaling pathway has been implicated in tumorigenesis, breast cancer progression, and in resistance to standard therapies (25–27). Up to now, PI3K/AKT has been widely used as a new target for the development of breast cancer drugs (28–31). In this study, SCIP incubation decreased the phosphorylation of PI3K and its downstream target AKT. However, the pretreatment of MCF-7 cells with PI3K inhibitor eliminated the decrease of PI3K and Akt phosphorylation, and the enhancement of clv.caspase-9 and clv.caspase-3, which is induced by SCIP. PI3K/Akt signaling pathway is considered as an important regulator of apoptosis activation by regulating the expression of the apoptosis-related proteins such as Bcl-2, Bax, clv.caspase-9, clv. Caspase-3, and so on (32–34). Thus, in the present study, SCIP triggered the inhibition of PI3K/AKT signaling pathway is the key factor for the apoptosis of MCF-7 cells.

In summary, our research indicated that the rich branched-chain amino acids and hydrophobic amino acids in SCIP could promote the apoptosis of MCF-7 cells by inhibiting PI3K/Akt signaling pathway, subsequently inducing the significant activation of the endogenous apoptotic pathway by increasing or up-regulating the expression of Bcl-2, Bax, clv.caspase-9, clv. Caspase-3, and Cyt-c. Although its detailed mechanism remains unclear, this is the first report on the anti-cancer effect of sea cucumber intestinal peptides.

## Materials and Methods

### Purification of Sea Cucumber Intestinal Peptide

Intestinal sea cucumbers from Dalian of China were crushed and freeze-dried. The lyophilizing powder was suspended in distilled with the ratio of 1:2 and kept mixture at 55°C for 1 h. Subsequently, adjusted pH of the mixture to 8.5 with NaOH solution and raised the temperature to 60°C. Then, 3% alkaline protease (Aladdin, Shanghai, China) was added for hydrolysis for 4 h at 50°C and pH 7. Afterward, the temperature was raised to 100°C and maintained for 5 min to inactivate the enzyme. When the solution temperature dropped to 55~60°C, activated carbon was added into and maintained the temperature for 1 h. Then, the solution was filtrated by 30,000 Da ceramic membrane and collected. After, the filtrate was purified again with 1 kDa ultrafiltration membrane and collected. Finally, the fraction with a molecular weight of <5 kDa was collected and spray dried to obtain SCIP.

### Determination of Molecular Weight Distribution and Amino Acid Composition of SCIP

The above prepared SCIP was redissolved in 5 M HCl and then hydrolyzed in an anaerobic environment at 120°C for 22 h, and then the samples were neutralized with 10 M NaOH. After the treatment, the liquid compound flowed through the double filter paper was collected. The obtained samples were analyzed with HPLC (HPLC, Agilent 1100, Tokyo, Japan) to obtain the composition of amino acids. The purification conditions are listed as follows: the flow ratio was 0.5 ml/min with the mobile phase of acetonitrile/water/trifluoroacetic acid (45:55:0.1, v/v/v). For evaluation of the molecular weight of SCIP, Cytochrome C (Mw 12,384 Da), aprotinin (Mw 6,512 Da), bacitracin (Mw 1,422 Da), and glycine-glycine-tyrosine-arginine (Mw 451 Da) and glycine glycine-glycine (Mw 189 Da) were used as the molecular weight markers.

The purified products of RP-HPLC were collected, and the amino acid composition of SCIP was determined using Amino Acid Analyzer (LA8900, Hitachi, Japan).

### SCIP Inhibits the Growth of Human Breast Cancer (MCF-7) Xenografts in Zebrafish

To establish the zebrafish cancer model, CM-Dil labeled human breast cancer cells of MCF-7 purchased from China Center for Type Culture Collection (CCTCC) were transplanted into the 2 DPF wild type AB zebrafish yolk sac by micro-injection. About 300 cells were transplanted into each tail, and a zebrafish human breast cancer (MCF-7) transplantation model was established. After the model is established successfully, zebrafish with good consistency of transplanted tumor cells were selected under the microscope and randomly assigned to a 6-well plate with 30 zebrafish each well. Then, 20 μg/ml capecitabine (positive control) and different concentrations of SCIP (27.8, 83.3, and 250 μg/ml) were administrated to the zebrafish with MCF-7 cells using the water-soluble method. The zebrafish (zebrafish) in each experimental group were cultured at 35°C for 2 days. Then, the fluorescence images of MCF-7 xenografts were obtained by fluorescence microscope, and image analysis was performed by Nikon NIS-Elements D 3.10 (Nikon Corporation, Japan) advanced image software to calculate the fluorescence intensity of cancer cells. Finally, the inhibitory effect of SCIP on zebrafish breast cancer (MCF-7) cell xenograft was evaluated by fluorescence intensity using an electric focusing continuous zoom fluorescence microscope (AZ100, Nikon, Japan).

### MTT Analysis

To determine the effect of SCIP on the activity of breast cancer cells, MCF-7 cell lines growing in the logarithmic phase were put into sterile 96-well plates with the concentration of 2 × 10^5^ cells/ml and cultured for 24 h at 37°iC in 5% CO_2_ incubator. Then, 100 ul of SCIP with different concentrations (27.8, 83.3, and 250 μg/ml) was added into 96-well plates. After incubation, 20 μl of MTT solution (5 mg/mL, 0.5% MTT) was added to each well at various times (24, 48, and 72 h), and was allowed to culture for 4 h at 37°C. After the centrifugation, the supernatant was discarded, and 100 μl dimethylsulfoxide (DMSO) was added into each well. The plates were then put on the shaker and vibrated at low speed for 10 min to make the crystal dissolve completely. Finally, the absorbance of each well was measured at 490 nm by ELISA.

### Flow Cytometry Analysis

The cell apoptosis was detected by flow cytometry according to the manufacturer's protocol. Briefly, MCF-7 cells were seeded into 6-well plates with a concentration of 1 × 10^6^ cells/ml and cultured for 24 h at 37°C in 5% CO_2_. Then, the medium was changed with fresh medium, and different concentrations of SCIP (27.8, 83.3, and 250 μg/ml) were added into the plates to cultured overnight at 37°C in 5% CO_2_. The cells were digested by trypsin and collected with centrifugation. Subsequently, the cells were washed twice with cooled phosphate buffer saline (PBS). After the treatment, the cells were diluted to 1 × 10^6^ cells/ml with 1 × Binding Buffer, and 100 μl of the above cell suspension was put into the falcon tube and added into 5 μl annexin V. After mixing, incubated the cells in the dark at room temperature for 25 min. The cells were washed twice with 1 × binding buffer before 5 μl of Propidium Iodide (PI) solution was added into the tube and incubated at room temperature in the dark for 15 min. Finally, the cells were diluted to 400 μl with 1 × binding buffer and detected apoptosis by flow cytometry.

### Western Blots Analysis

As described above, the cancer cells were cultured into 6-well plates for 24 h at 37°C in 5% CO_2_. Then, different concentrations of SCIP (27.8, 83.3, and 250 μg/ml) were added into the plates and cultured overnight at 37°C in 5% CO_2_. After the treatment, the cells were digested by trypsin and collected with centrifugation. The cell lysis buffer and protease inhibitor, Phenylmethanesulfonyl fluoride (PMSF), was added into the centrifuge tube with the ratio of 100:1 and then homogenized at 65 Hz in a pre-cooled homogenizer at 4°C. The homogenized samples were ultrasonicated for 5 min and centrifuged at 4°C for 10 min. After, the supernatant was collected to measure the protein concentration using the BSA kit (Beyotime, China). The above-prepared protein was separated by 12% sodium dodecyl sulfate-polyacrylamide gel electrophoresis (SDS-PAGE) at 80 V and transferred to polyvinylidene difluoride (PVDF) membrane (Pierce Biotechnology Inc, Rockford, IL) for 2.5 h at 350 mA. The PVDF membranes were blocked for 2 h at room temperature with 5% non-fat milk and were then washed three times with Tris-Tween buffered saline (TBST) (0.01% Tween-20 in TBS). The membranes were then incubated with primary antibodies including GAPDH, caspase-8, caspase-9, caspase-3, Bcl-2, Bax, Cyt-c, t-AKT, p-AKT308, t-PI3k, and p-PI3k (Cell Signaling Technol, USA) overnight at 4°C, respectively. The primary antibody, GAPDH (Santa Cruz Company, Dallas, TX), was used as the reference protein. Following incubation, the membranes were washed three times in TBST and incubated with a secondary antibody (Abcam, UK) for 1 h at room temperature. Finally, the enhanced chemiluminescence (Pierce Biotechnology, Rock-ford, IL, USA) was added to the membrane to detect the fluorescence signals. All blots were quantified using ImageJ software (V1.53e, National Institutes of Health, USA).

### PI3K Inhibitor Treatment

MCF-7 cells (1 × 106 cells/ml) were cultured into 6-well plates for 24 h at 37°C in 5% CO_2_. Then, the fresh medium was replaced and pretreated the cells with 100 nm wortmannin (PI3K inhibitor, Beyotime, China) for 1 h by following the manufacturer's instructions. At the end of the treatment, the cells were washed three times with PBS. Subsequently, the fresh medium with different concentrations of SCIP (27.8, 83.3, and 250 μg/ml) was added into the cells and continued to culture for 24 h. Finally, the cells were collected to perform WB analysis as described above.

### Statistical Analysis

All experiments were repeated at least three times. Measurement data were expressed as mean ± SD and analyzed by Student's test. A value of *P* < 0.05 was considered statistically significant.

## Data Availability Statement

The original contributions presented in the study are included in the article/Supplementary Material, further inquiries can be directed to the corresponding author/s.

## Author Contributions

WW, S-HJ, and Y-WZ: conceptualization. WW, X-MF, and X-PZ: methodology. WW: software and investigation. X-JZ, ZZ, and J-XZ: validation. WW and S-HJ: writing—original draft preparation. X-MF and Y-WZ: writing—review and editing. Y-WZ: supervision. All authors have read and agreed to the published version of the manuscript.

## Conflict of Interest

WW, X-PZ, ZZ, X-JZ and J-XZ were employed by company Shandong Tianjiu Industry Group. The remaining authors declare that the research was conducted in the absence of any commercial or financial relationships that could be construed as a potential conflict of interest.

## Publisher's Note

All claims expressed in this article are solely those of the authors and do not necessarily represent those of their affiliated organizations, or those of the publisher, the editors and the reviewers. Any product that may be evaluated in this article, or claim that may be made by its manufacturer, is not guaranteed or endorsed by the publisher.
