# Supplementary figures and images for: Sea Cucumber Intestinal Peptide Induces the Apoptosis of MCF-7 Cells by Inhibiting PI3K/AKT Pathway
Source: Front Nutr. 2021 Dec 14;8:763692. doi: 10.3389/fnut.2021.763692 (PMC8713759; doi:10.3389/fnut.2021.763692)

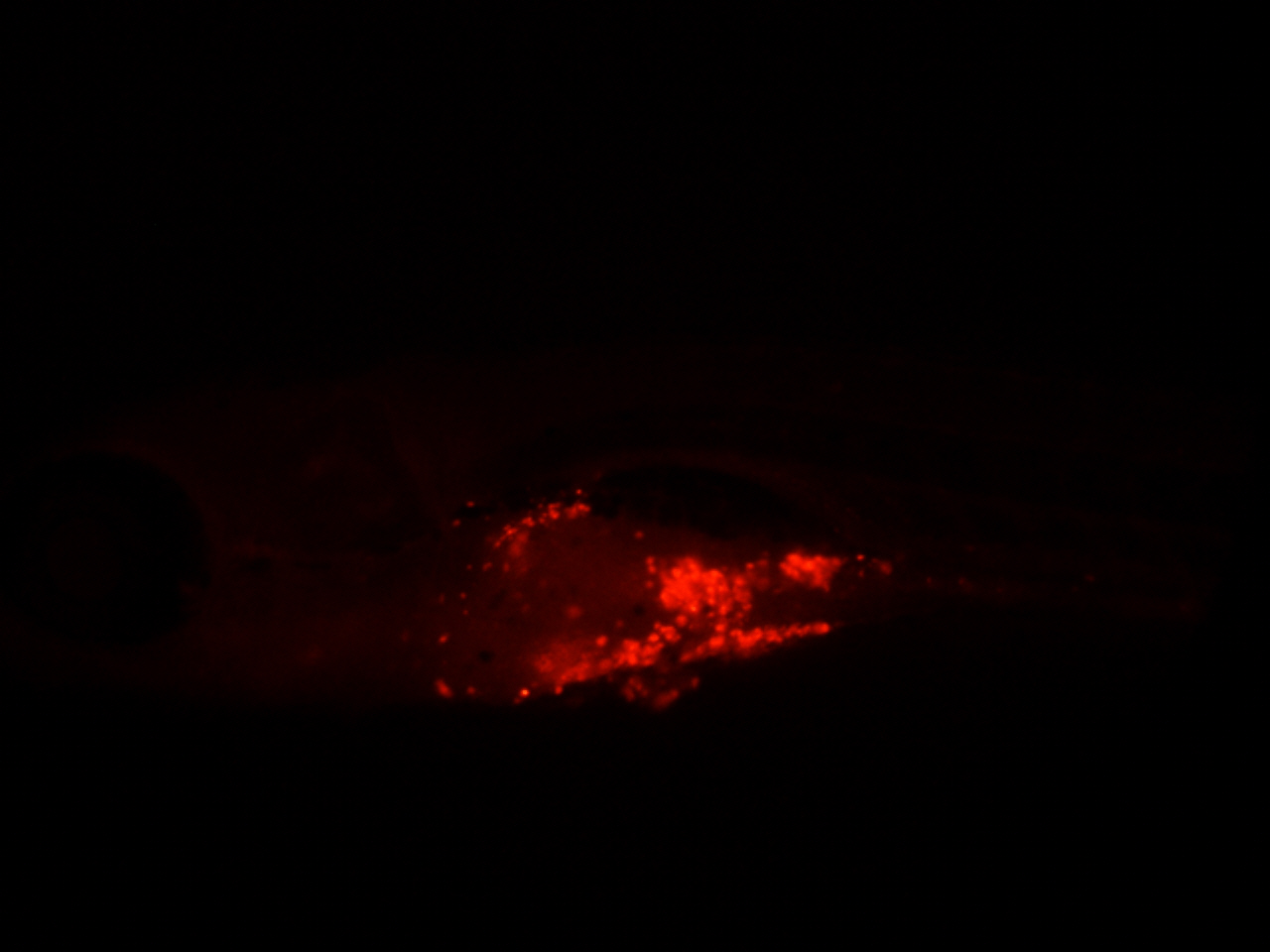

Supplement: Supplementary file 1 [file Data_Sheet_1.ZIP › 250 3/1.jpg]

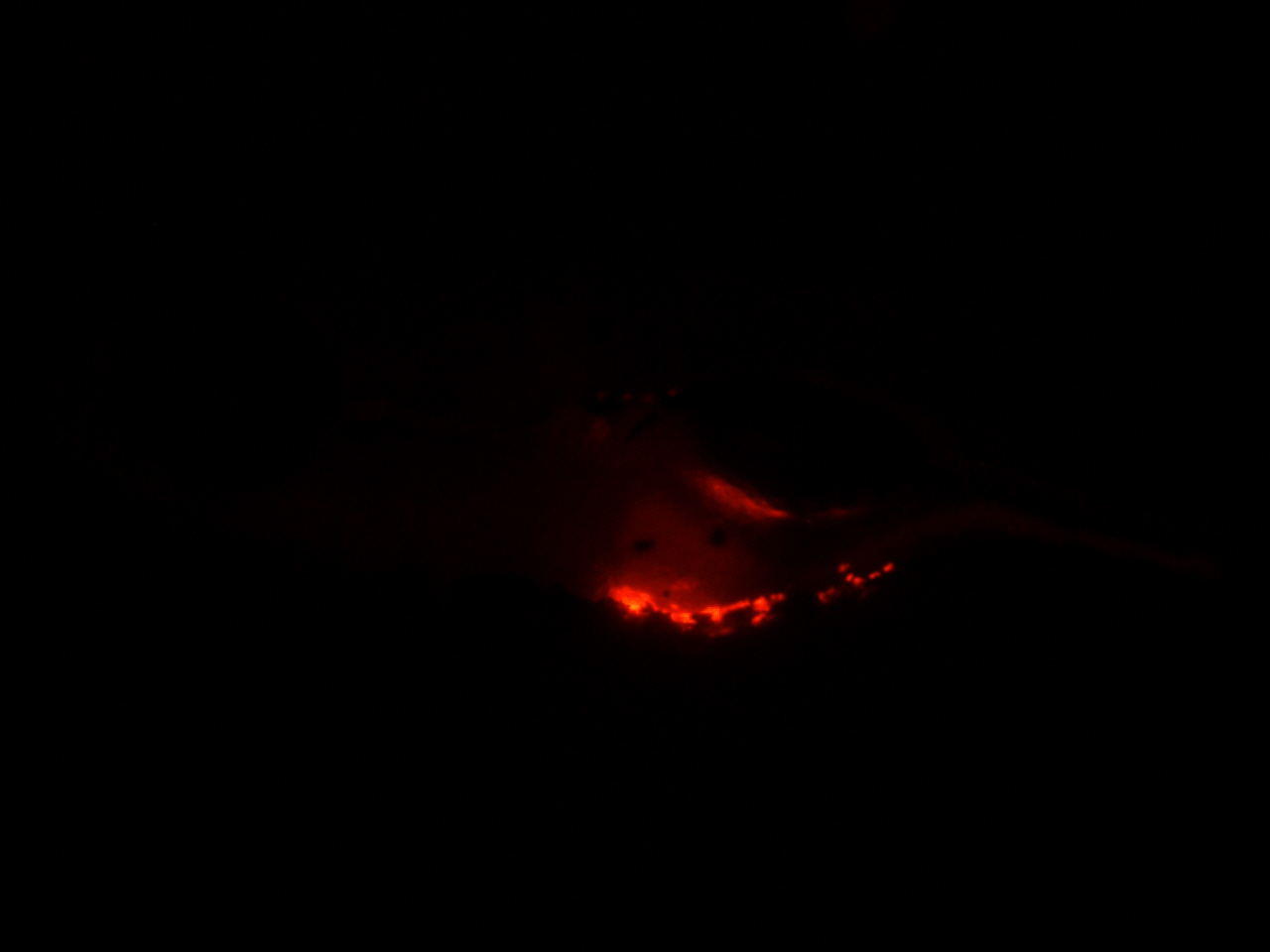

Supplement: Supplementary file 1 [file Data_Sheet_1.ZIP › 250 3/10.jpg]

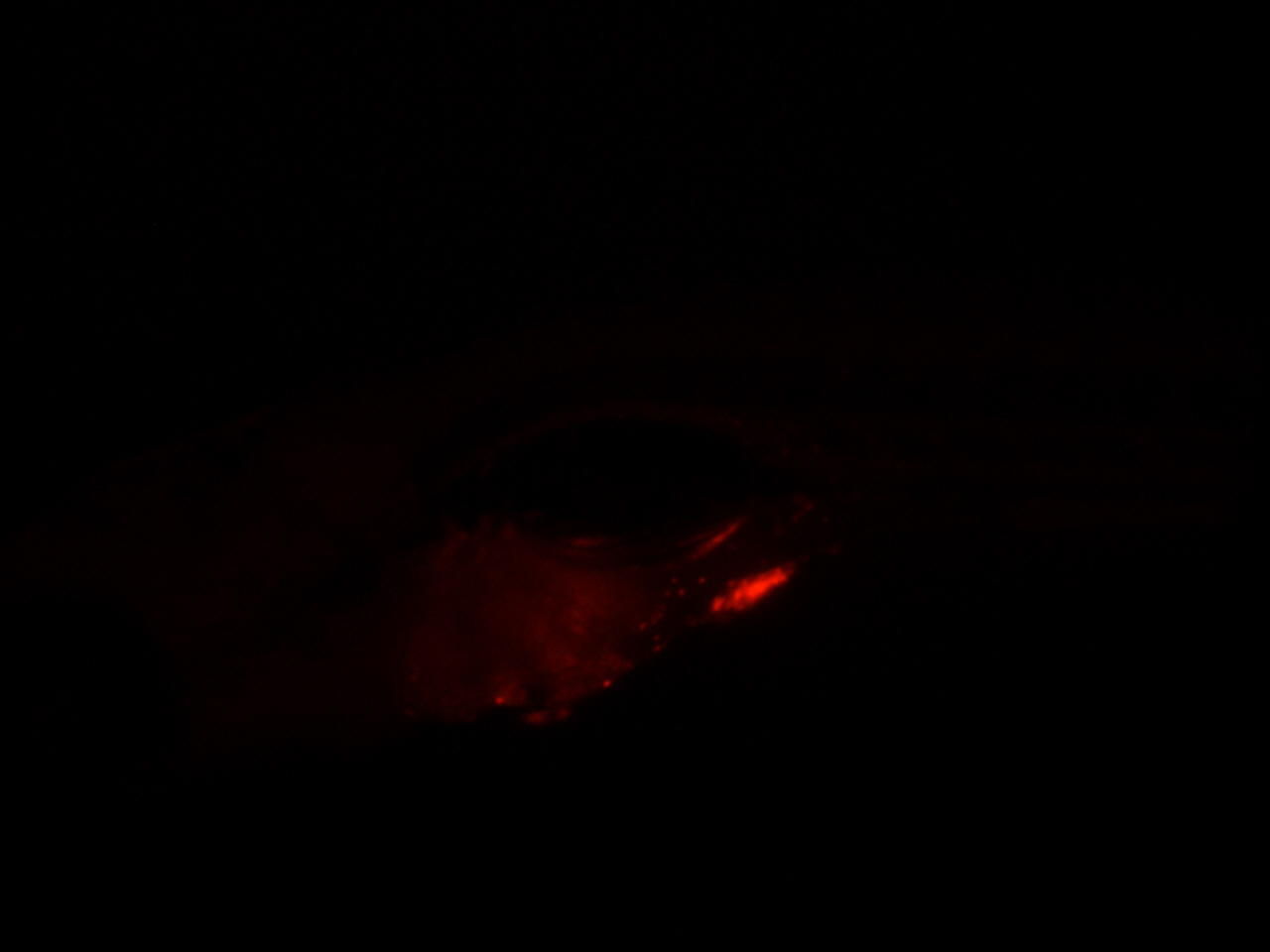

Supplement: Supplementary file 1 [file Data_Sheet_1.ZIP › 250 3/11.jpg]

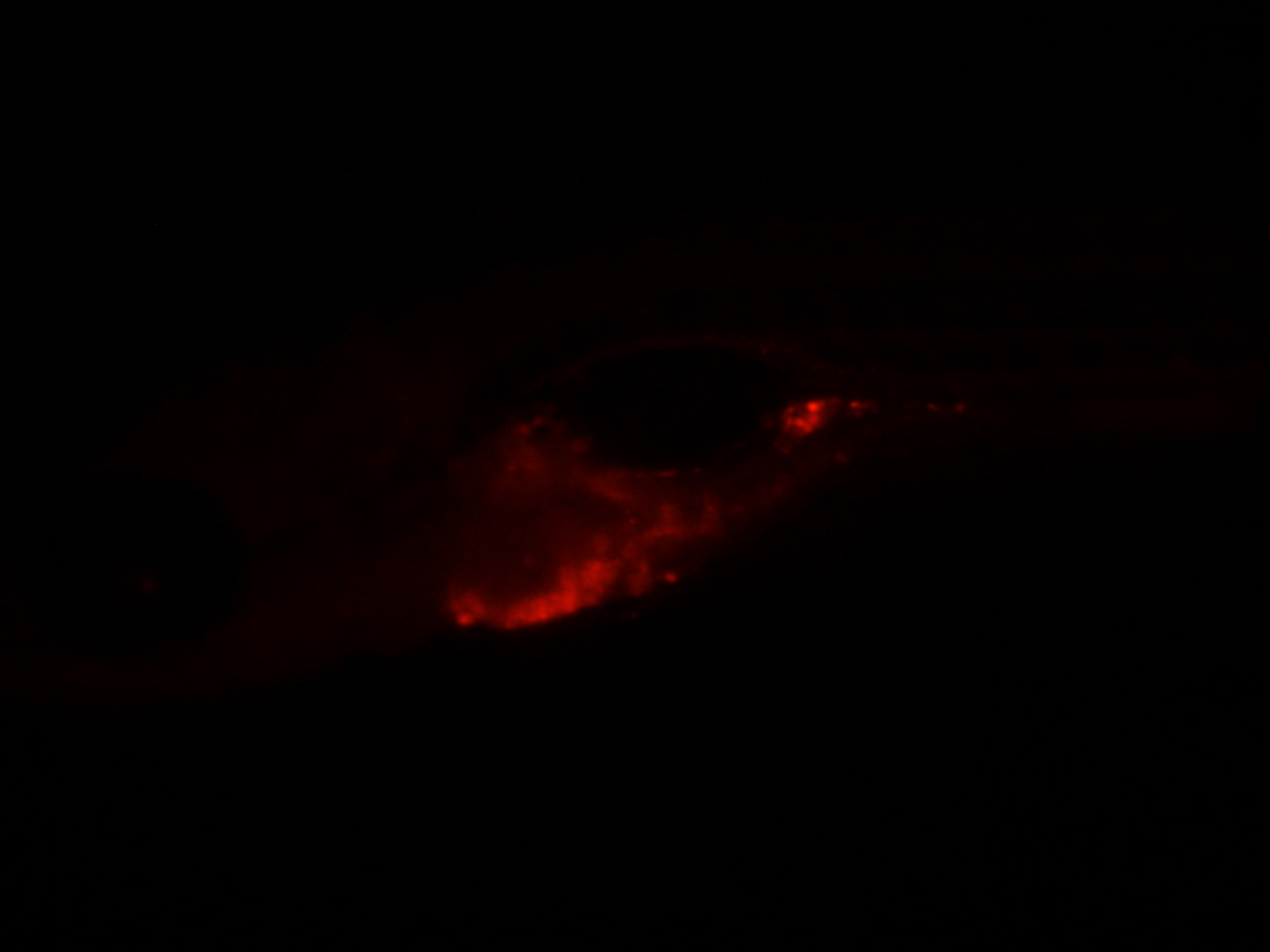

Supplement: Supplementary file 1 [file Data_Sheet_1.ZIP › 250 3/12.jpg]

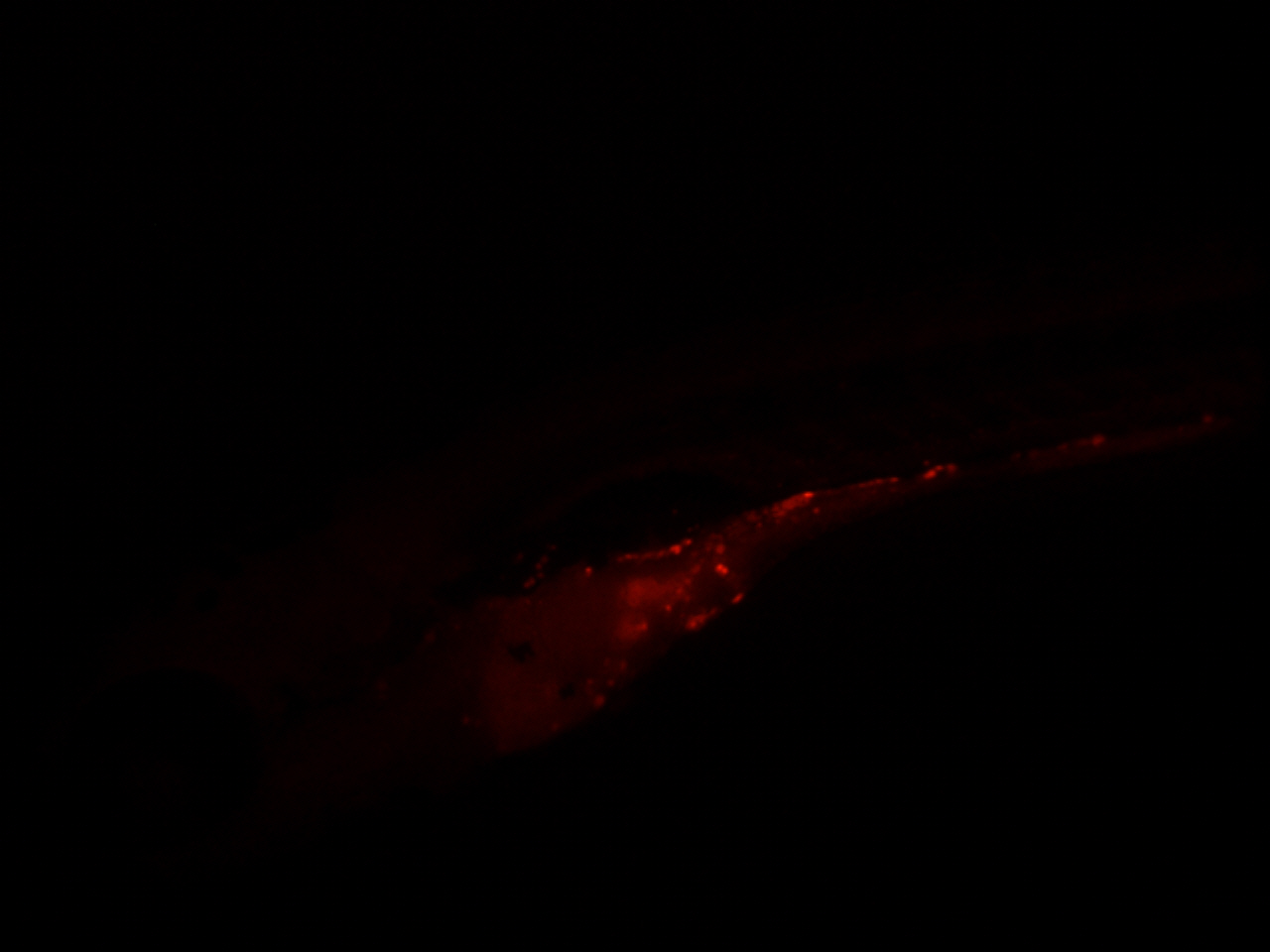

Supplement: Supplementary file 1 [file Data_Sheet_1.ZIP › 250 3/13.jpg]

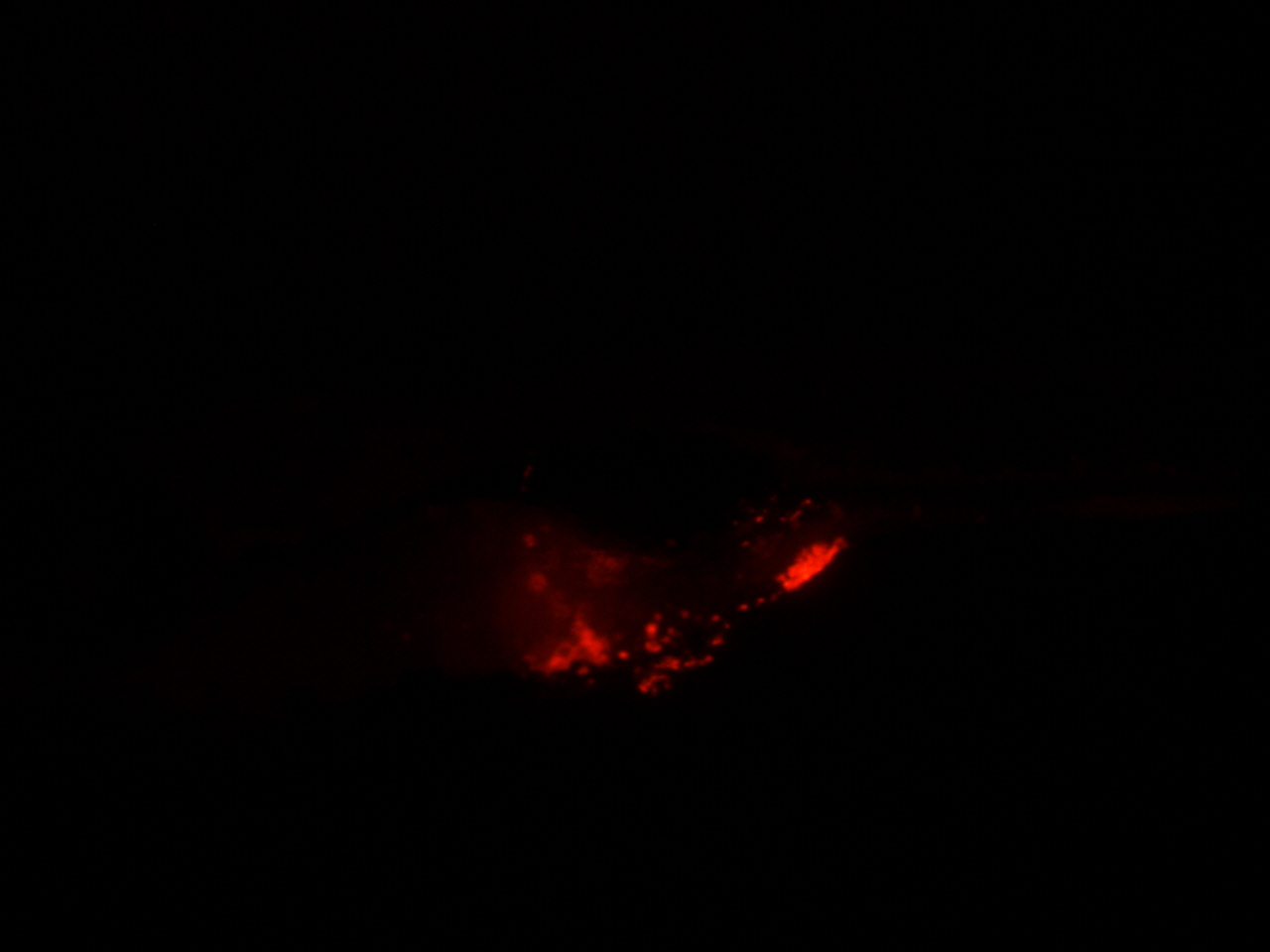

Supplement: Supplementary file 1 [file Data_Sheet_1.ZIP › 250 3/14.jpg]

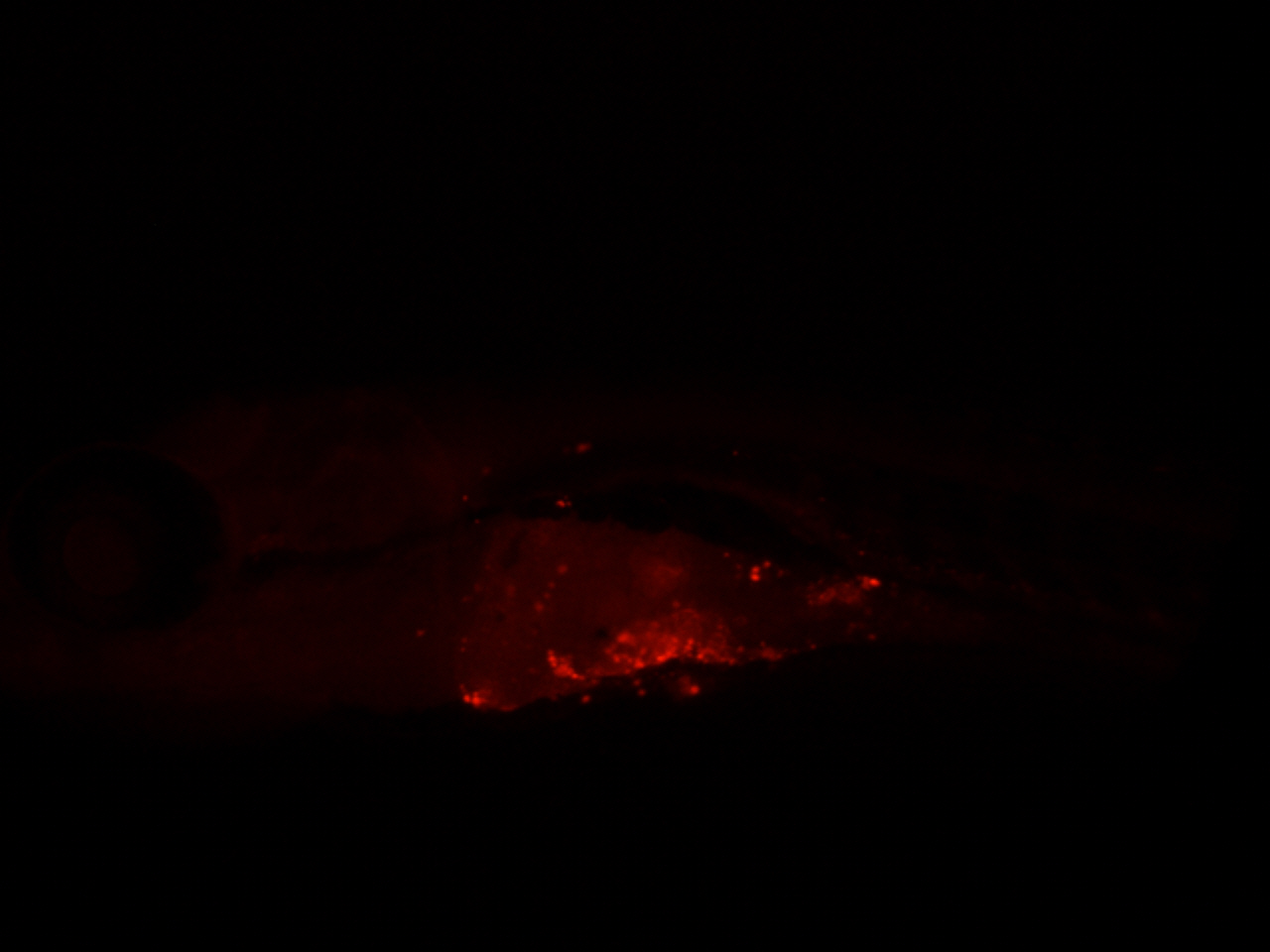

Supplement: Supplementary file 1 [file Data_Sheet_1.ZIP › 250 3/2.jpg]

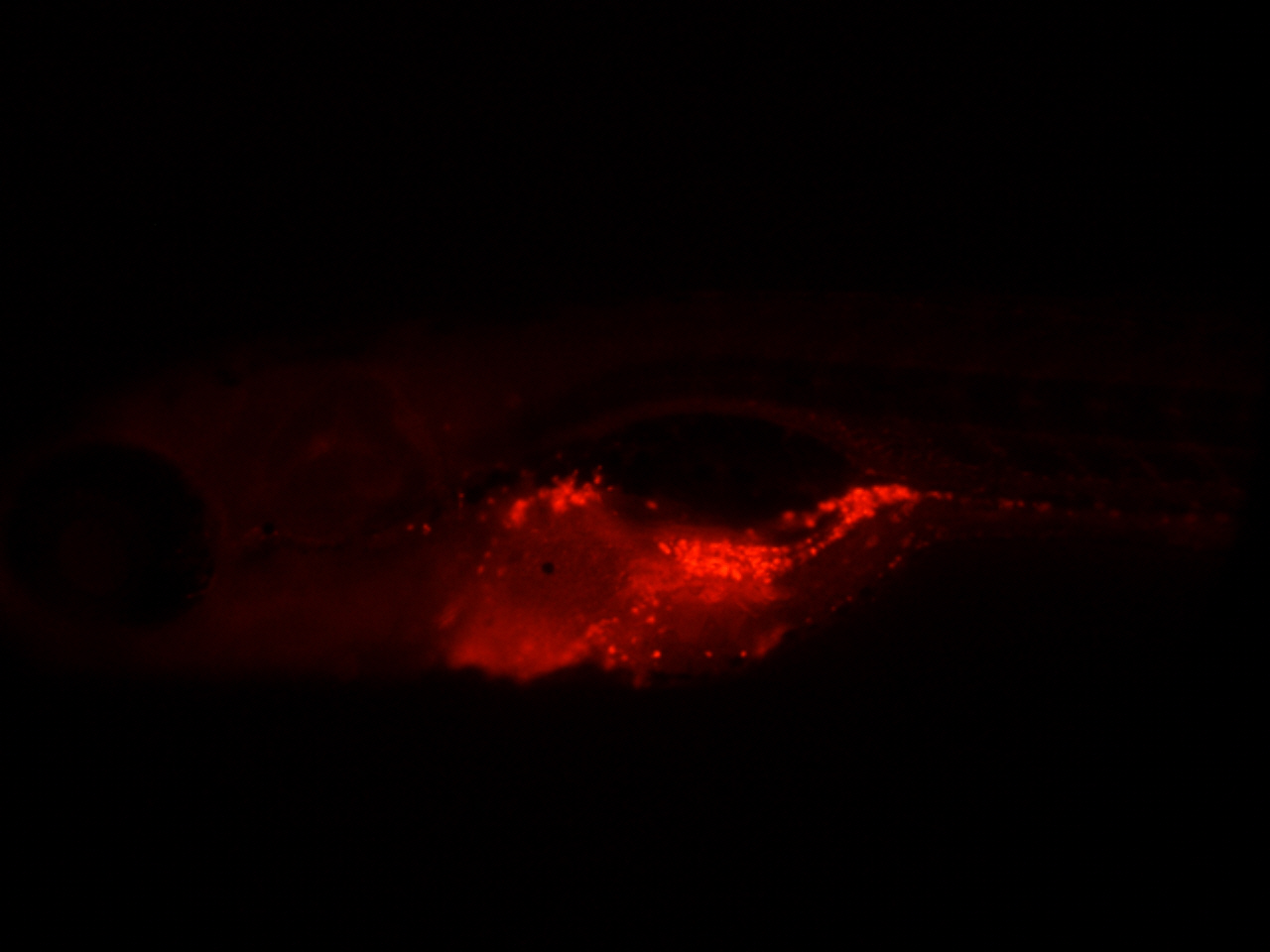

Supplement: Supplementary file 1 [file Data_Sheet_1.ZIP › 250 3/3.jpg]

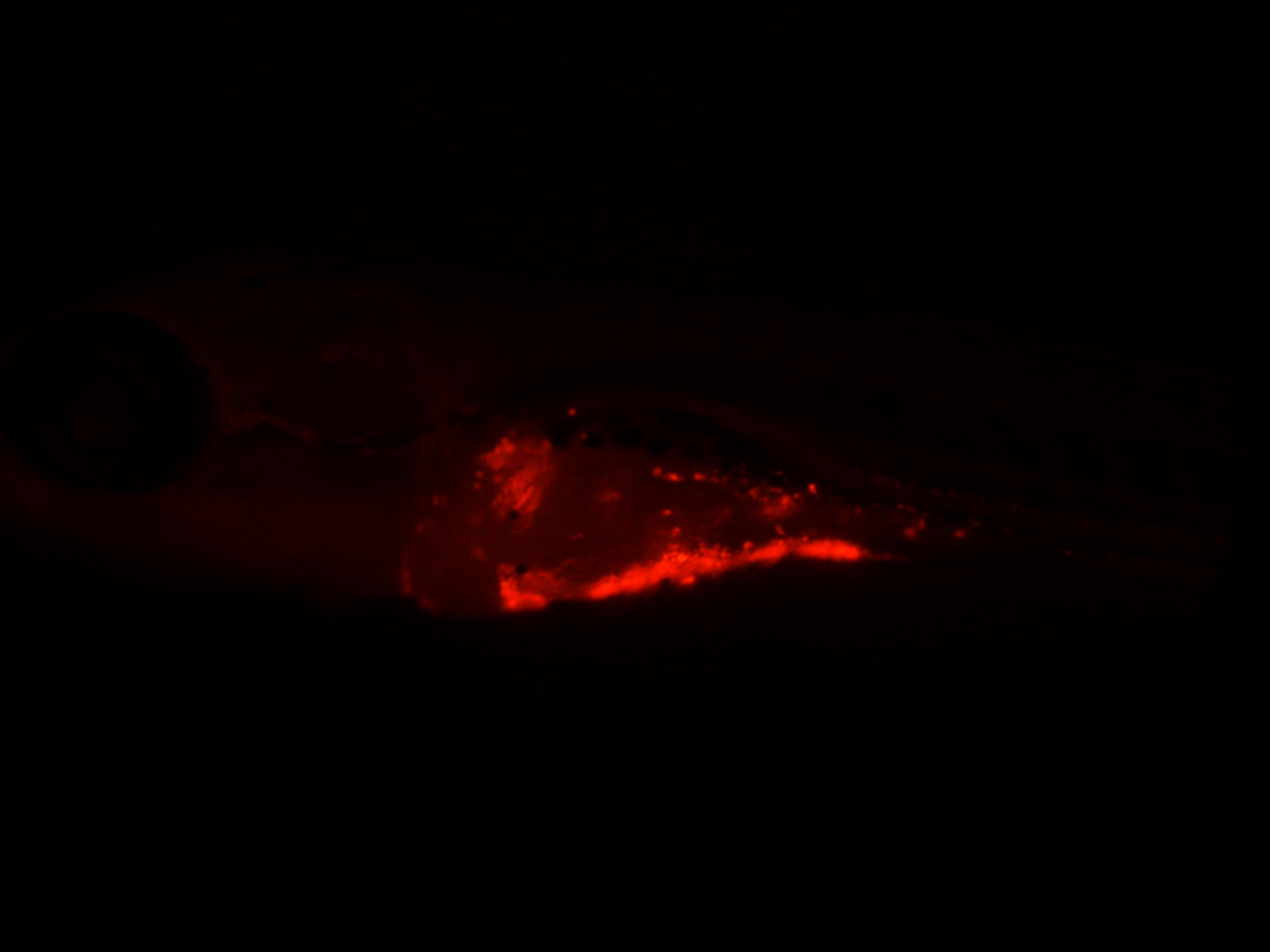

Supplement: Supplementary file 1 [file Data_Sheet_1.ZIP › 250 3/4.jpg]

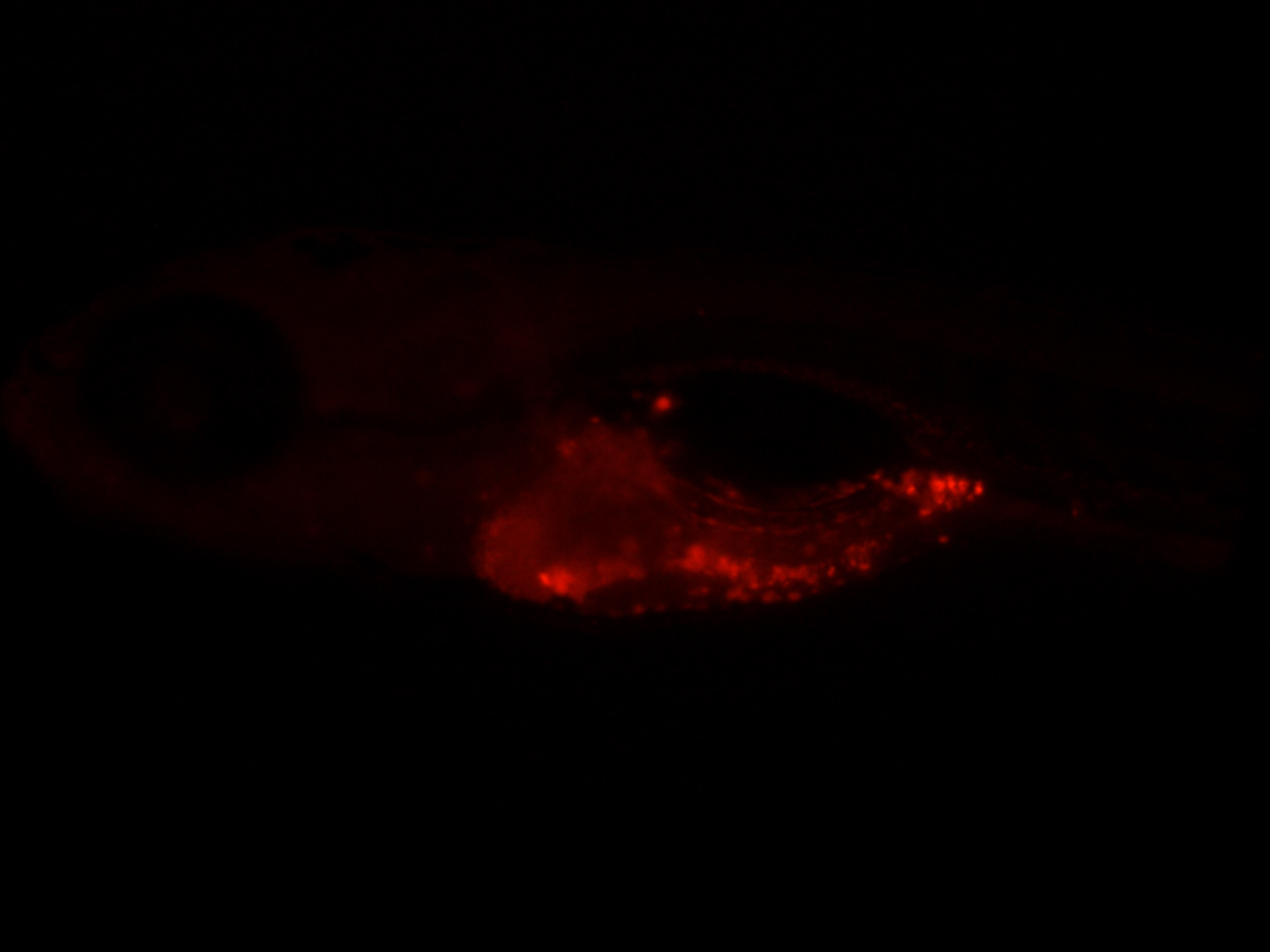

Supplement: Supplementary file 1 [file Data_Sheet_1.ZIP › 250 3/5.jpg]

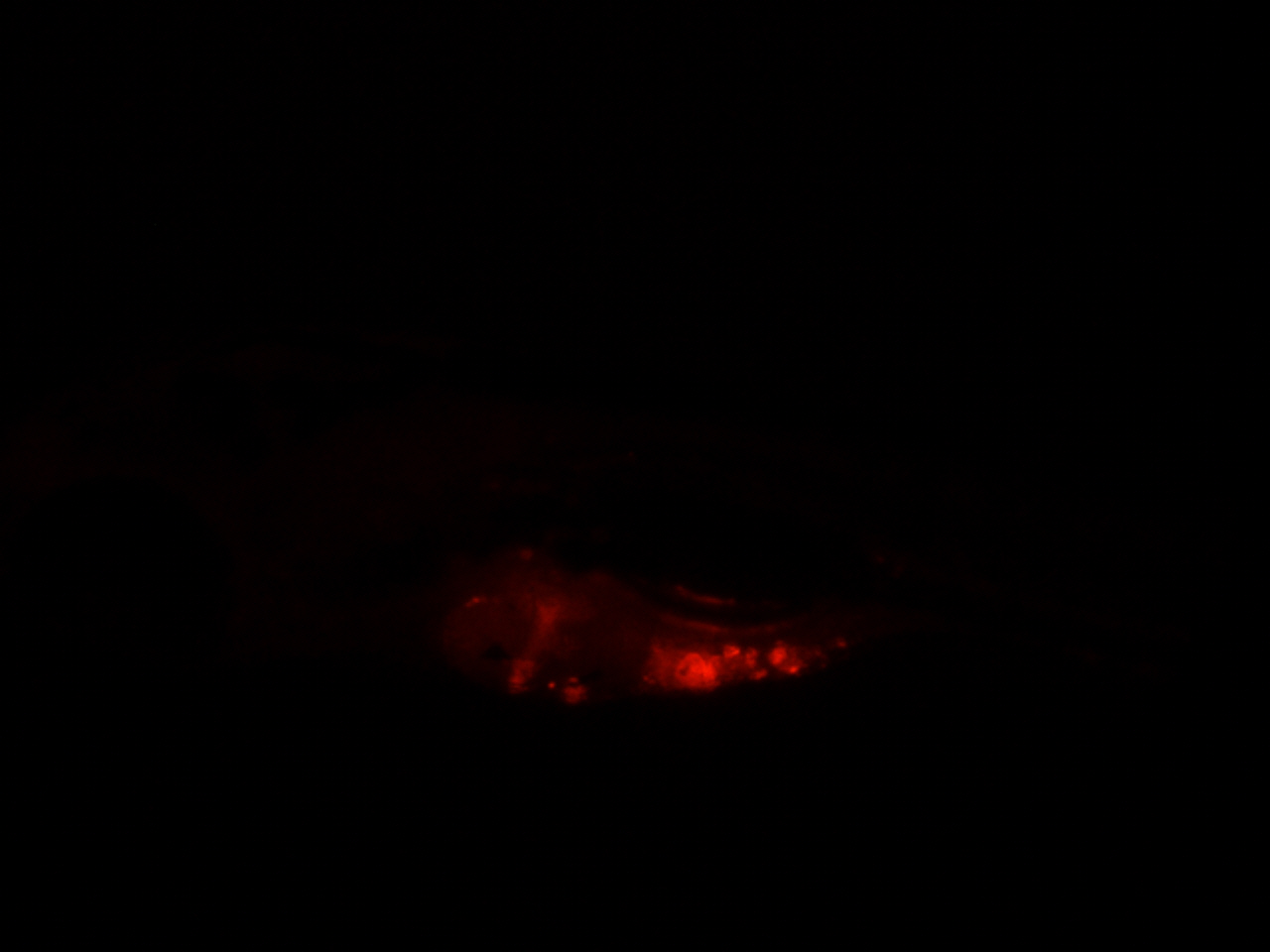

Supplement: Supplementary file 1 [file Data_Sheet_1.ZIP › 250 3/6.jpg]

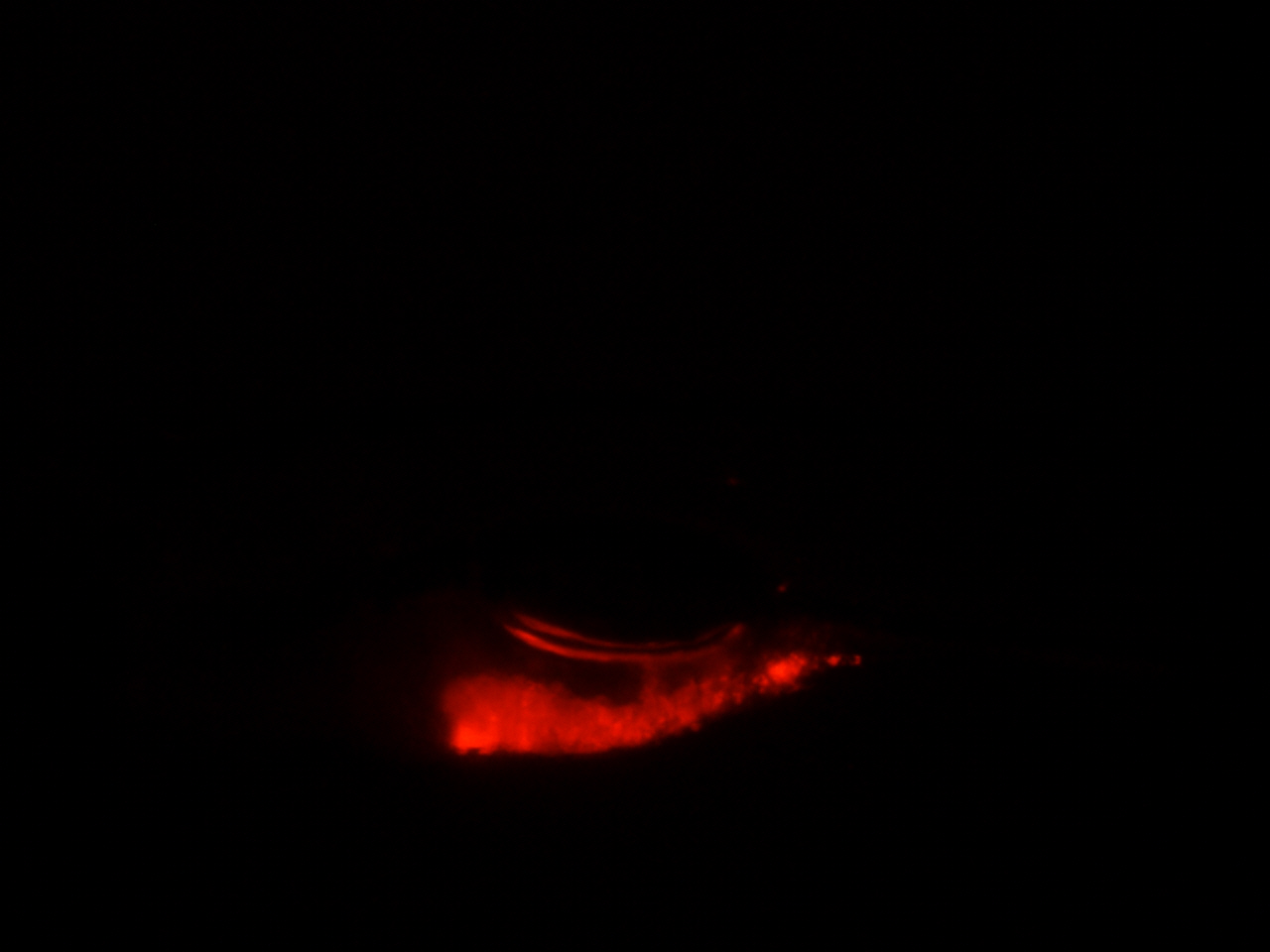

Supplement: Supplementary file 1 [file Data_Sheet_1.ZIP › 250 3/7.jpg]

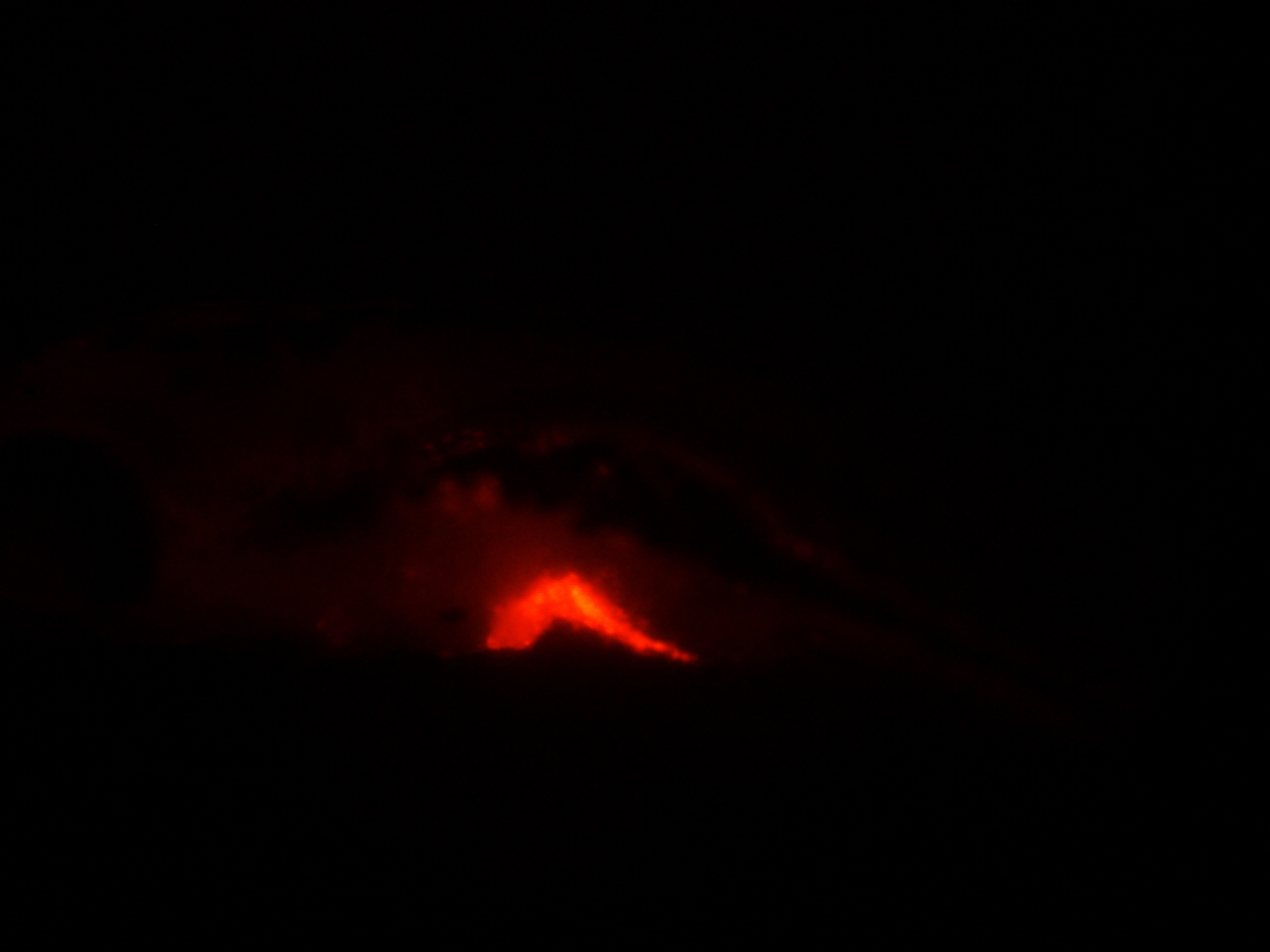

Supplement: Supplementary file 1 [file Data_Sheet_1.ZIP › 250 3/8.jpg]

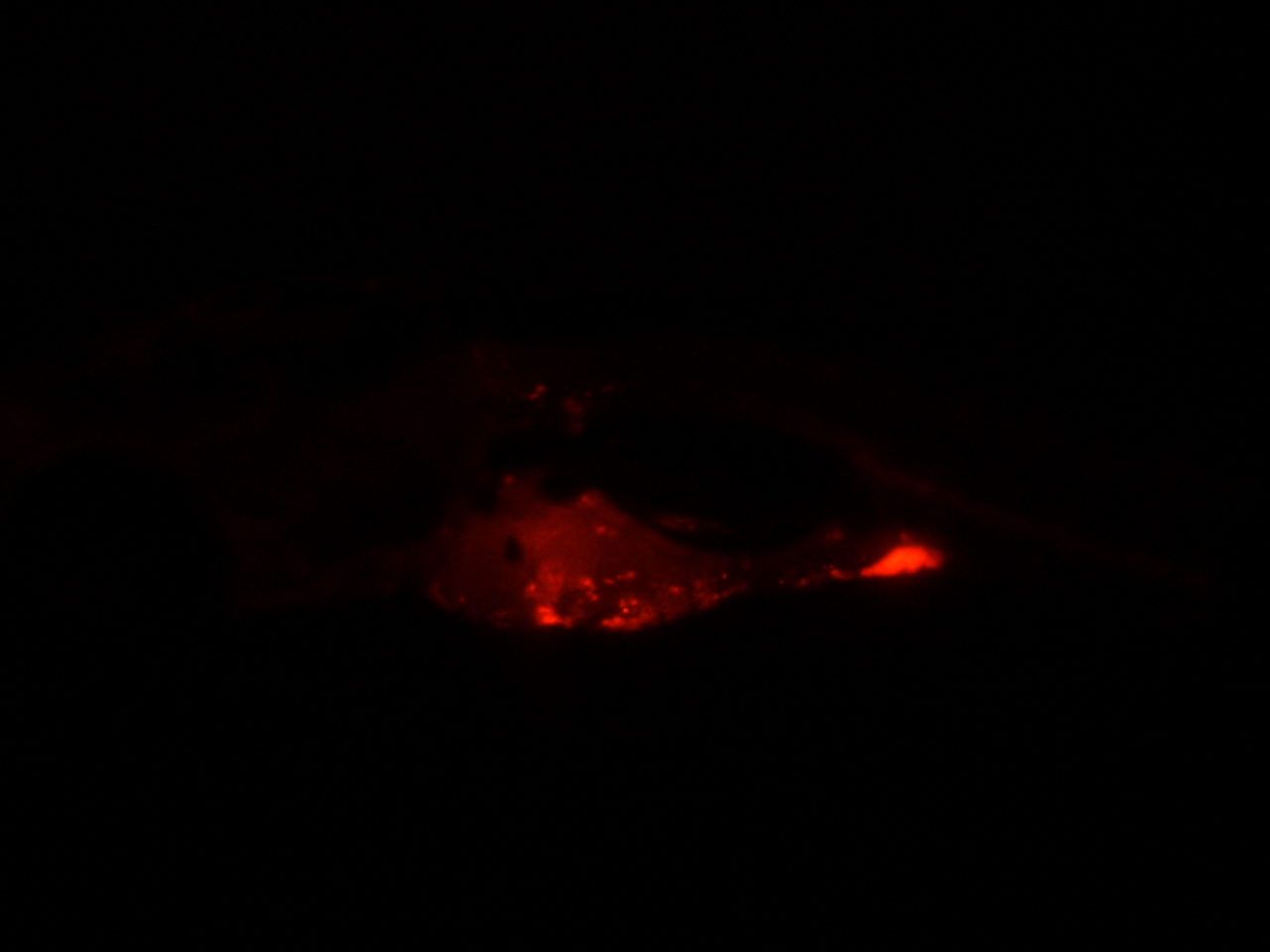

Supplement: Supplementary file 1 [file Data_Sheet_1.ZIP › 250 3/9.jpg]

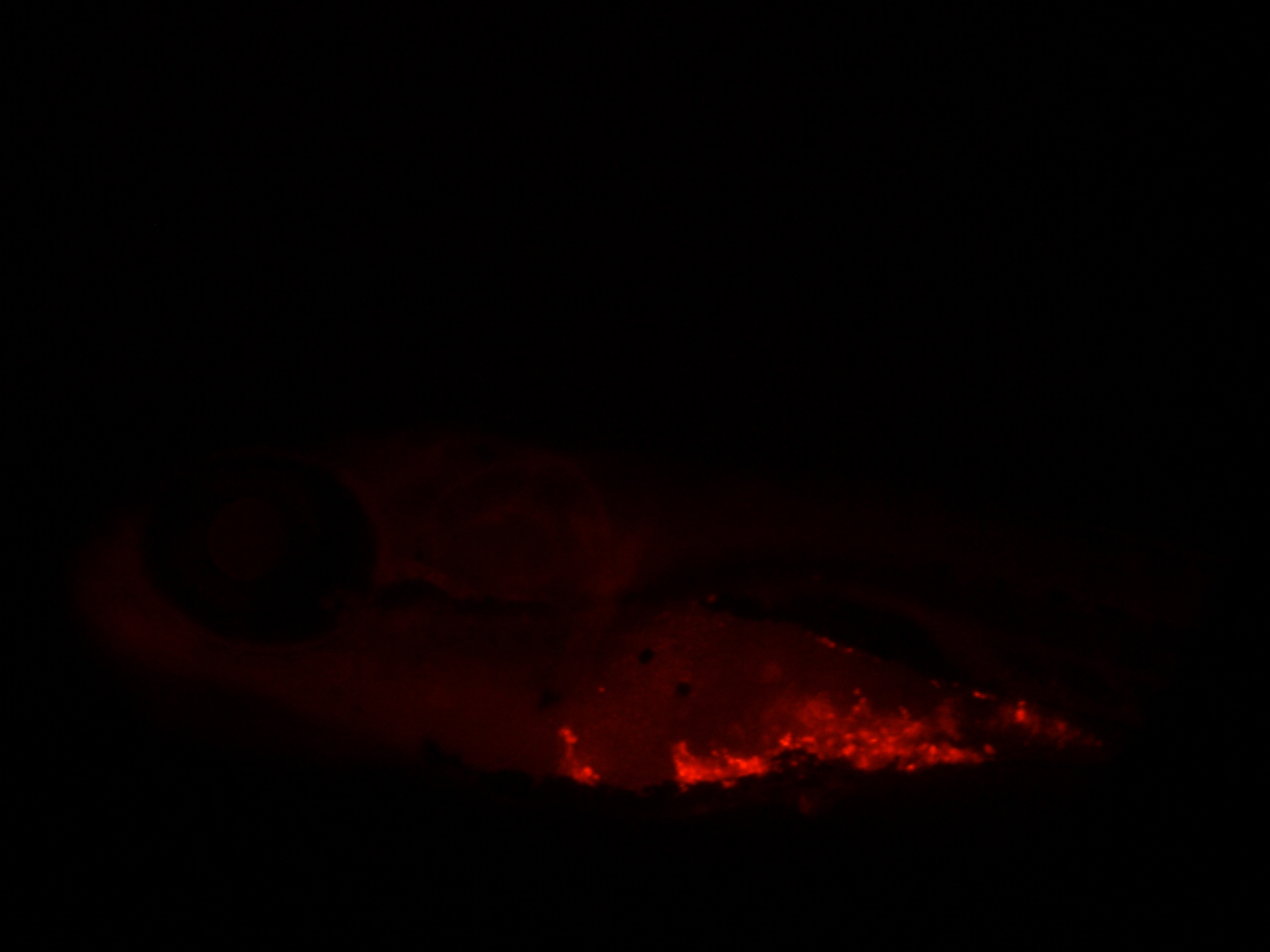

Supplement: Supplementary file 1 [file Data_Sheet_1.ZIP › 250 9/1.jpg]

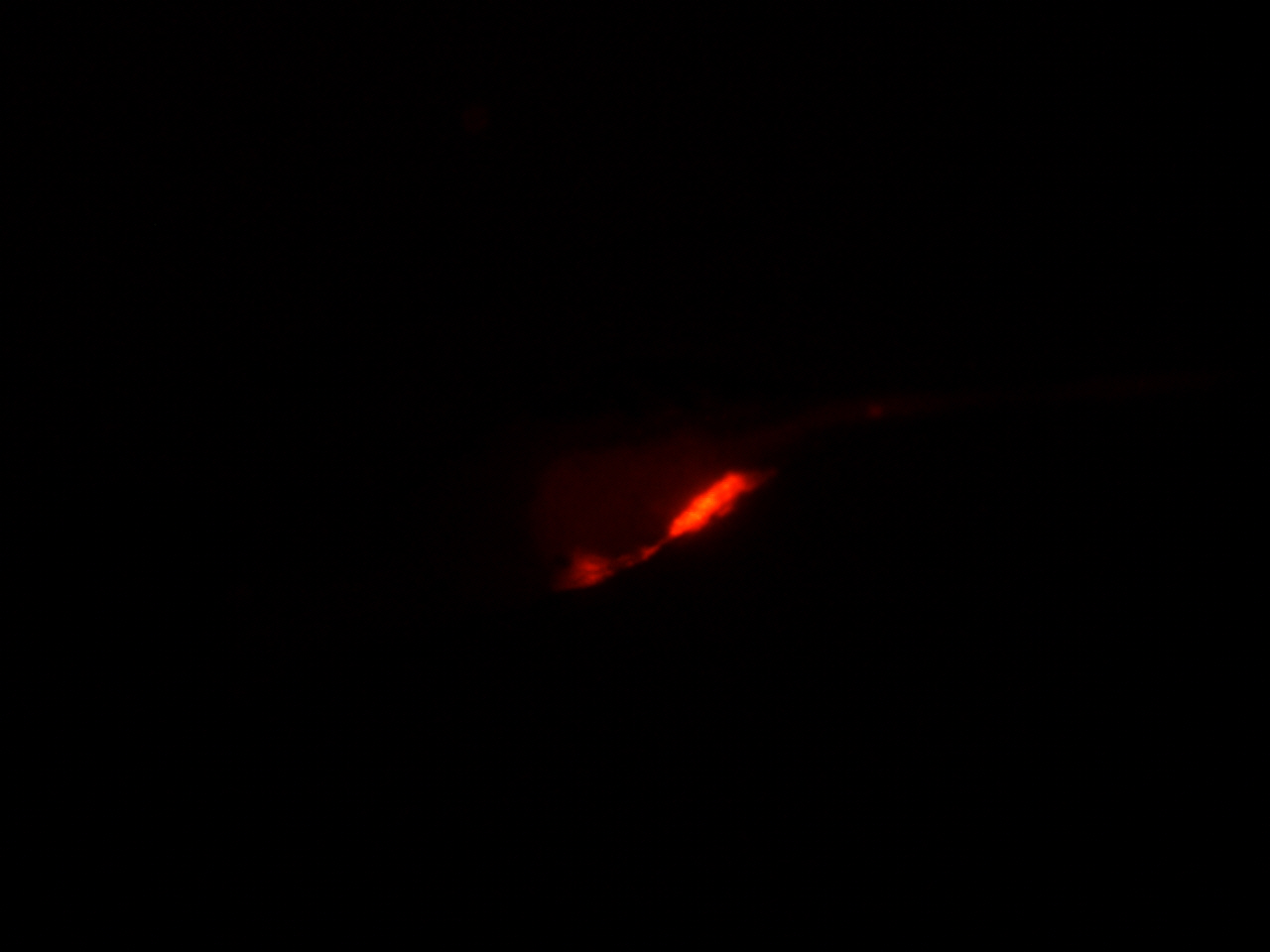

Supplement: Supplementary file 1 [file Data_Sheet_1.ZIP › 250 9/10.jpg]

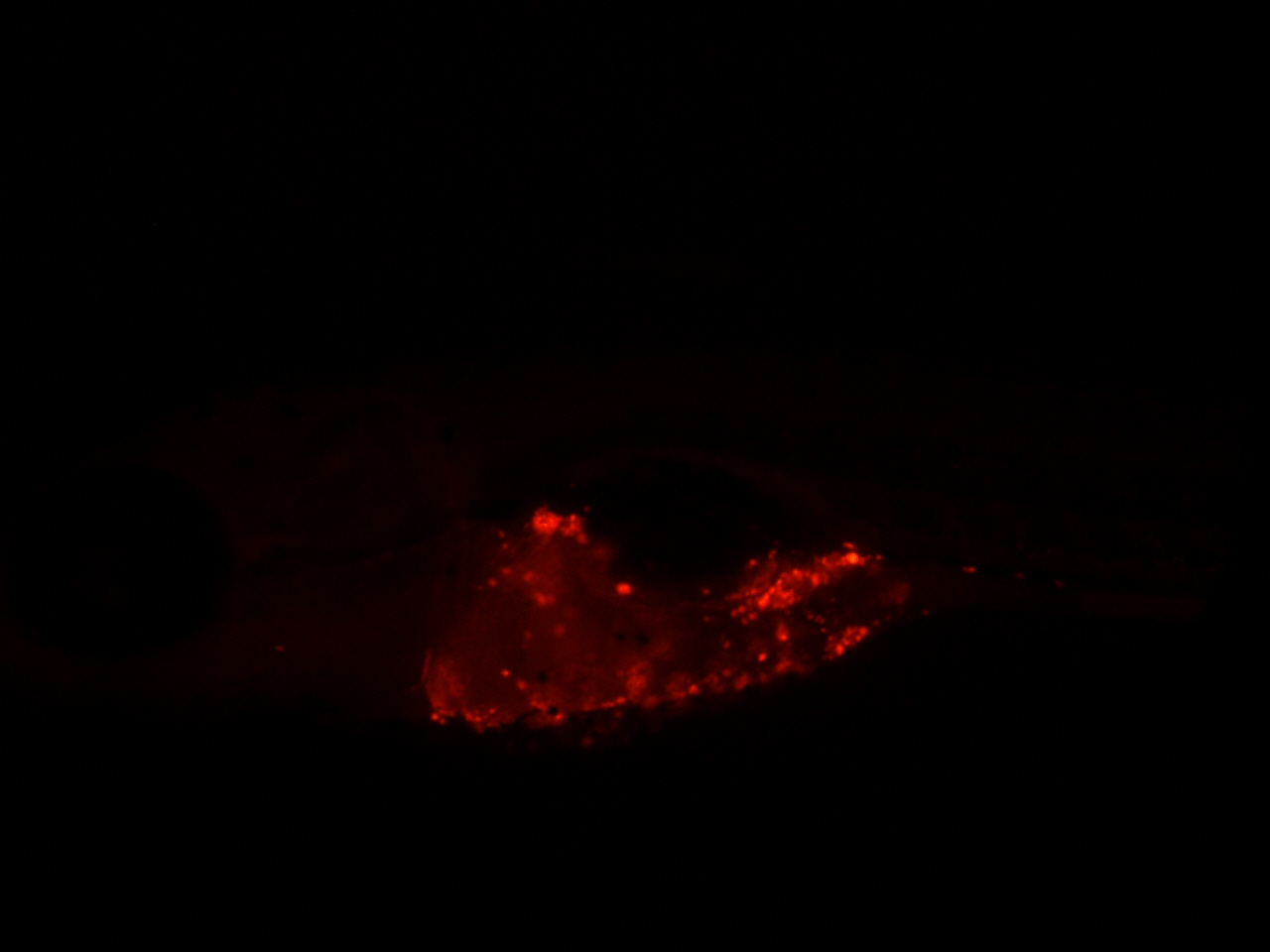

Supplement: Supplementary file 1 [file Data_Sheet_1.ZIP › 250 9/11.jpg]

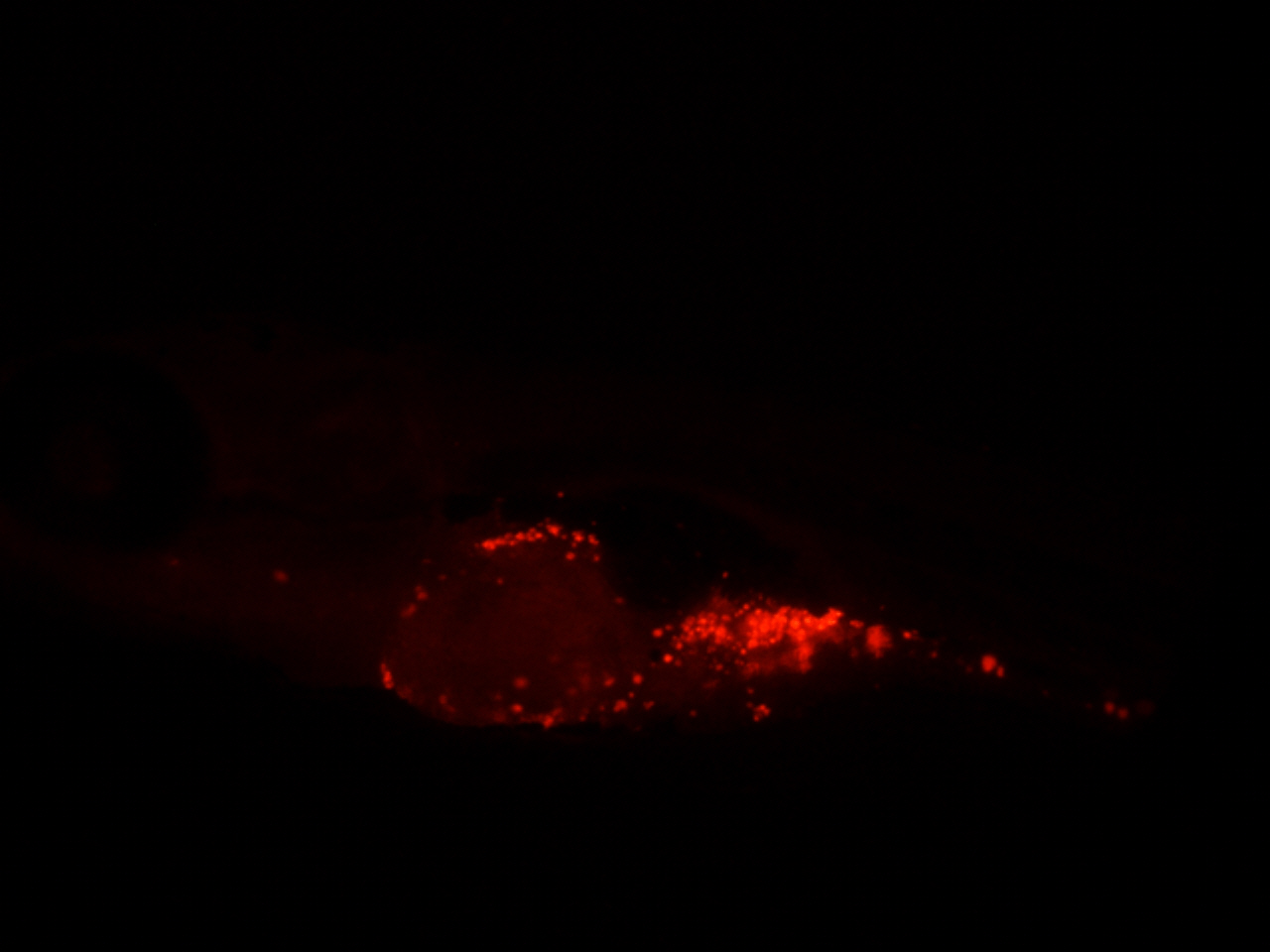

Supplement: Supplementary file 1 [file Data_Sheet_1.ZIP › 250 9/12.jpg]

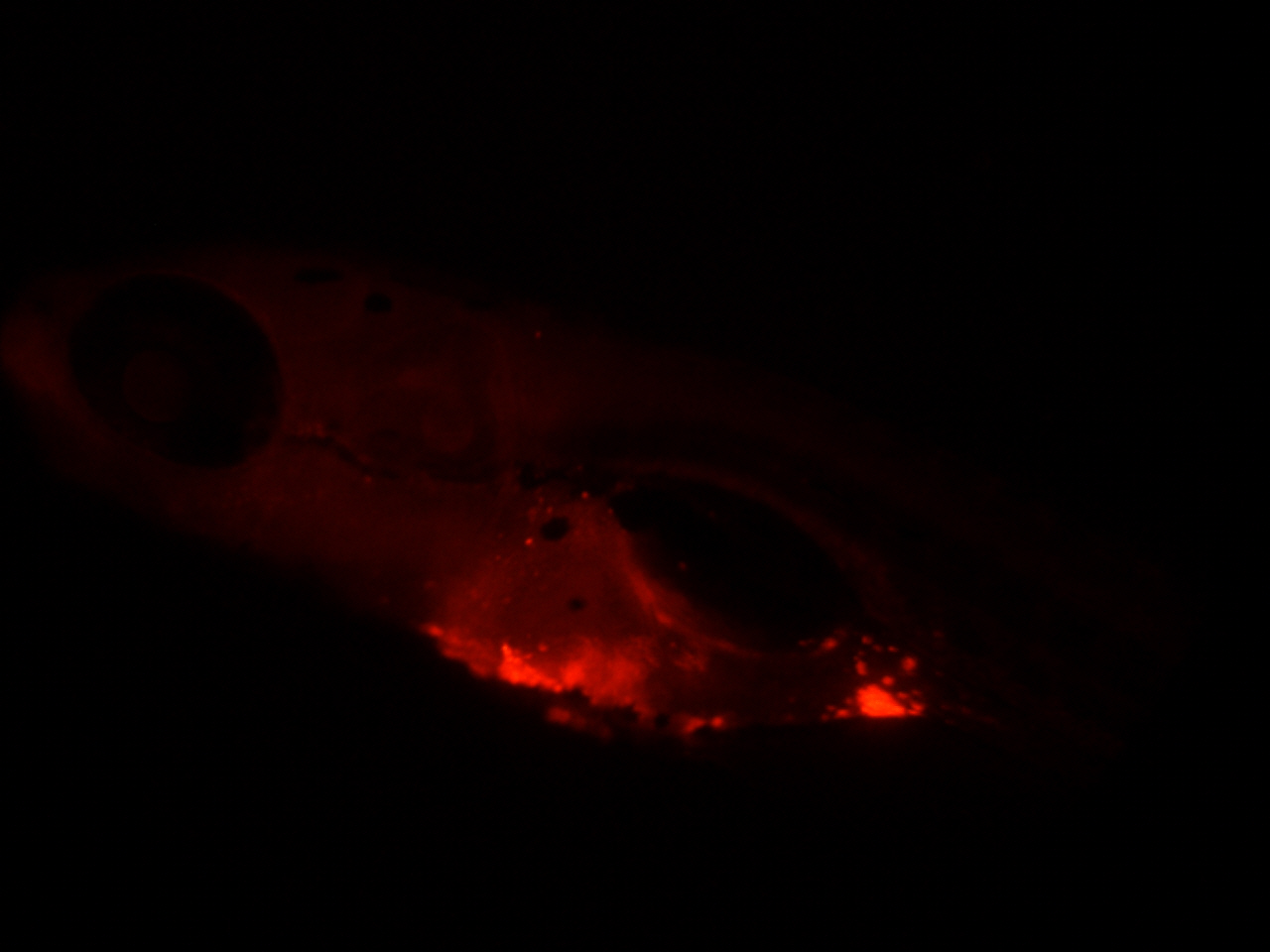

Supplement: Supplementary file 1 [file Data_Sheet_1.ZIP › 250 9/13.jpg]

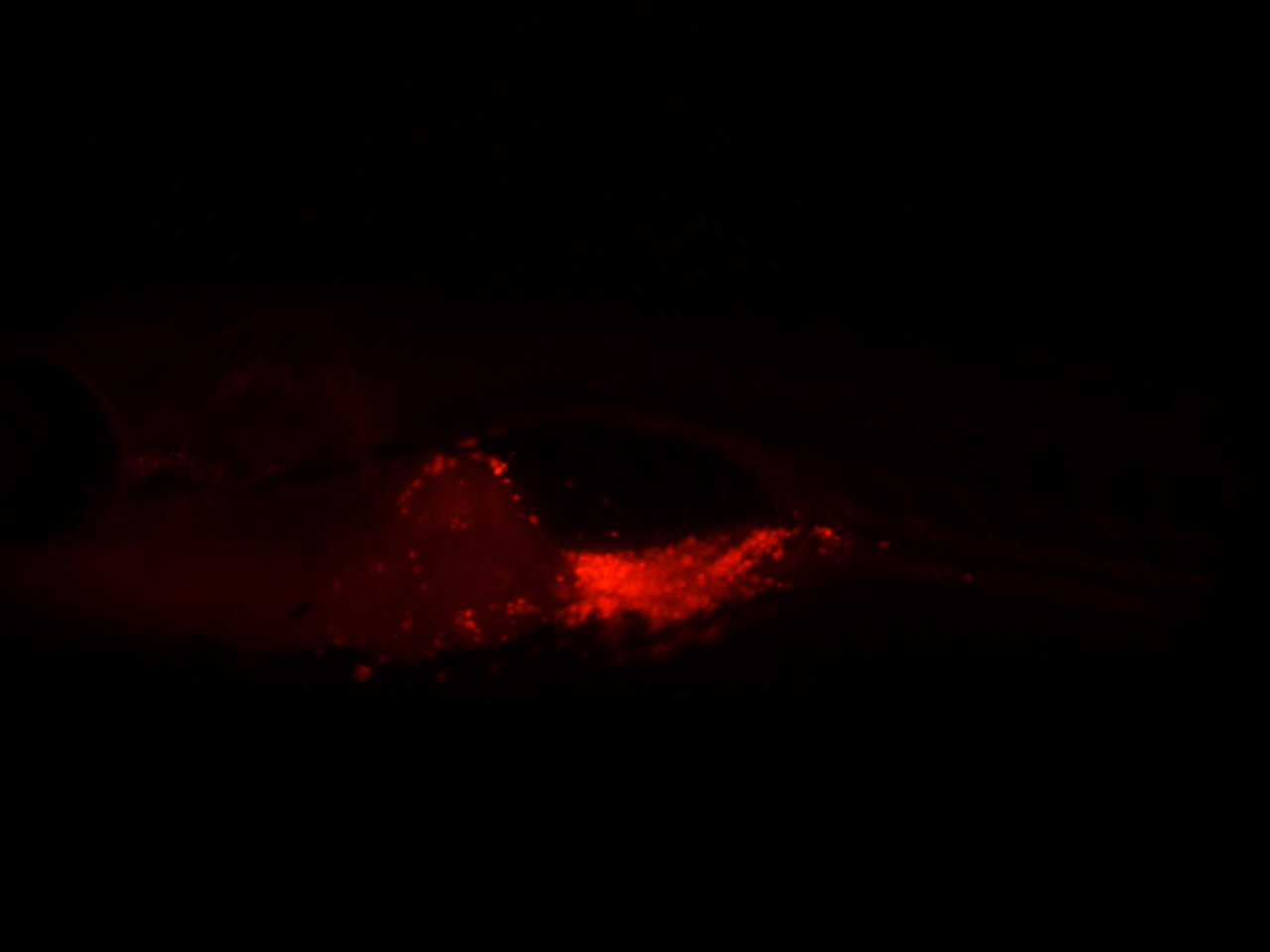

Supplement: Supplementary file 1 [file Data_Sheet_1.ZIP › 250 9/14.jpg]

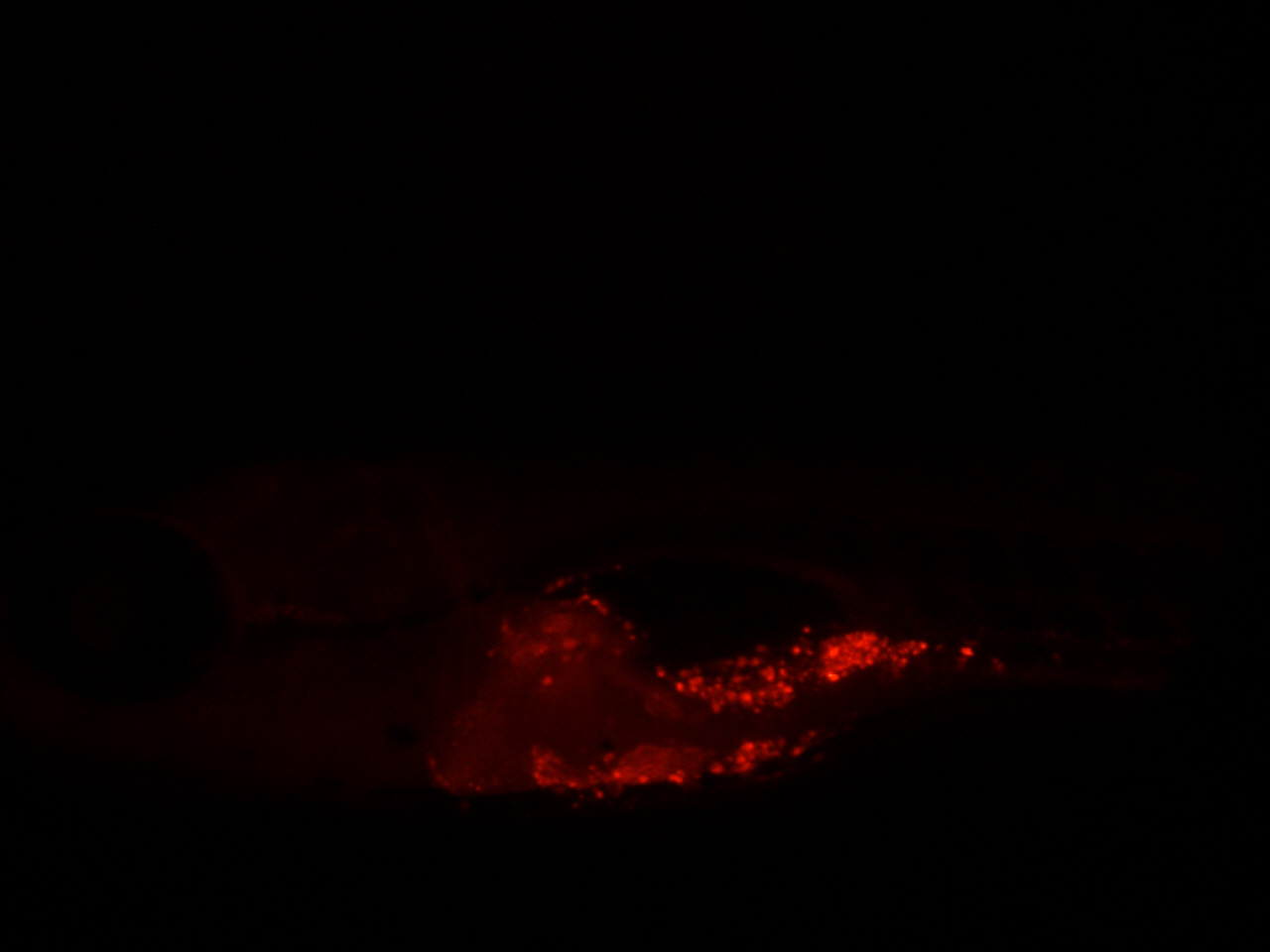

Supplement: Supplementary file 1 [file Data_Sheet_1.ZIP › 250 9/2.jpg]

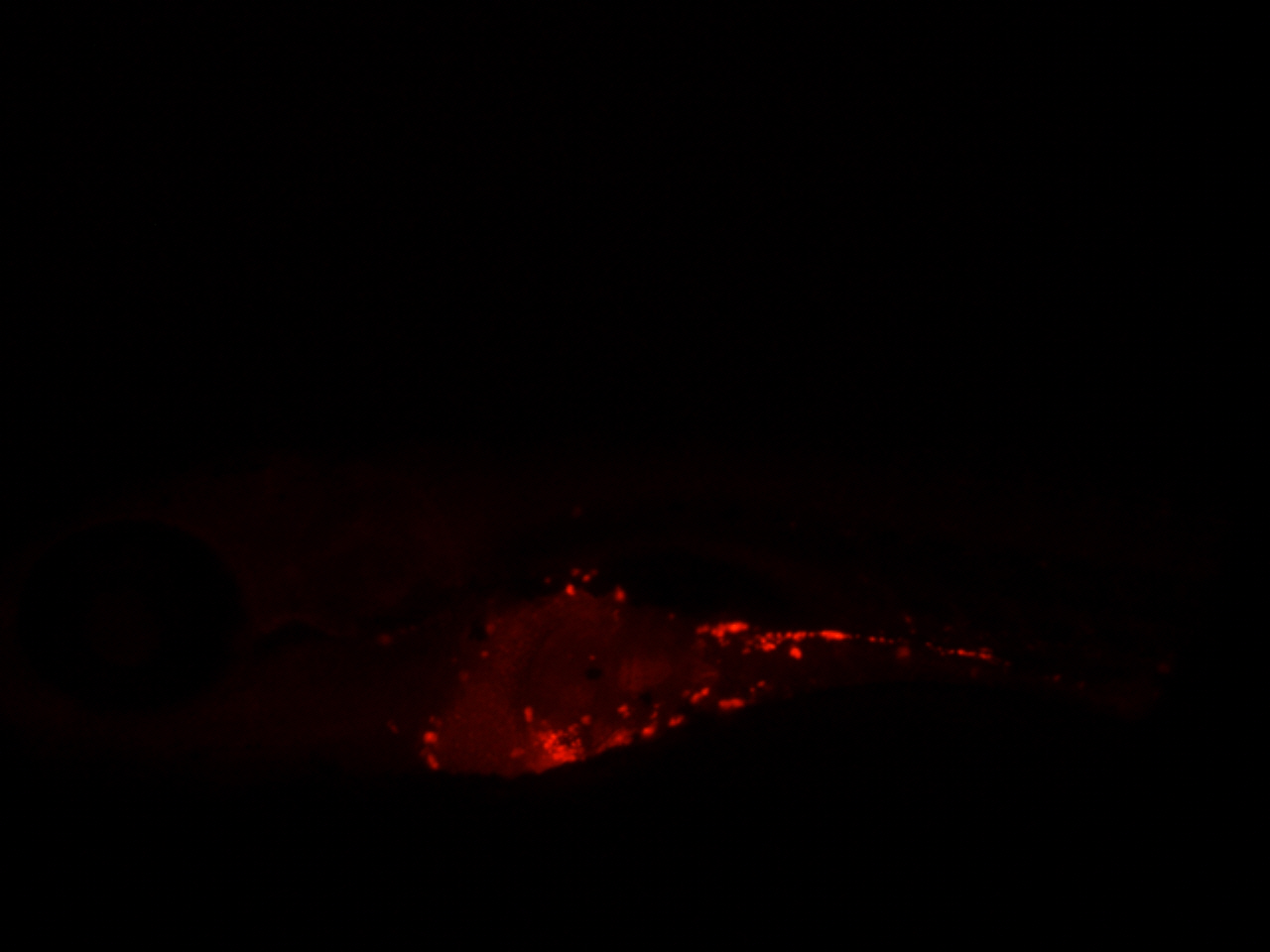

Supplement: Supplementary file 1 [file Data_Sheet_1.ZIP › 250 9/3.jpg]

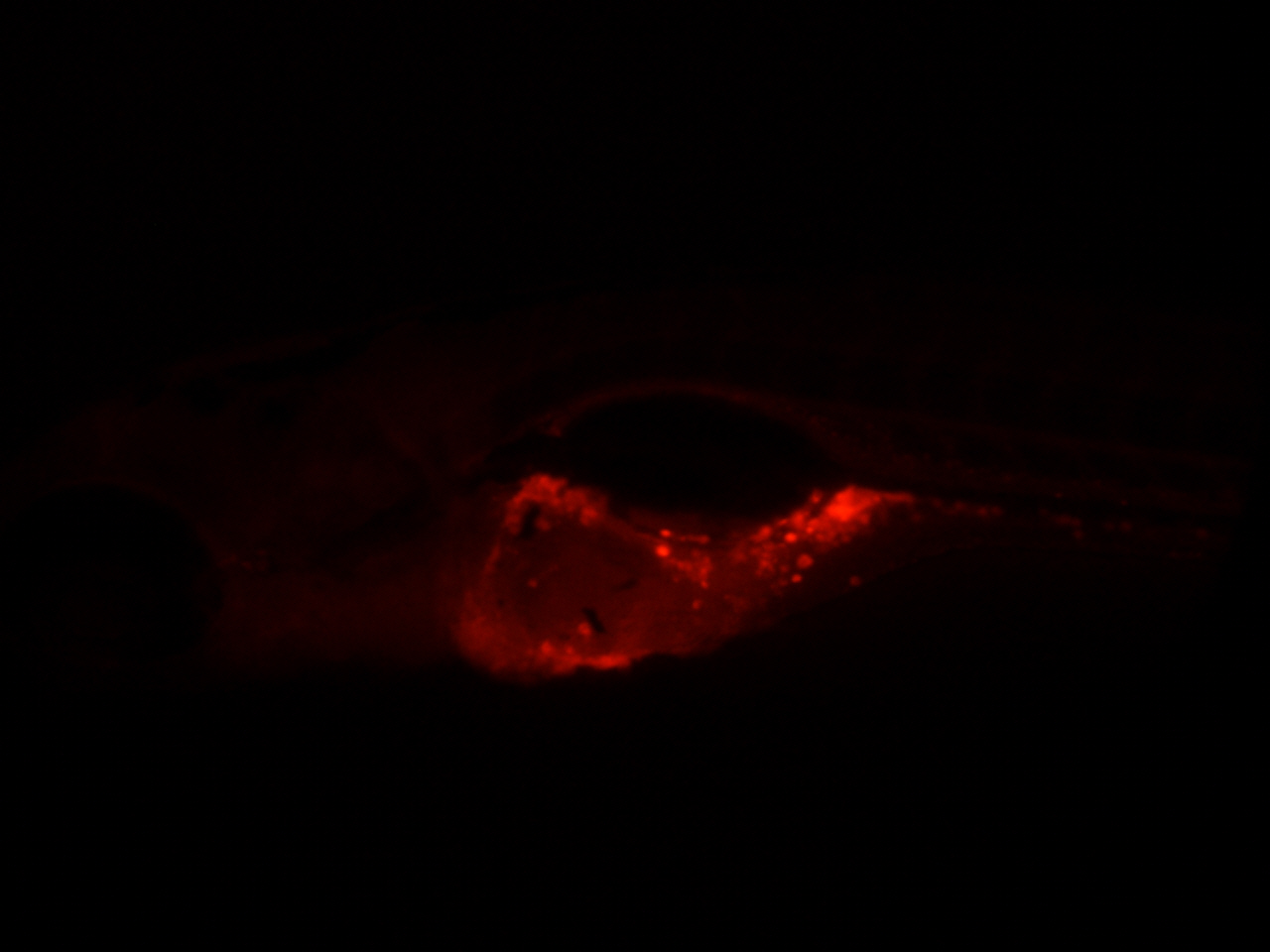

Supplement: Supplementary file 1 [file Data_Sheet_1.ZIP › 250 9/4.jpg]

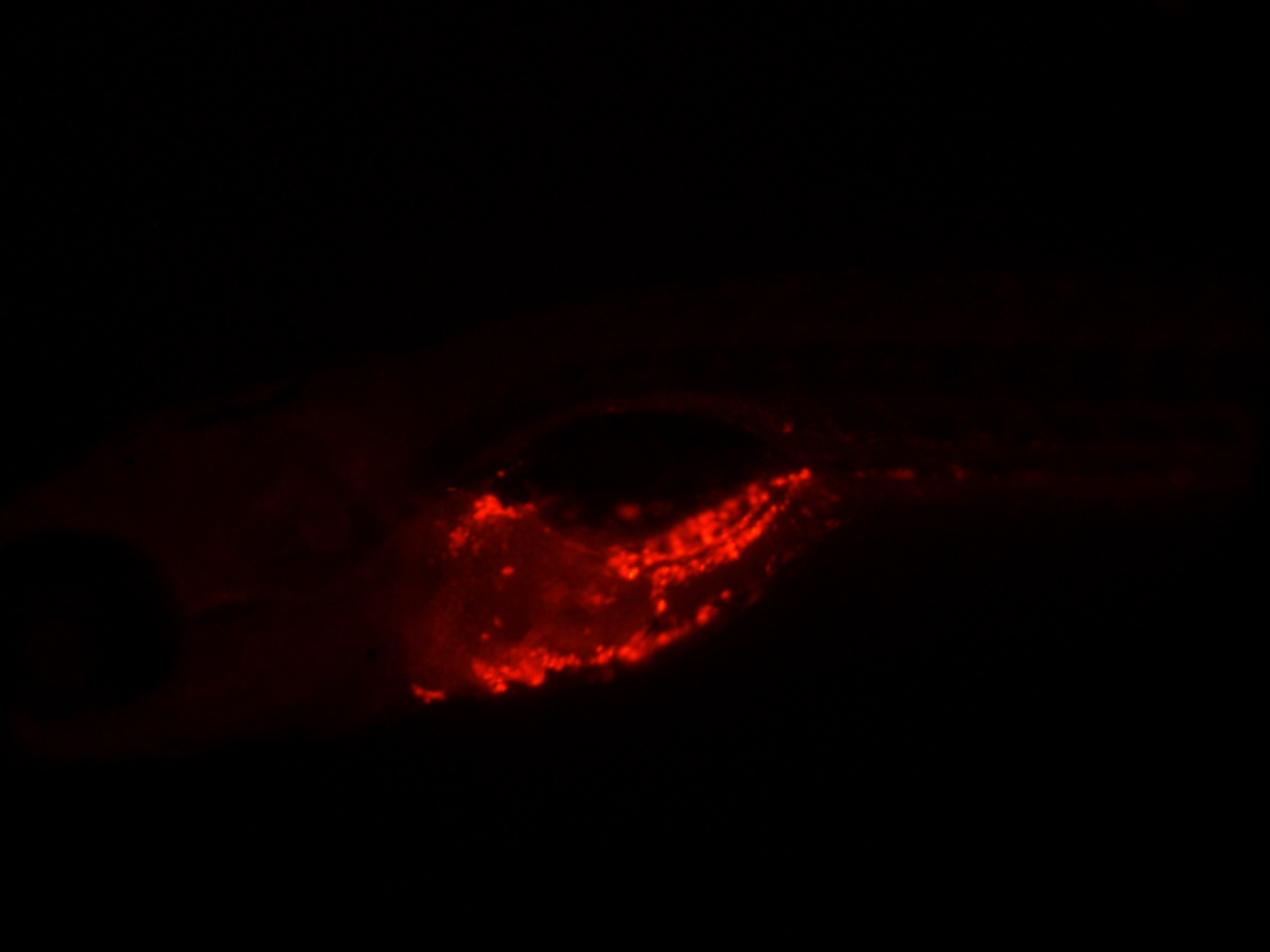

Supplement: Supplementary file 1 [file Data_Sheet_1.ZIP › 250 9/5.jpg]

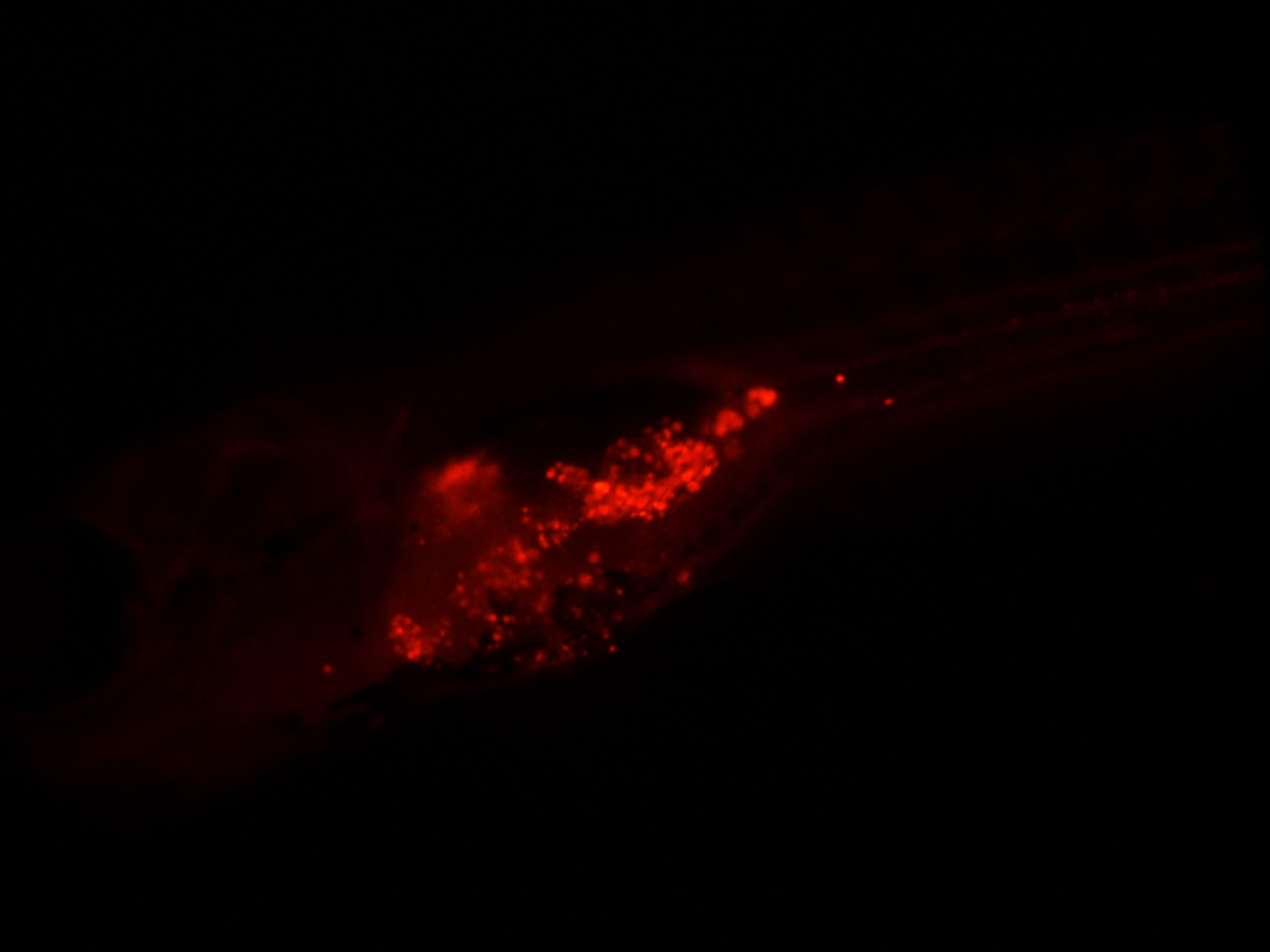

Supplement: Supplementary file 1 [file Data_Sheet_1.ZIP › 250 9/6.jpg]

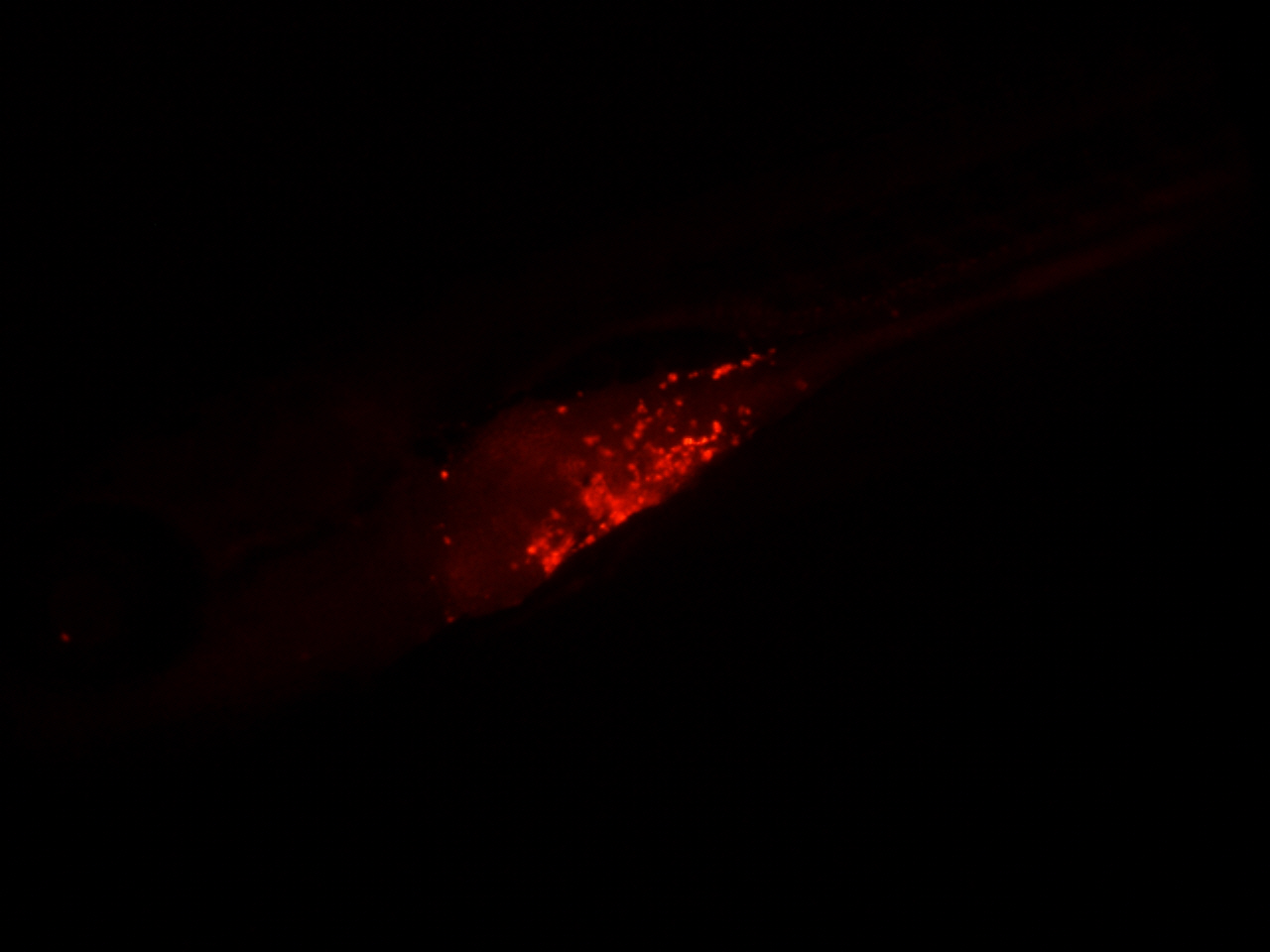

Supplement: Supplementary file 1 [file Data_Sheet_1.ZIP › 250 9/7.jpg]

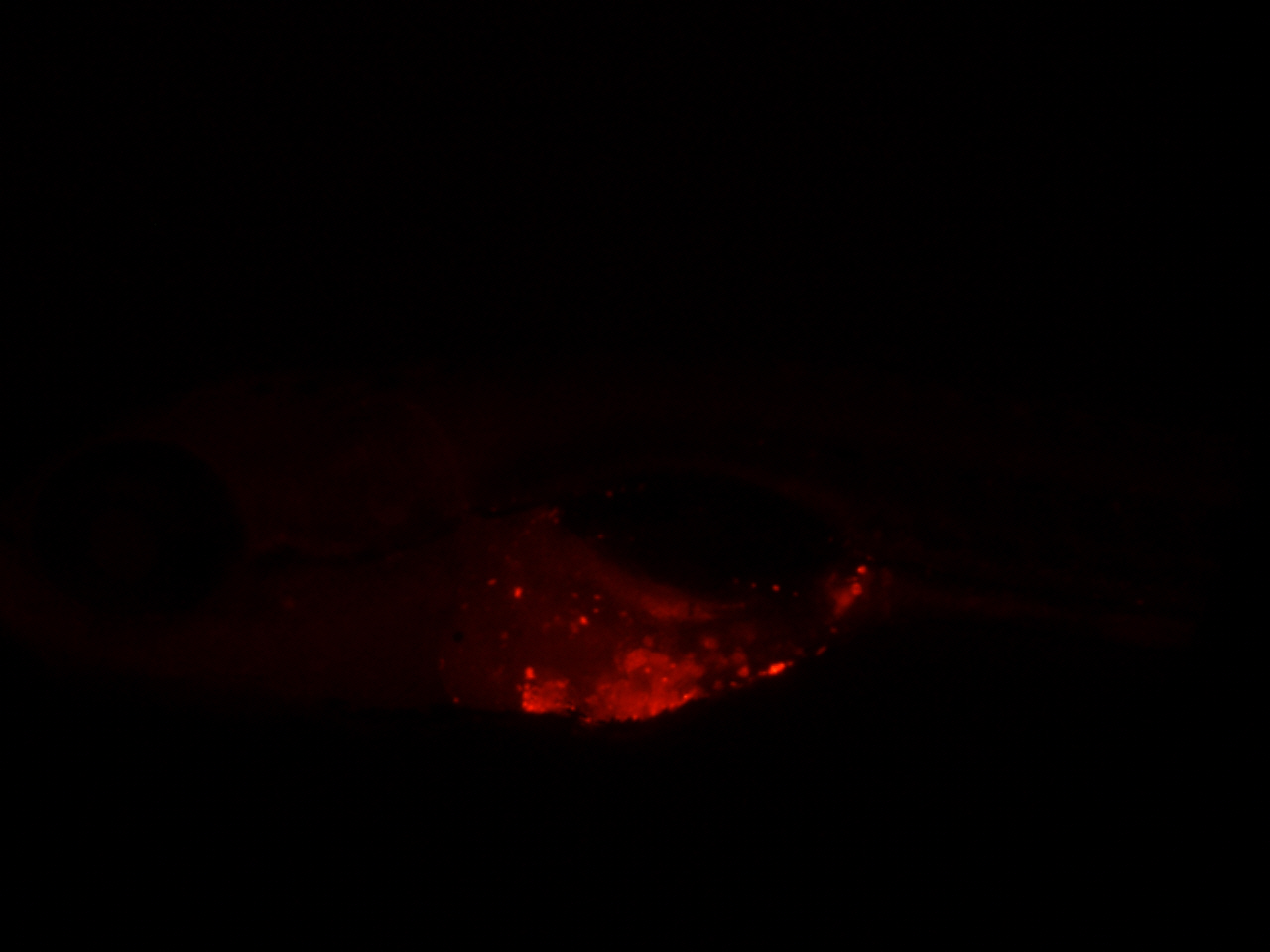

Supplement: Supplementary file 1 [file Data_Sheet_1.ZIP › 250 9/8.jpg]

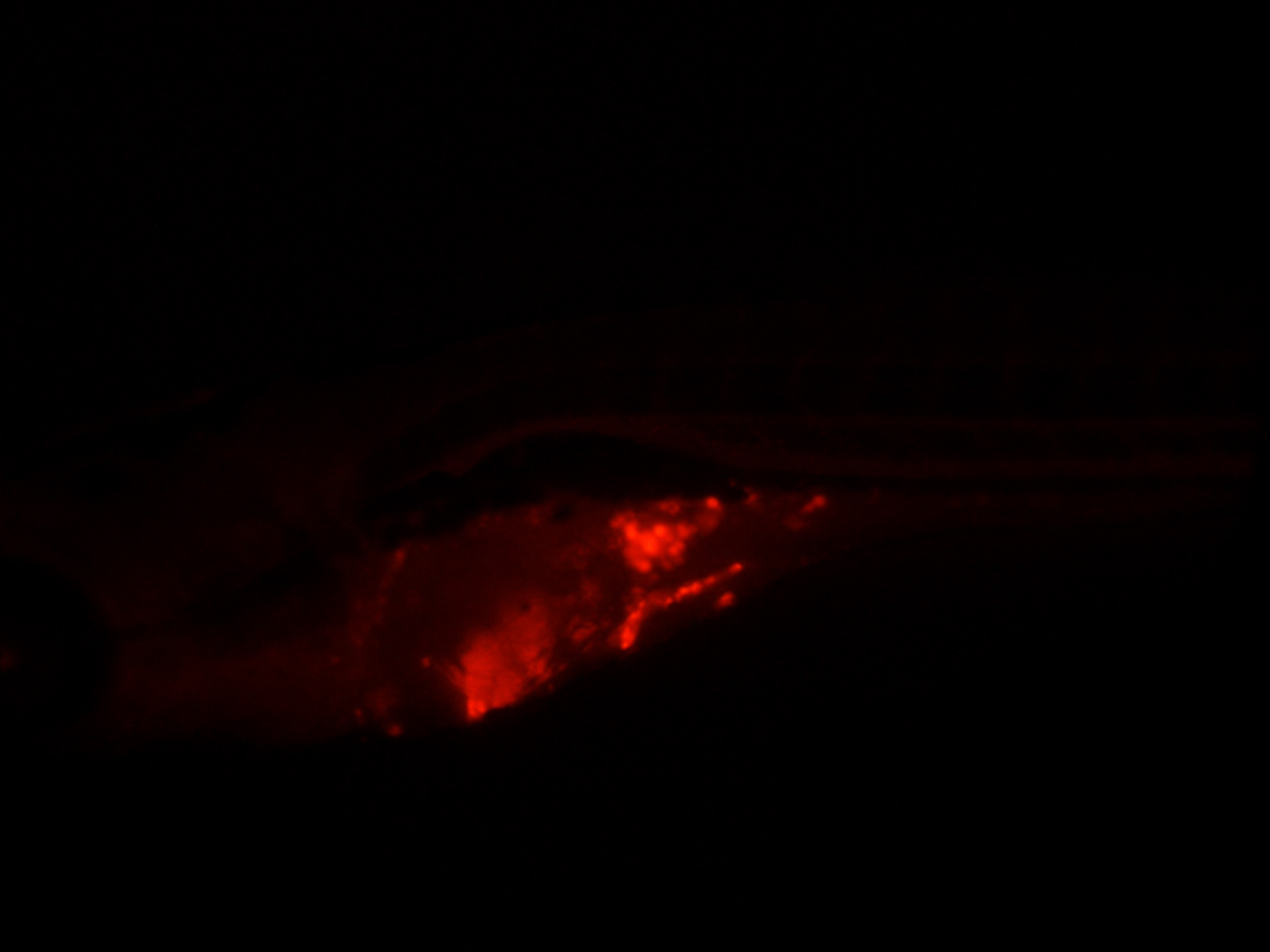

Supplement: Supplementary file 1 [file Data_Sheet_1.ZIP › 250 9/9.jpg]

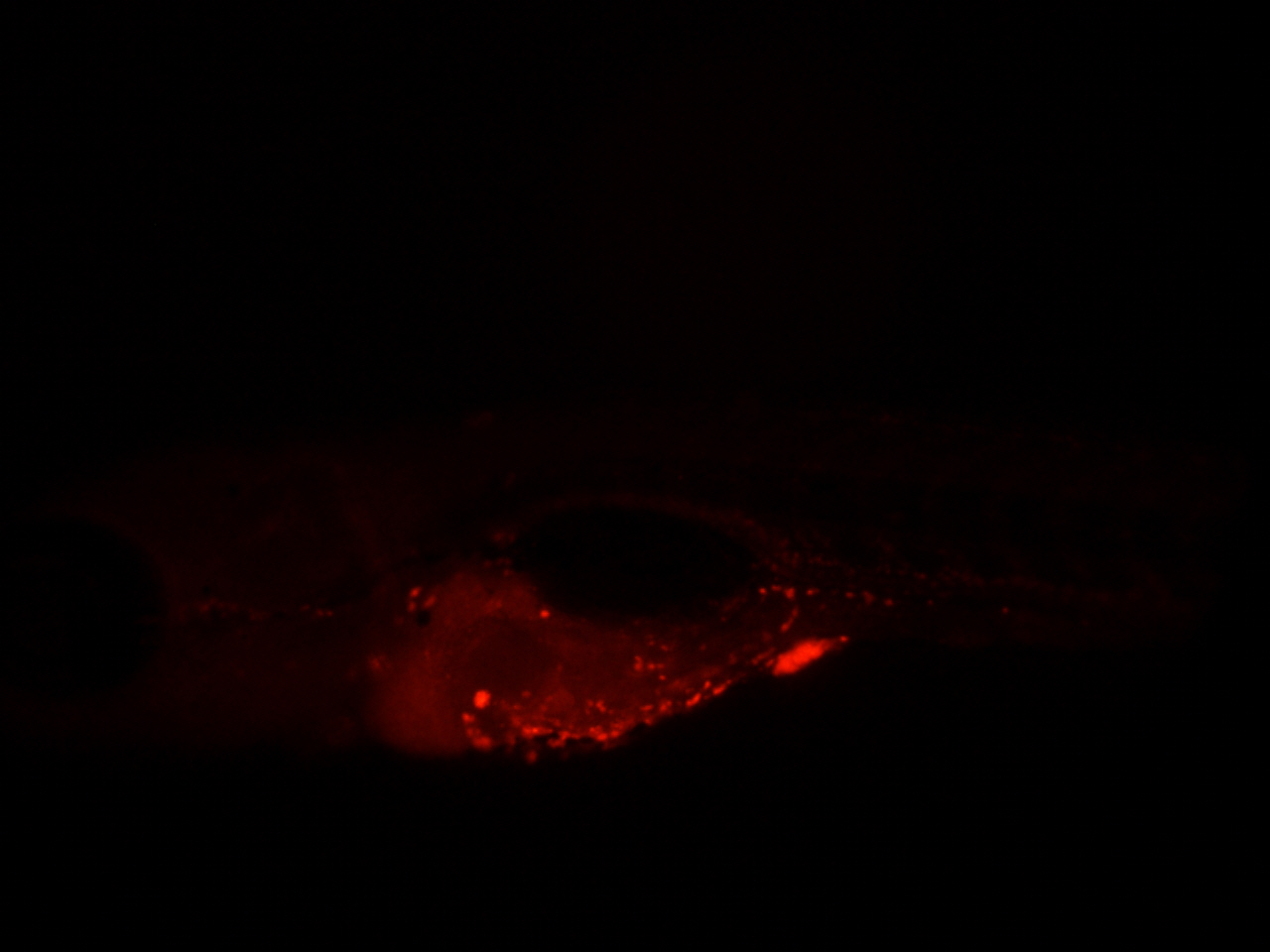

Supplement: Supplementary file 1 [file Data_Sheet_1.ZIP › 250/1.jpg]

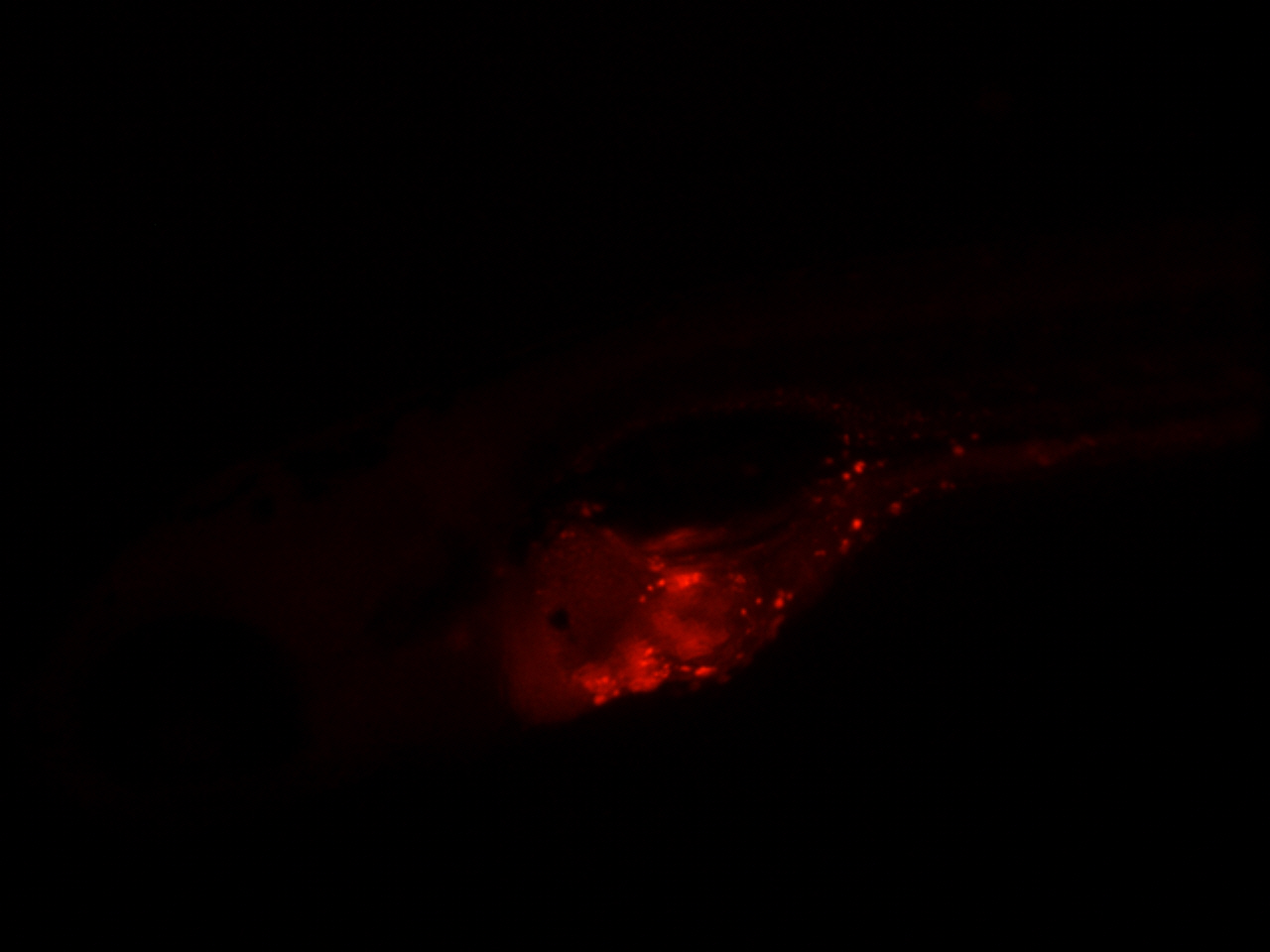

Supplement: Supplementary file 1 [file Data_Sheet_1.ZIP › 250/10.jpg]

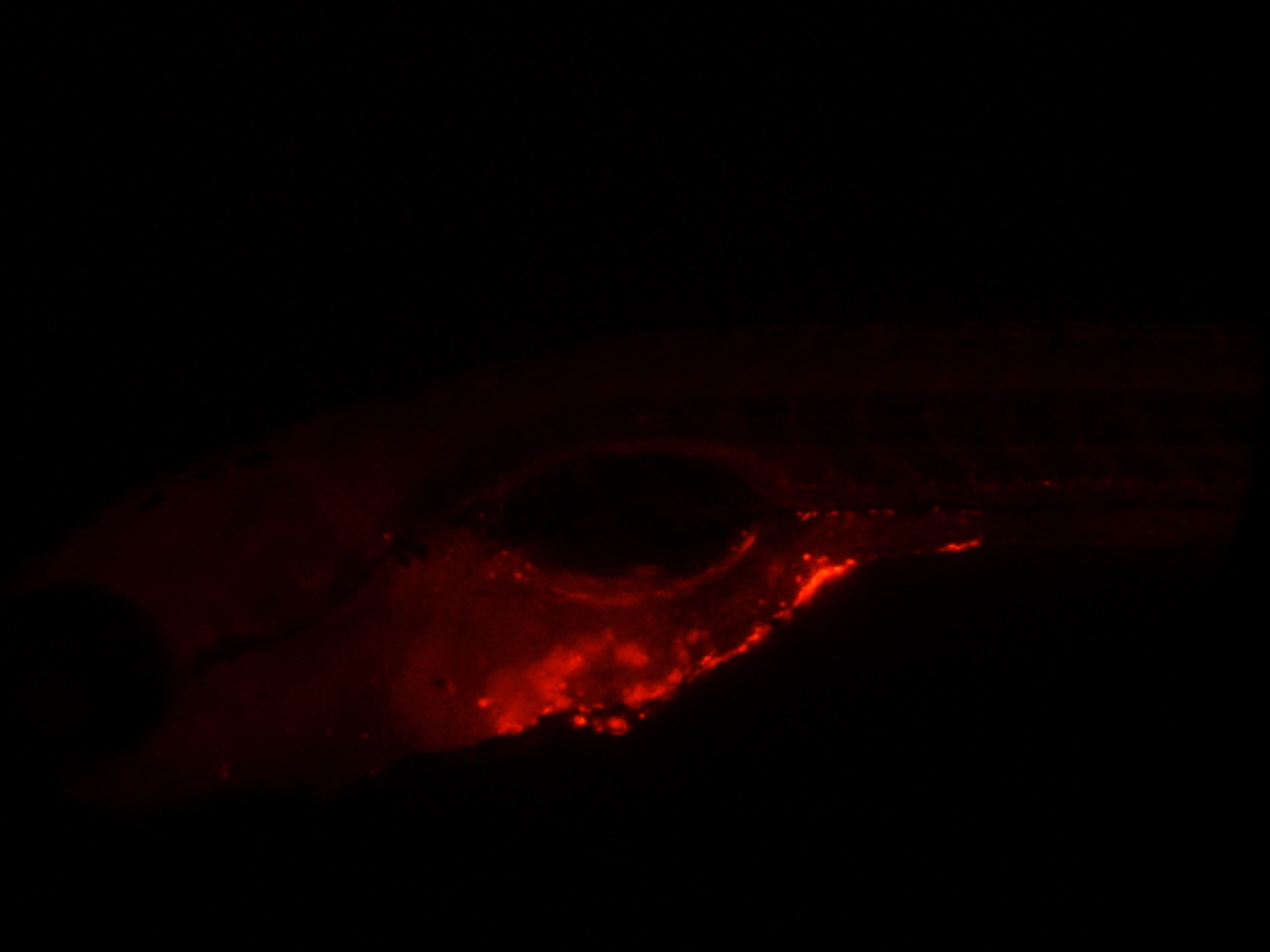

Supplement: Supplementary file 1 [file Data_Sheet_1.ZIP › 250/11.jpg]

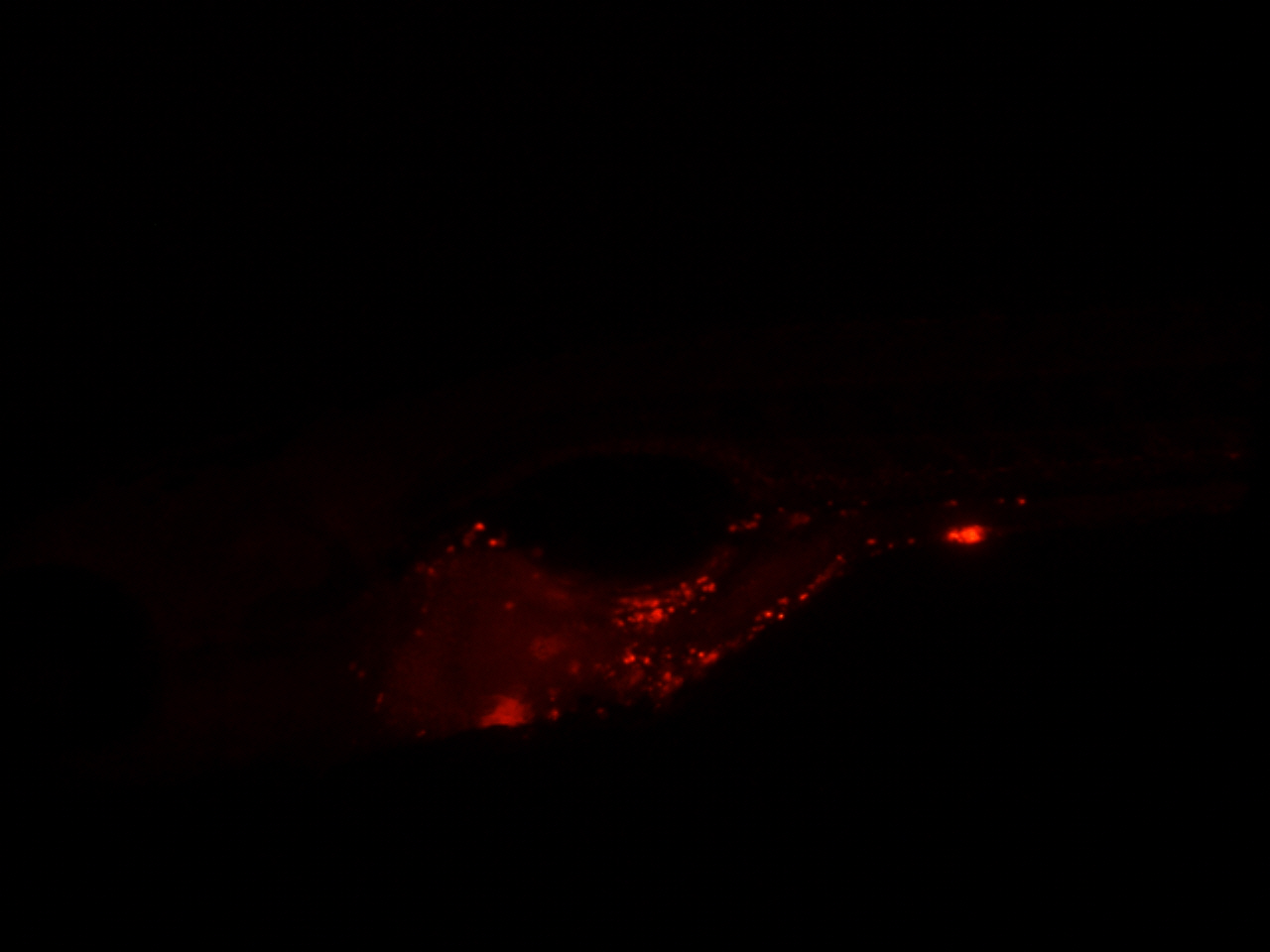

Supplement: Supplementary file 1 [file Data_Sheet_1.ZIP › 250/12.jpg]

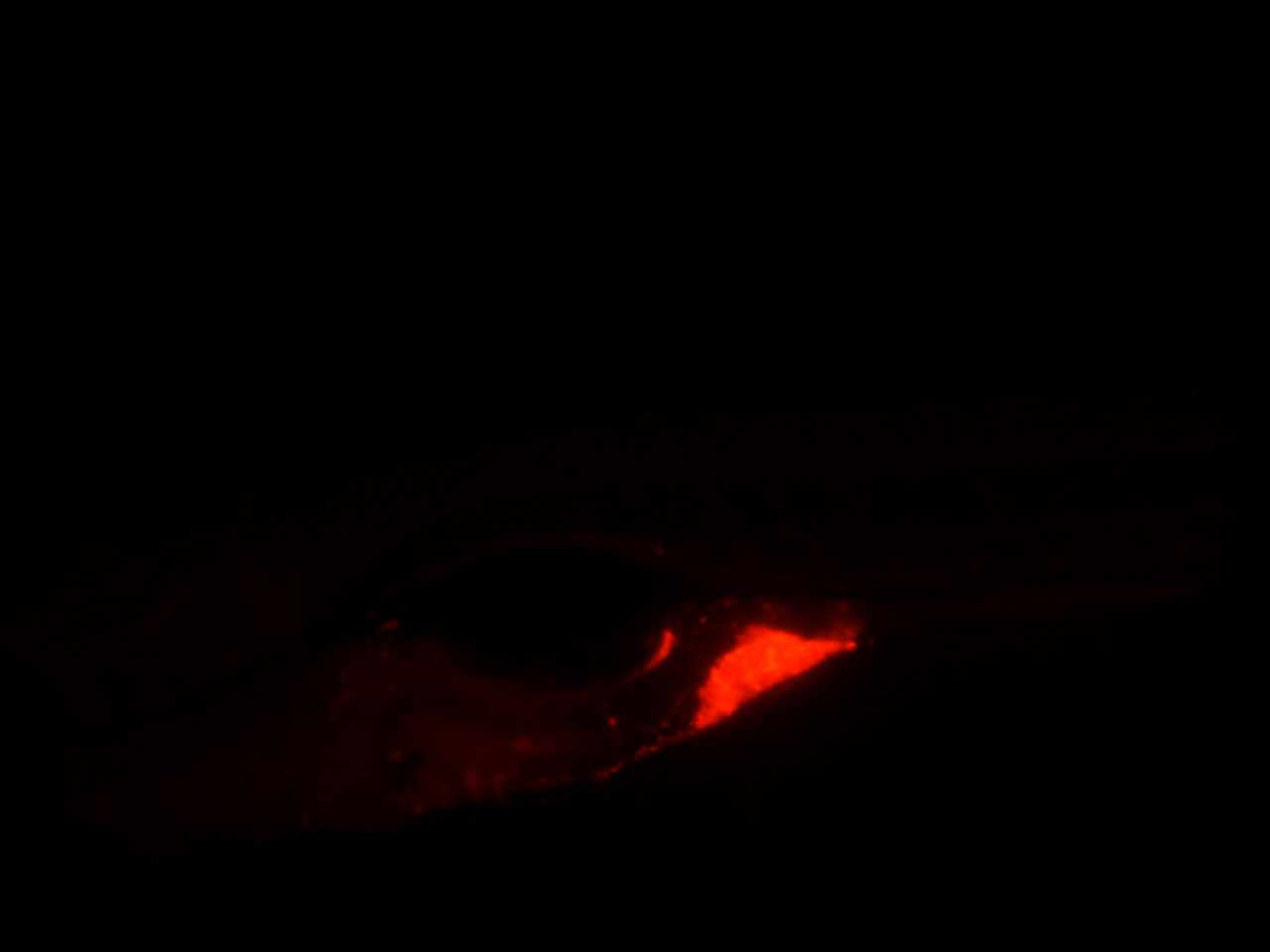

Supplement: Supplementary file 1 [file Data_Sheet_1.ZIP › 250/13.jpg]

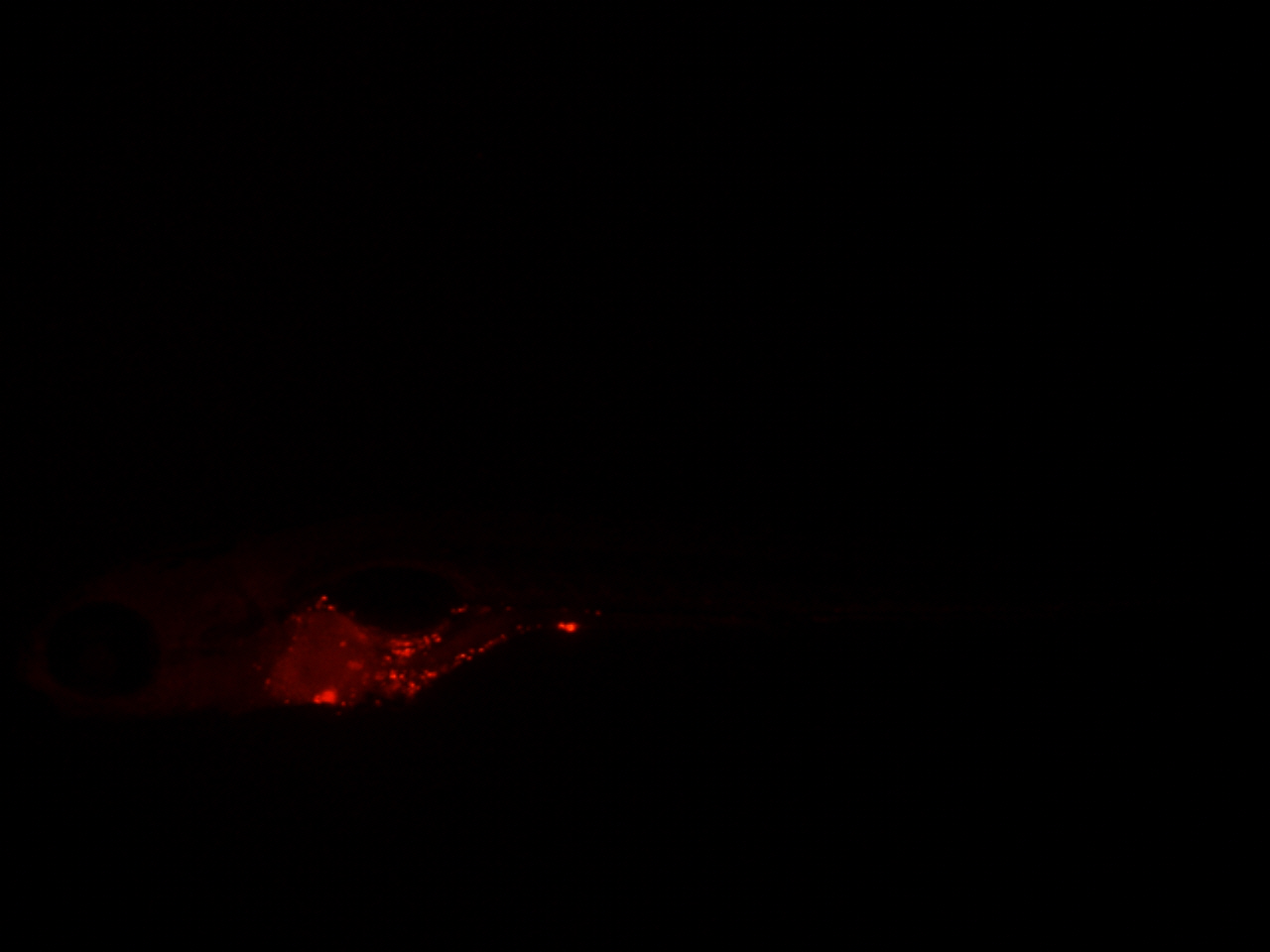

Supplement: Supplementary file 1 [file Data_Sheet_1.ZIP › 250/14-1.jpg]

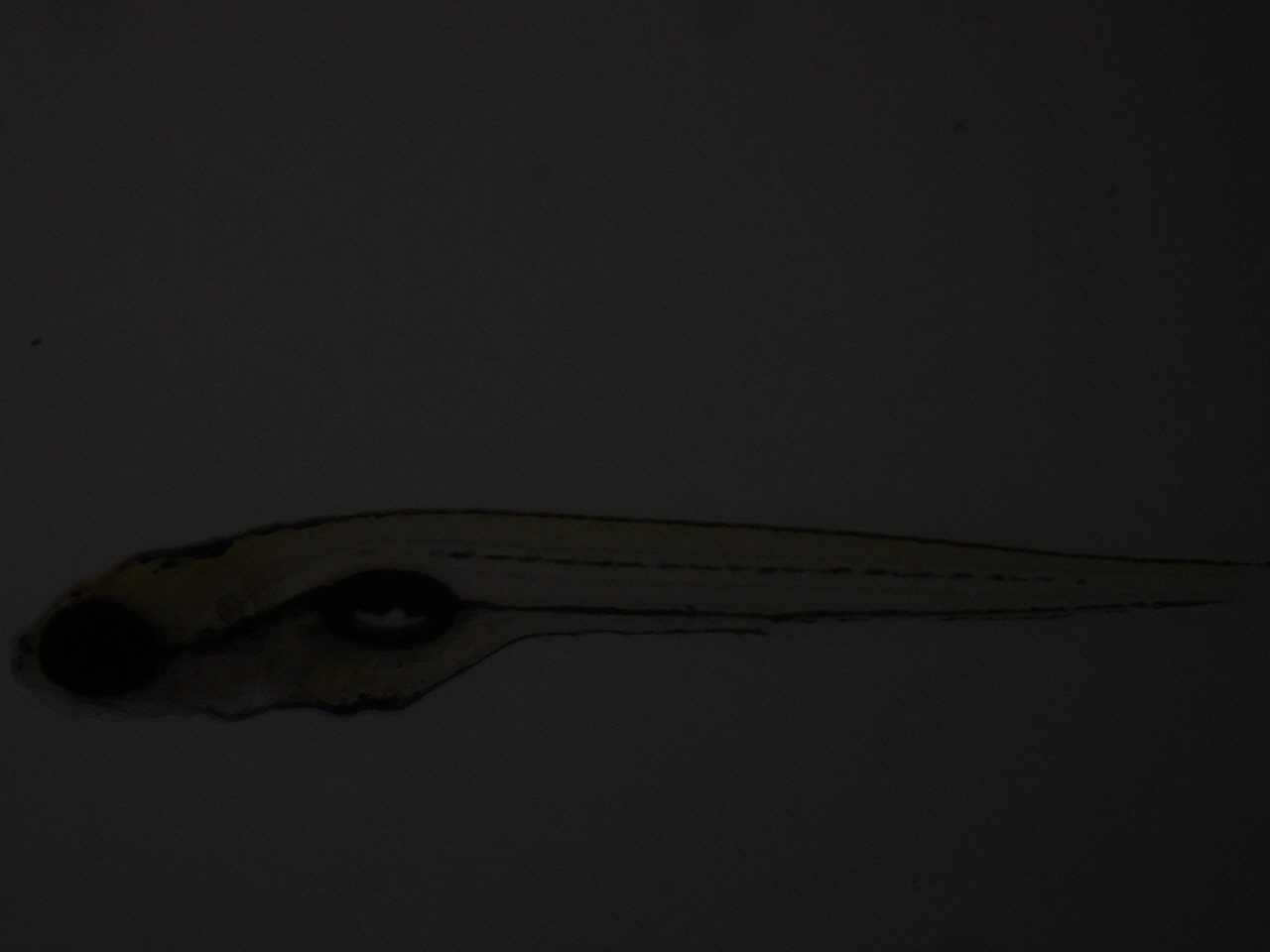

Supplement: Supplementary file 1 [file Data_Sheet_1.ZIP › 250/14-2.jpg]

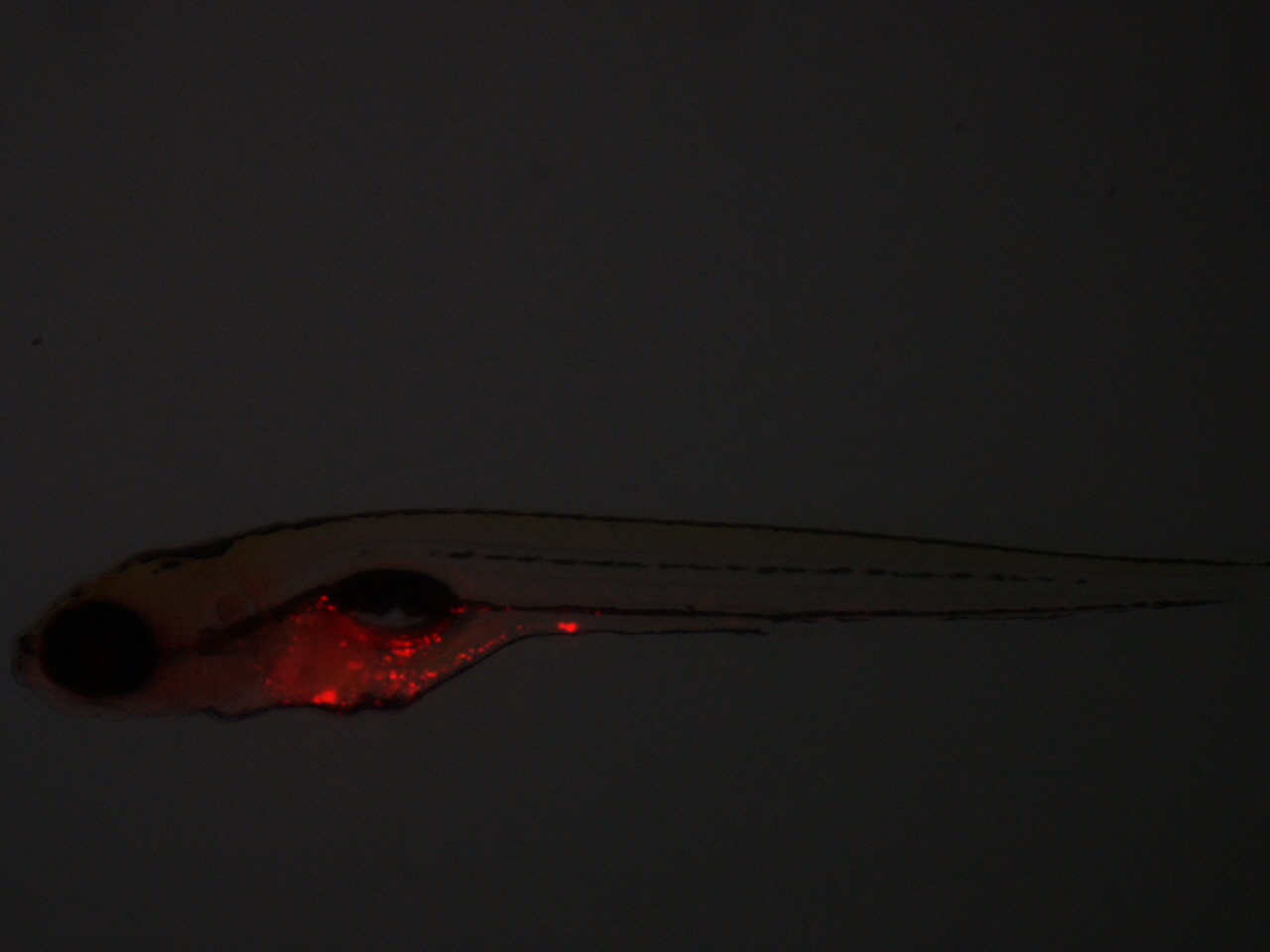

Supplement: Supplementary file 1 [file Data_Sheet_1.ZIP › 250/14-3.jpg]

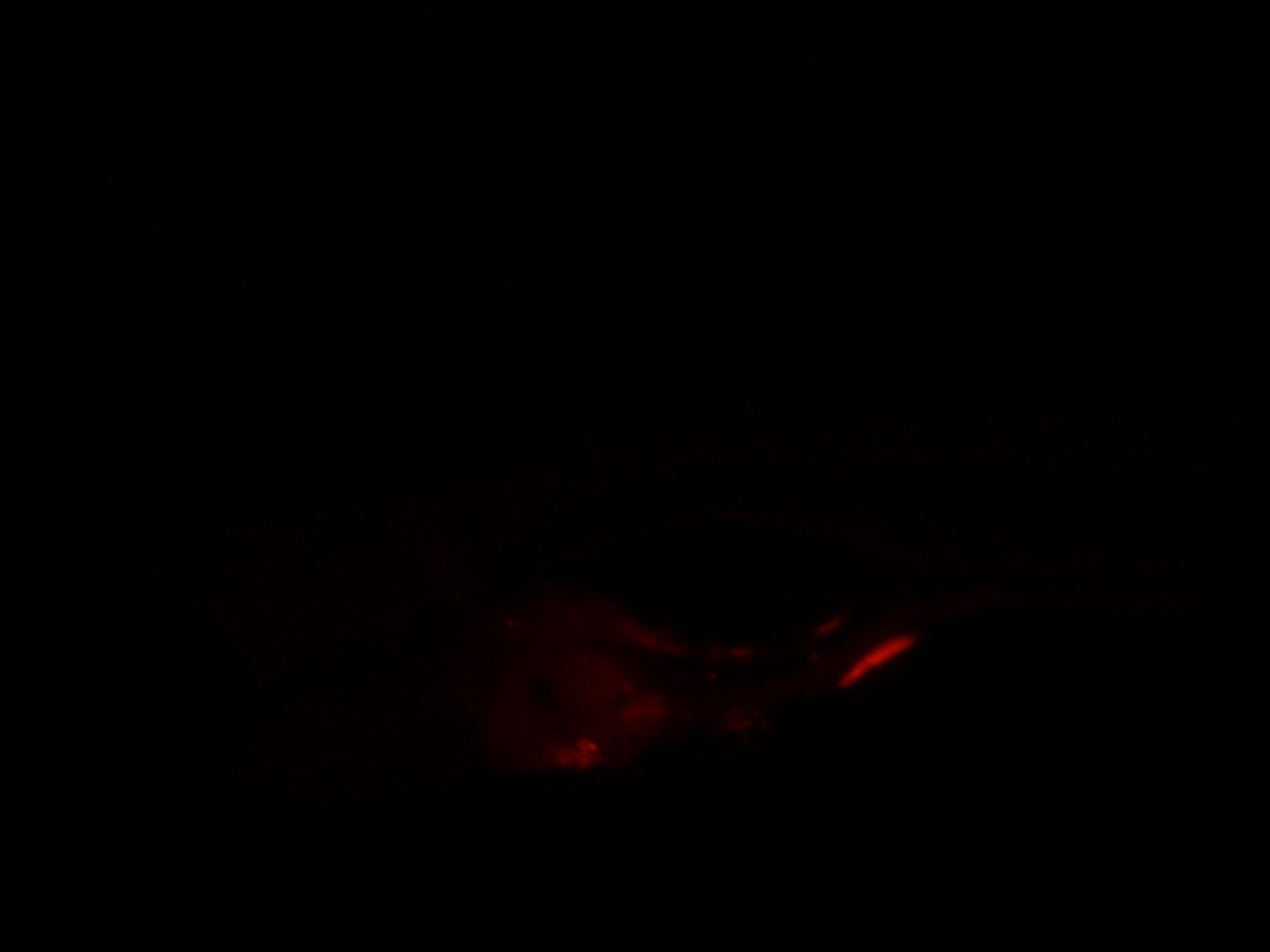

Supplement: Supplementary file 1 [file Data_Sheet_1.ZIP › 250/14.jpg]

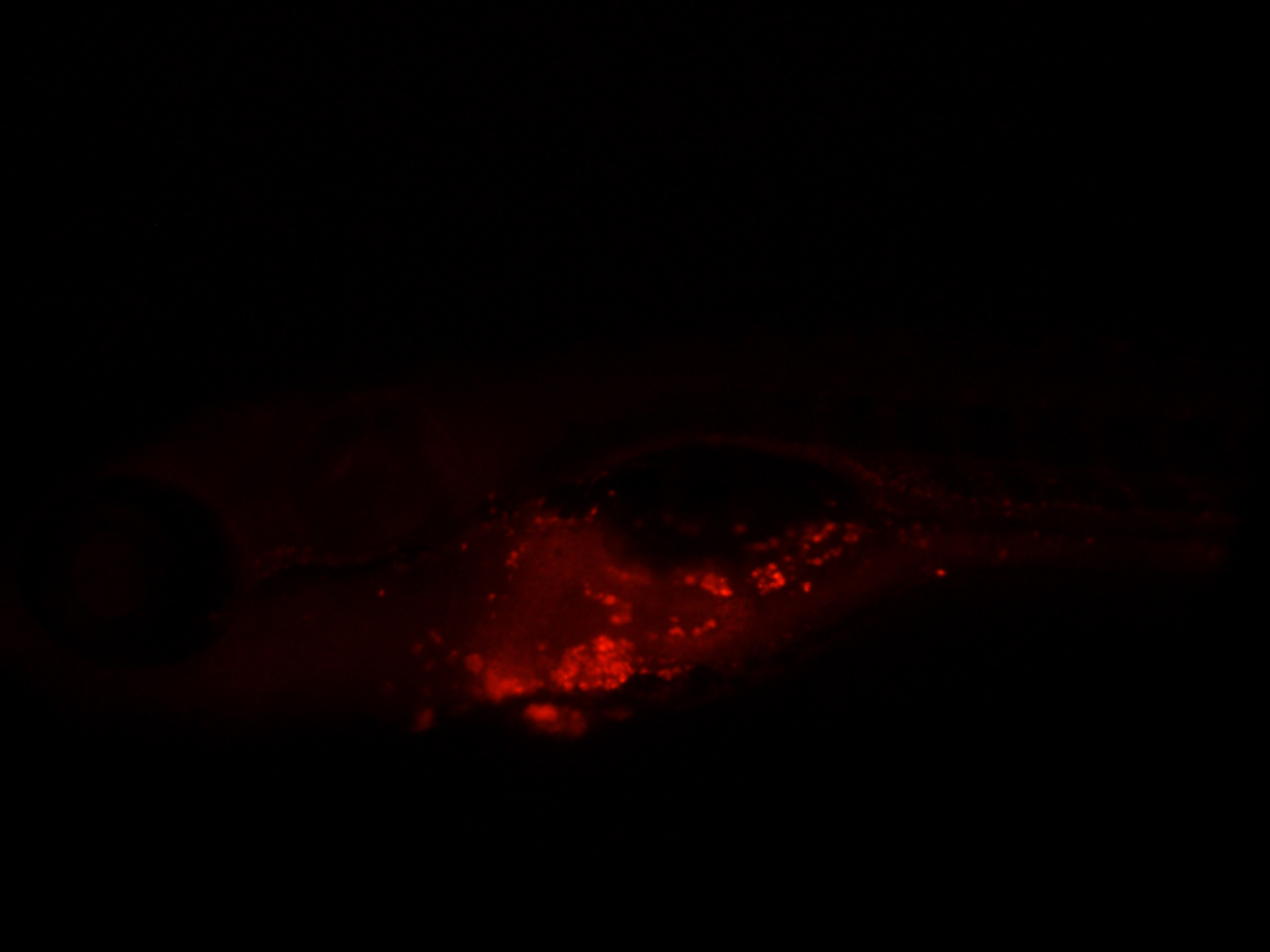

Supplement: Supplementary file 1 [file Data_Sheet_1.ZIP › 250/2.jpg]

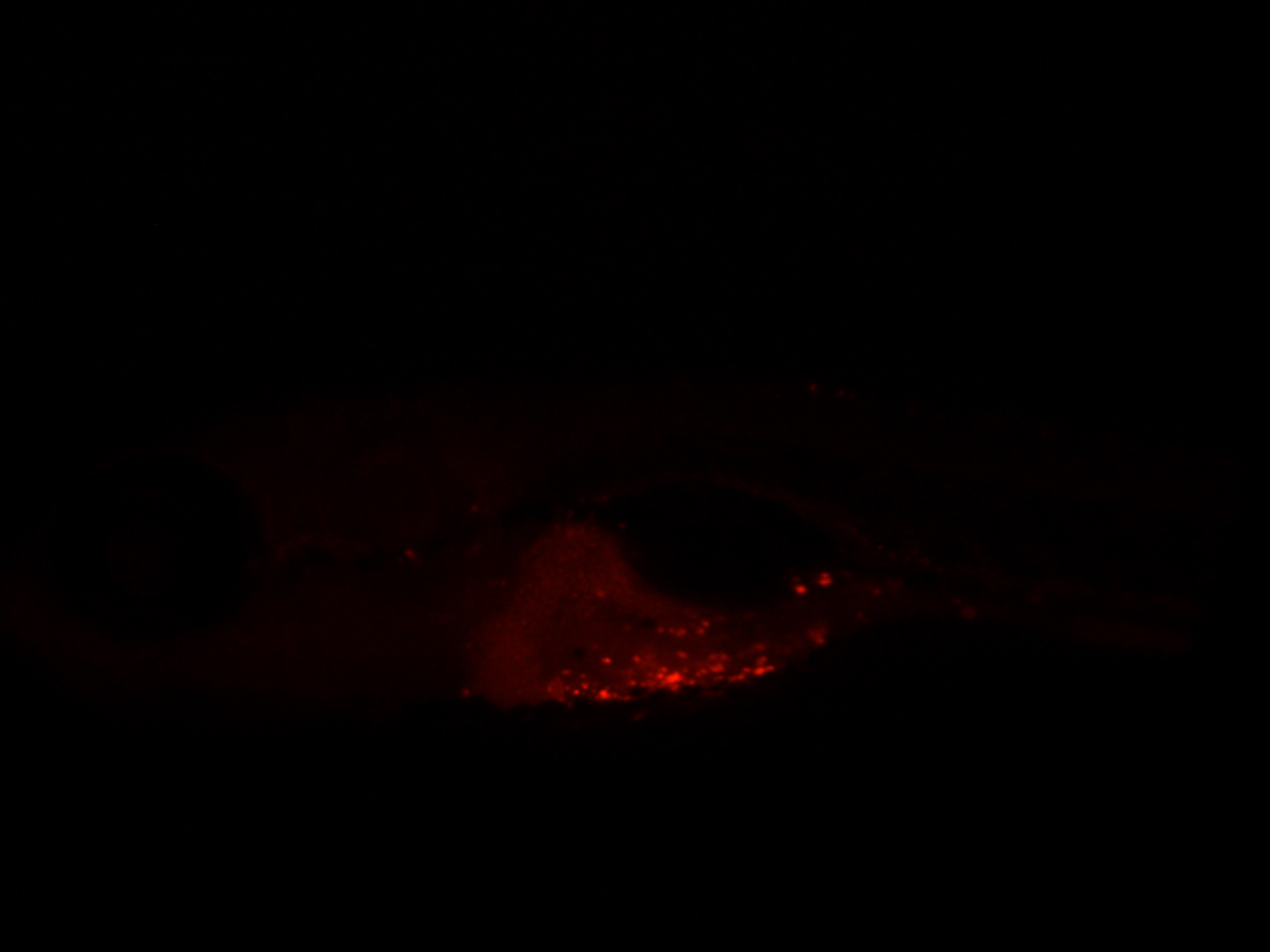

Supplement: Supplementary file 1 [file Data_Sheet_1.ZIP › 250/3.jpg]

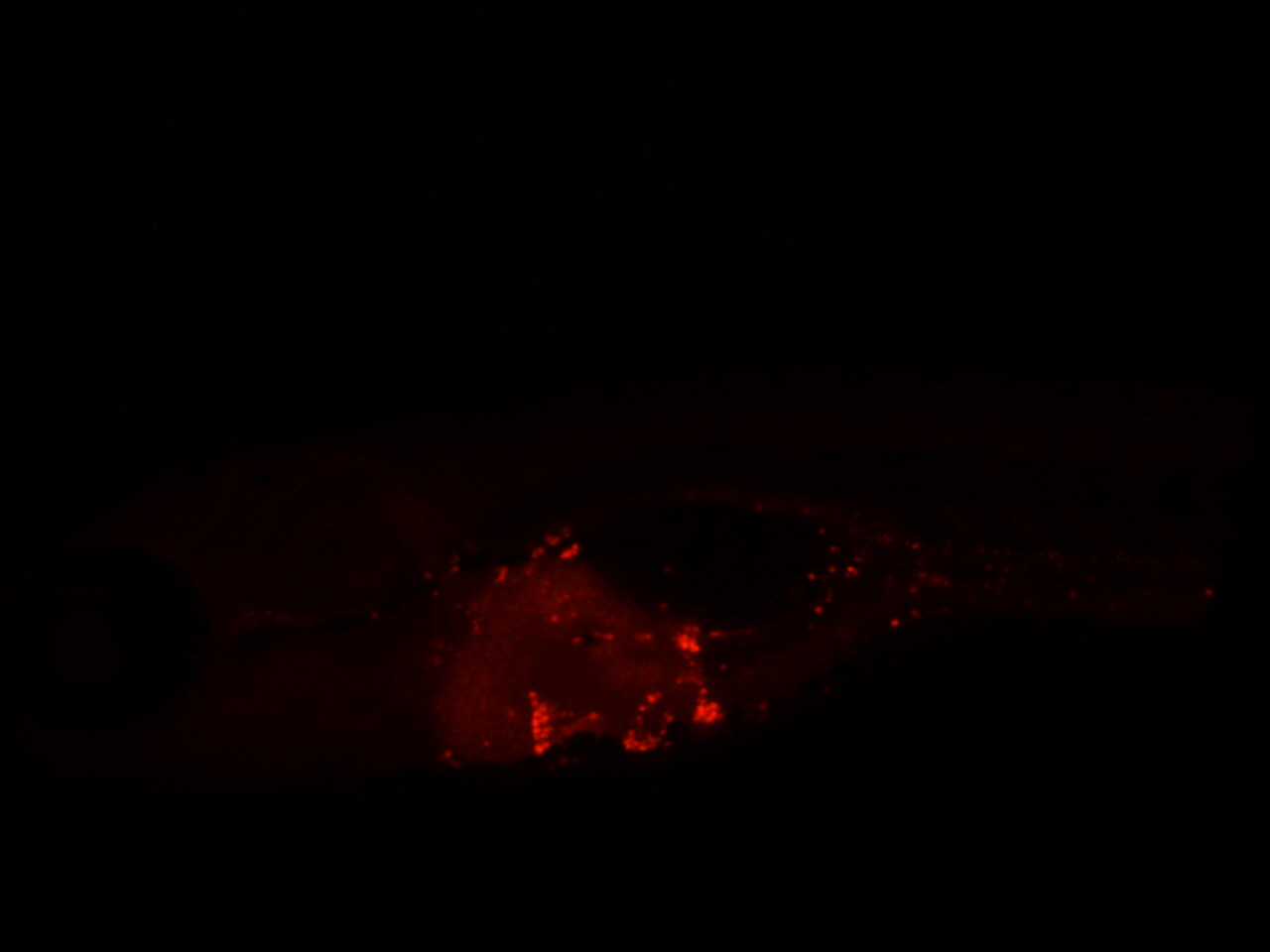

Supplement: Supplementary file 1 [file Data_Sheet_1.ZIP › 250/4.jpg]

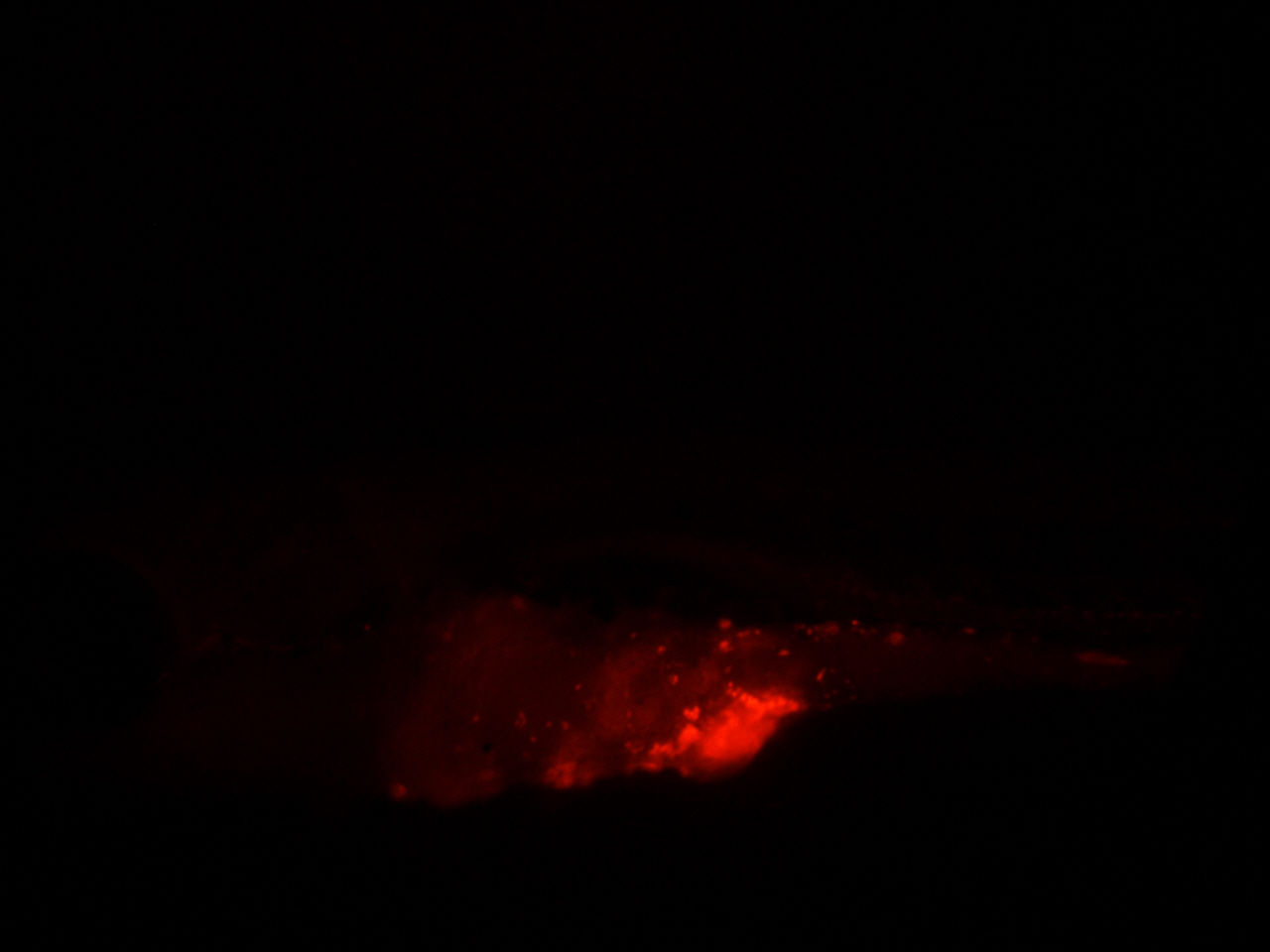

Supplement: Supplementary file 1 [file Data_Sheet_1.ZIP › 250/5.jpg]

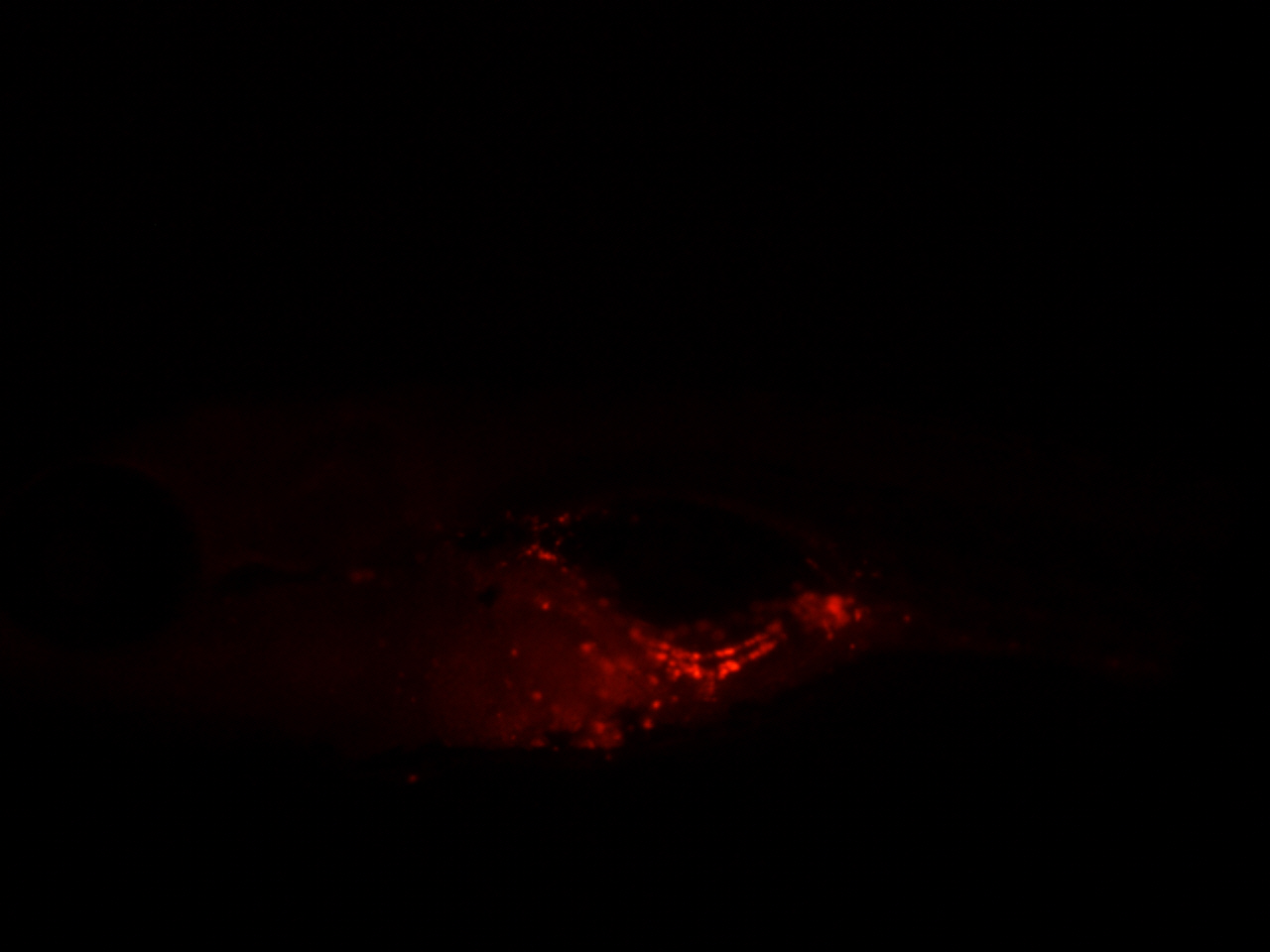

Supplement: Supplementary file 1 [file Data_Sheet_1.ZIP › 250/6.jpg]

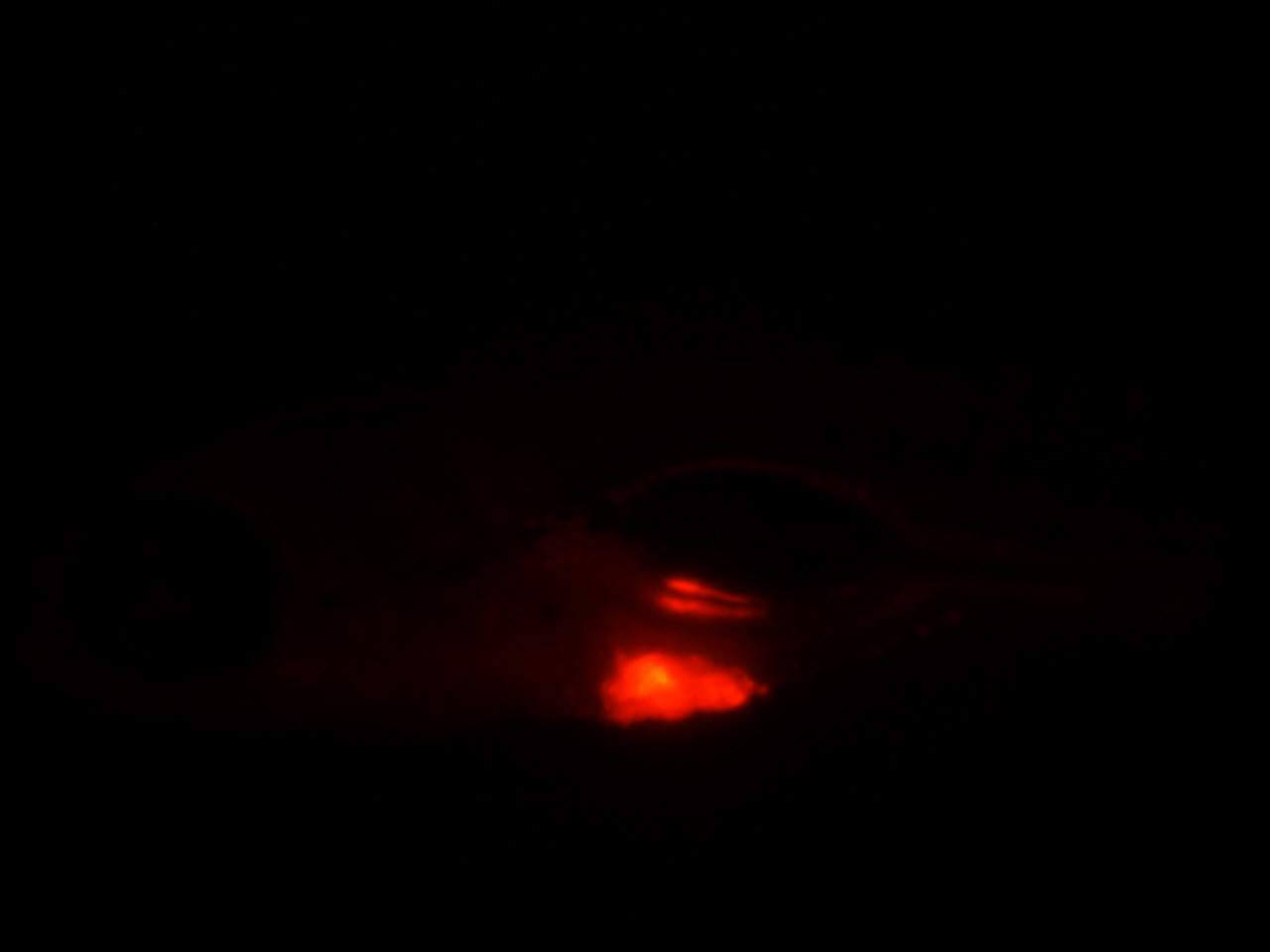

Supplement: Supplementary file 1 [file Data_Sheet_1.ZIP › 250/7.jpg]

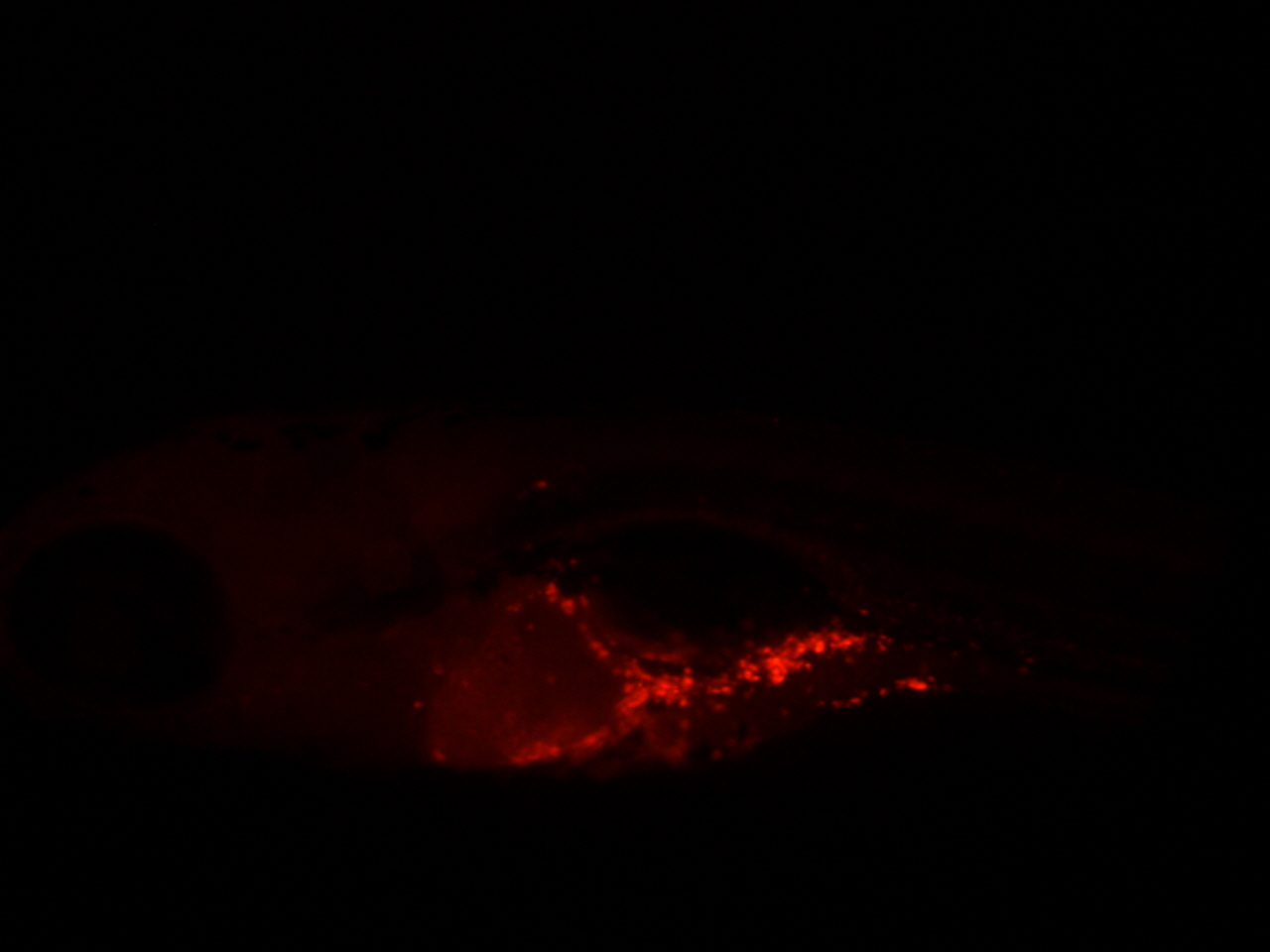

Supplement: Supplementary file 1 [file Data_Sheet_1.ZIP › 250/8.jpg]

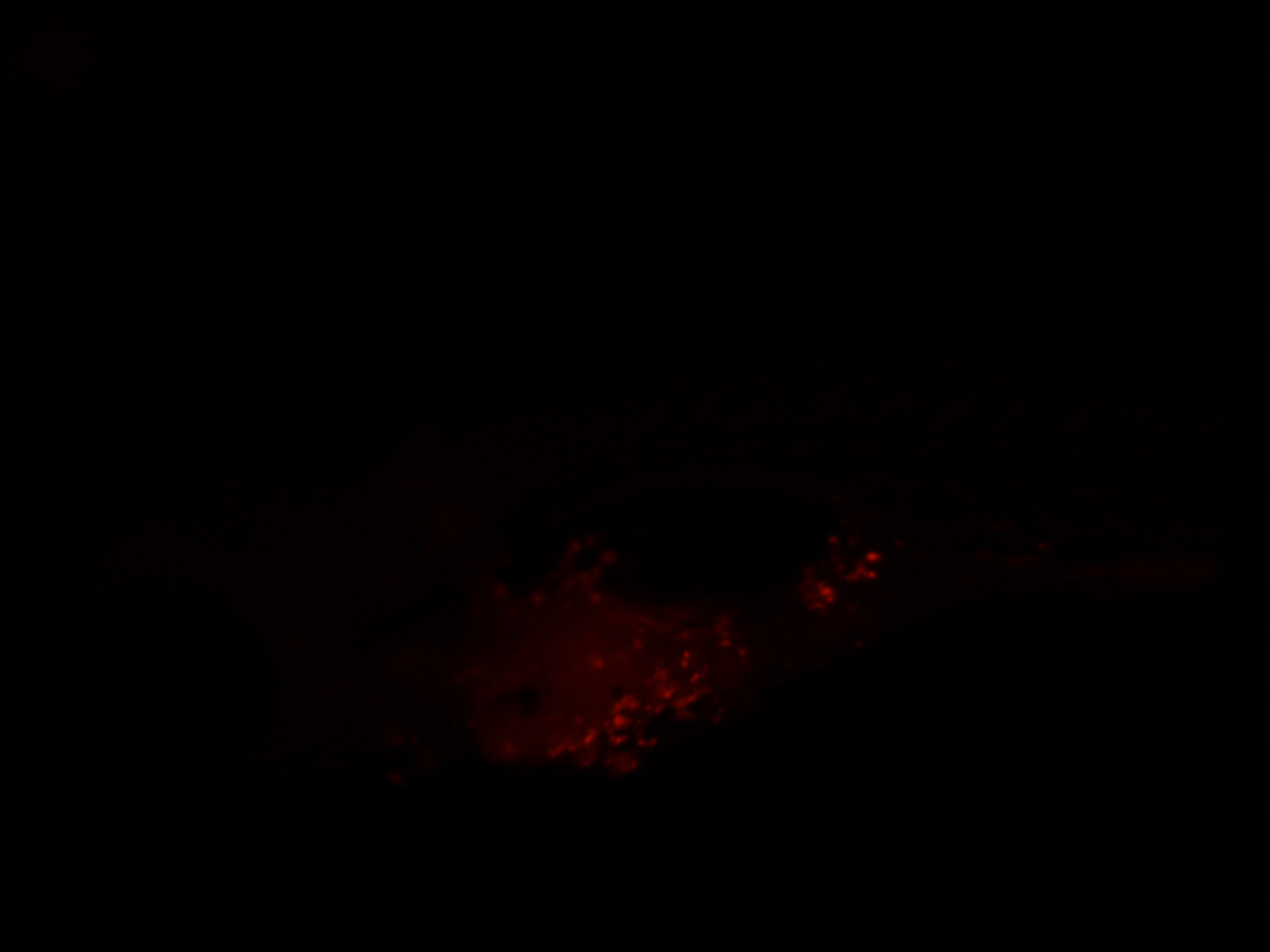

Supplement: Supplementary file 1 [file Data_Sheet_1.ZIP › 250/9.jpg]

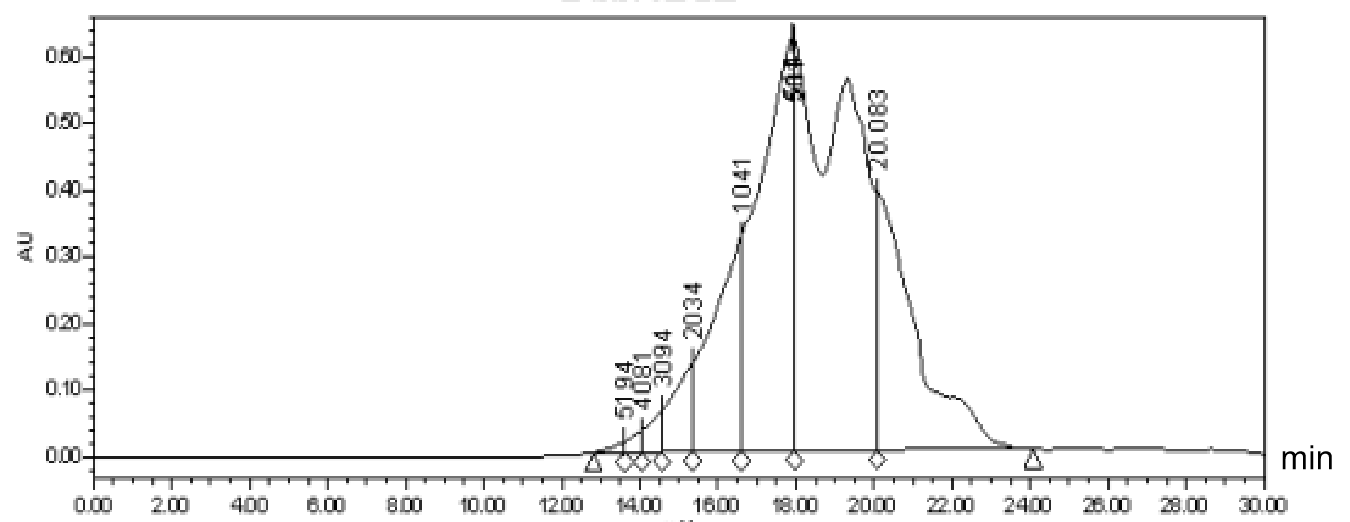

Supplement: Supplementary file 1 [file Data_Sheet_1.ZIP › HPLC.tif]

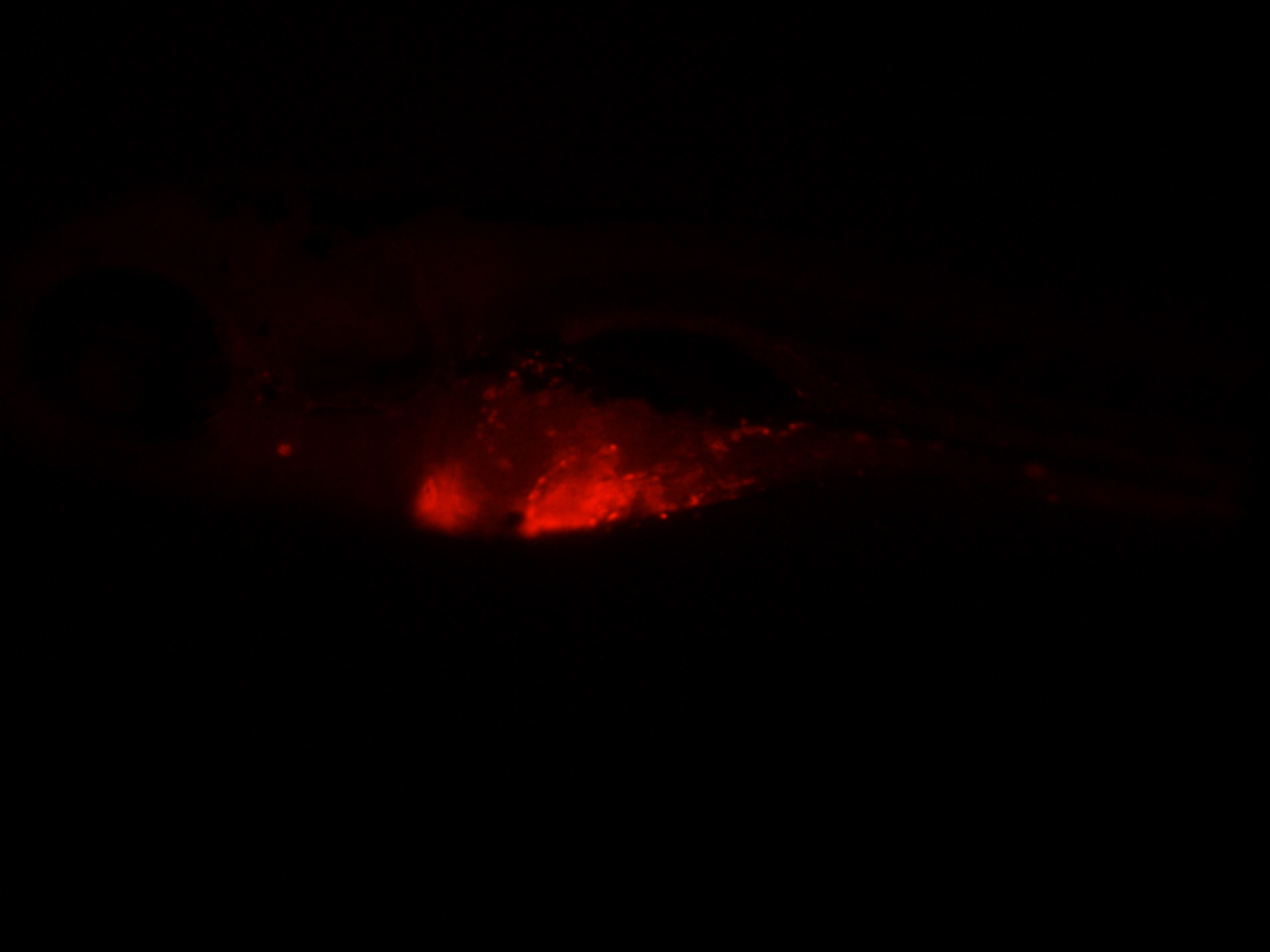

Supplement: Supplementary file 1 [file Data_Sheet_1.ZIP › model/1.jpg]

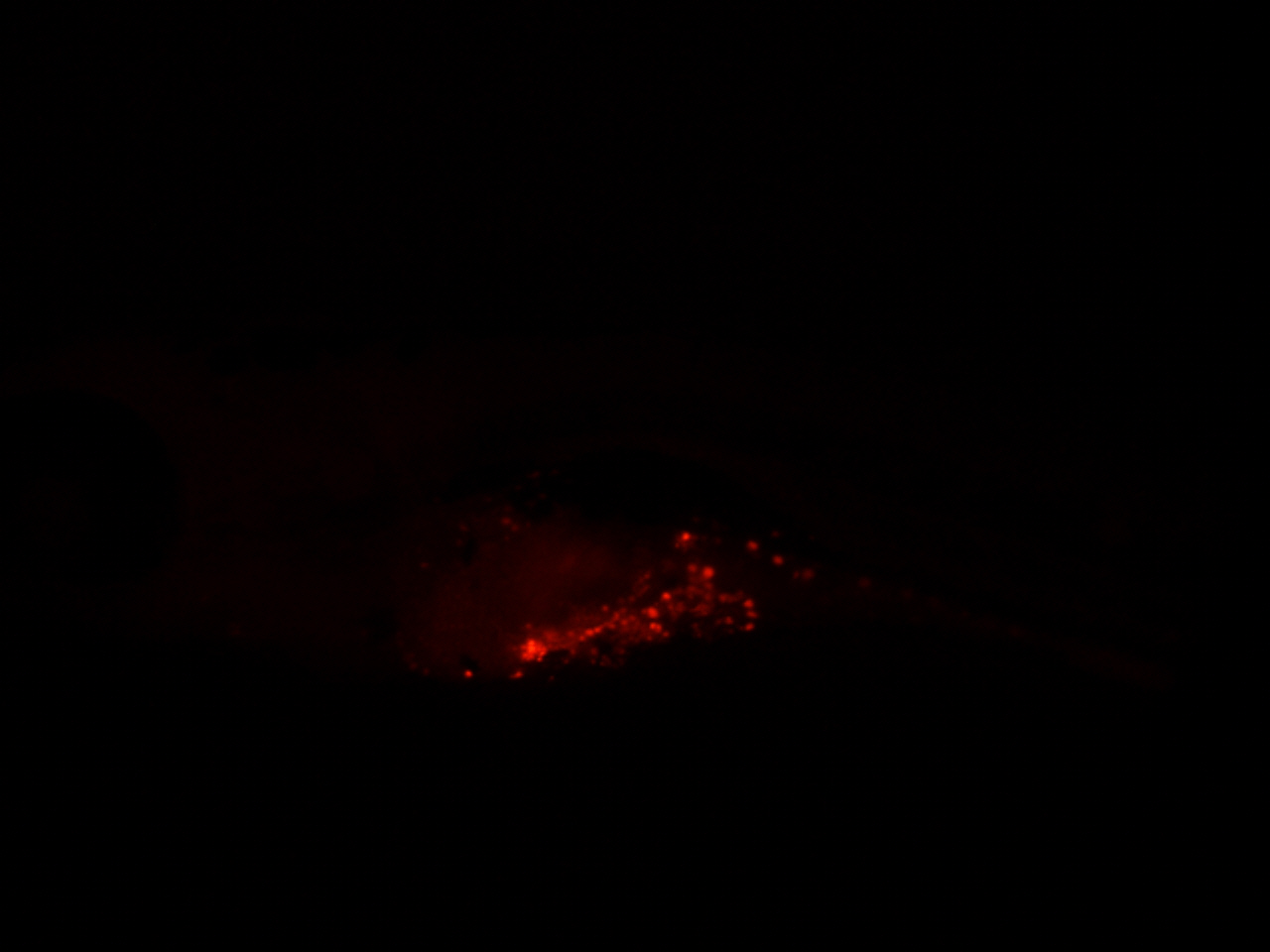

Supplement: Supplementary file 1 [file Data_Sheet_1.ZIP › model/10.jpg]

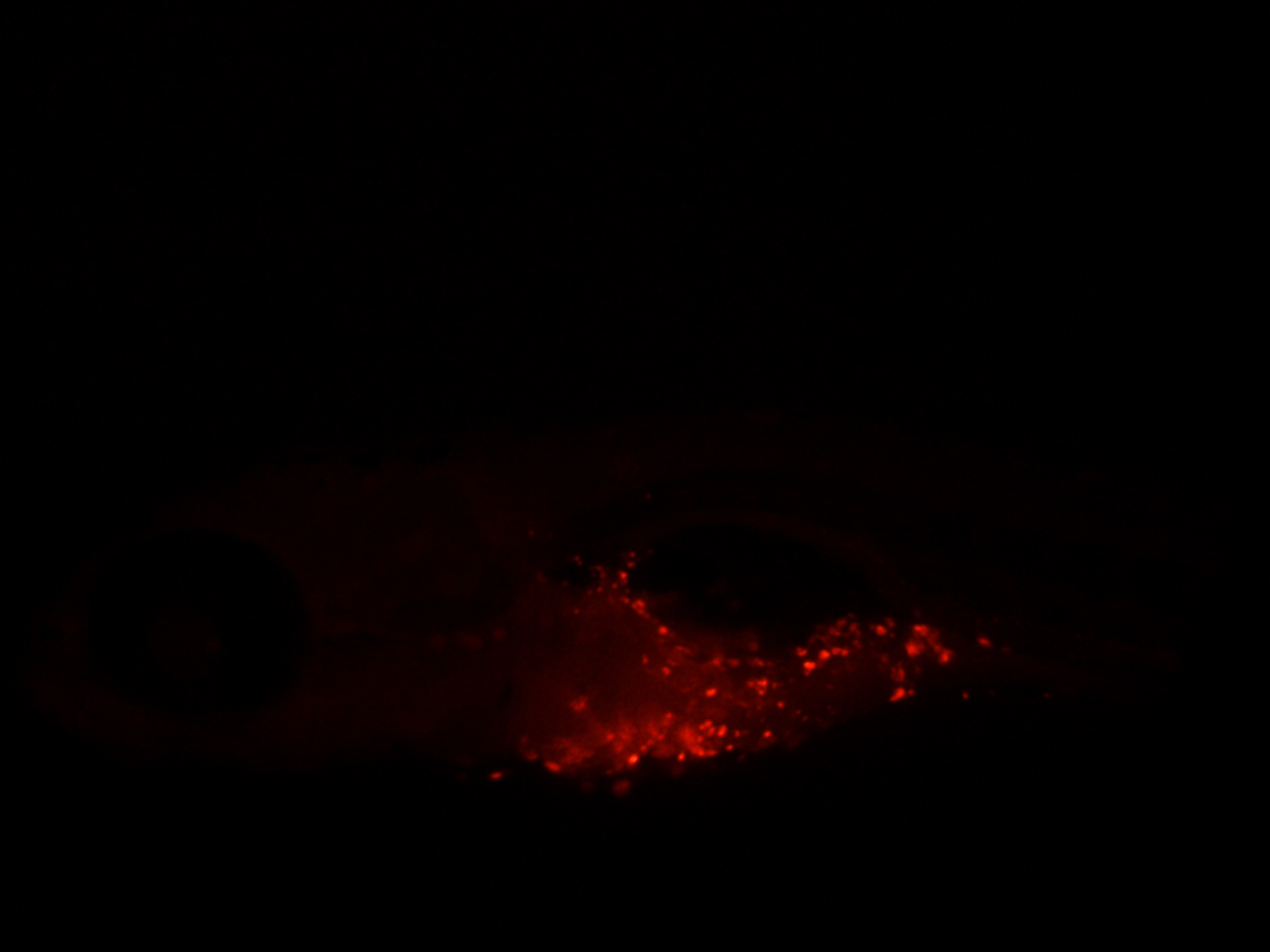

Supplement: Supplementary file 1 [file Data_Sheet_1.ZIP › model/11.jpg]

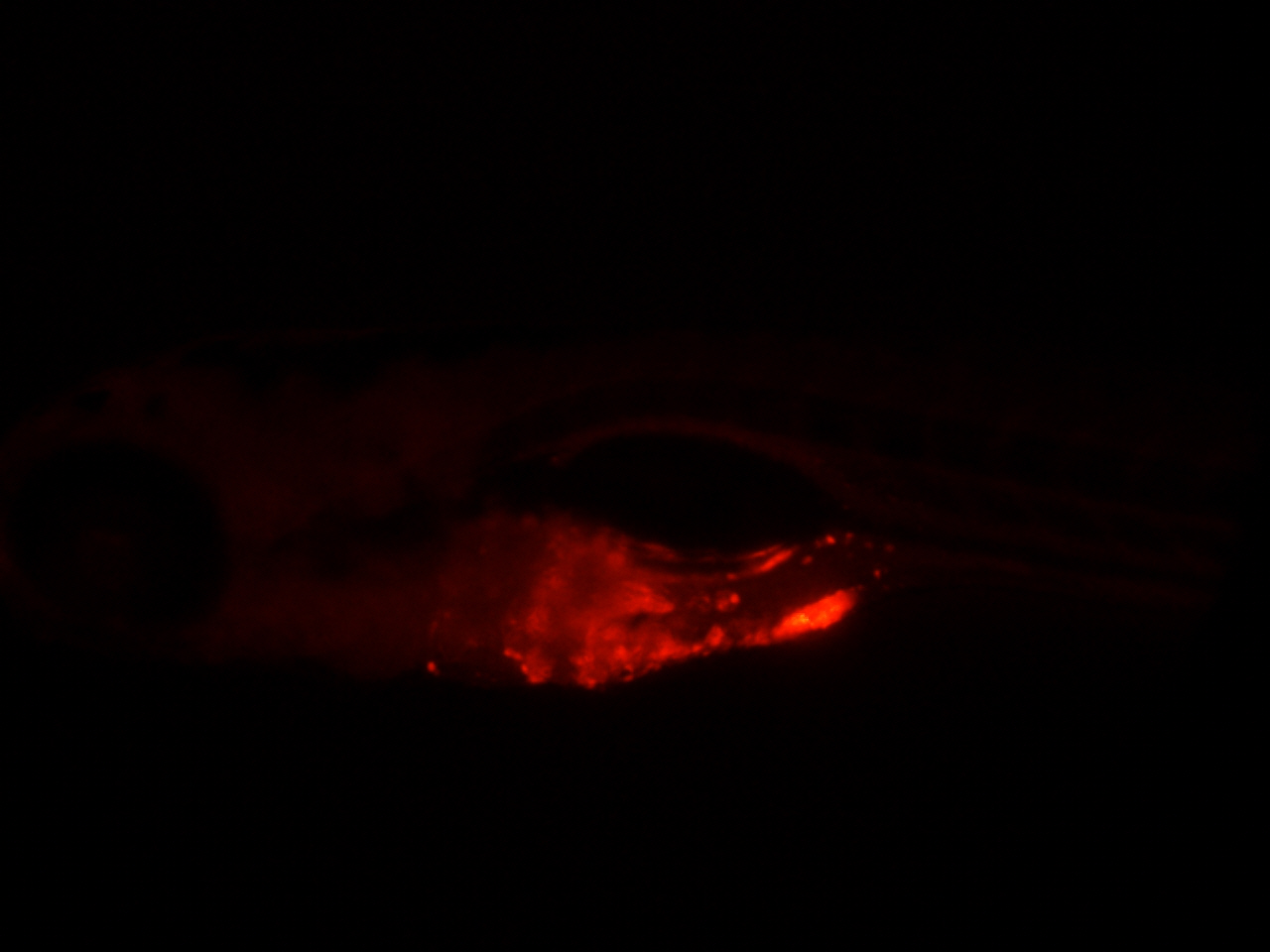

Supplement: Supplementary file 1 [file Data_Sheet_1.ZIP › model/12.jpg]

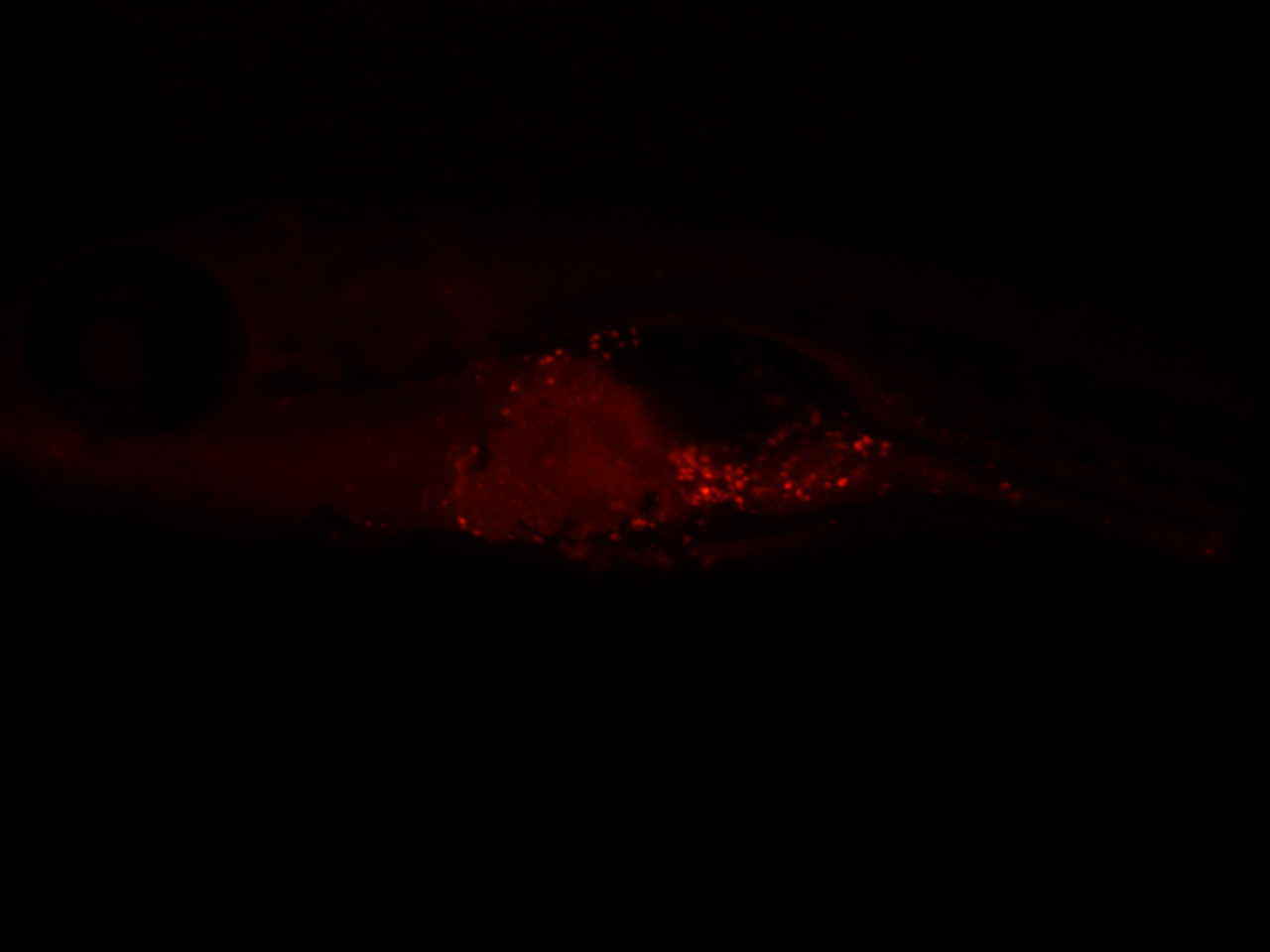

Supplement: Supplementary file 1 [file Data_Sheet_1.ZIP › model/13.jpg]

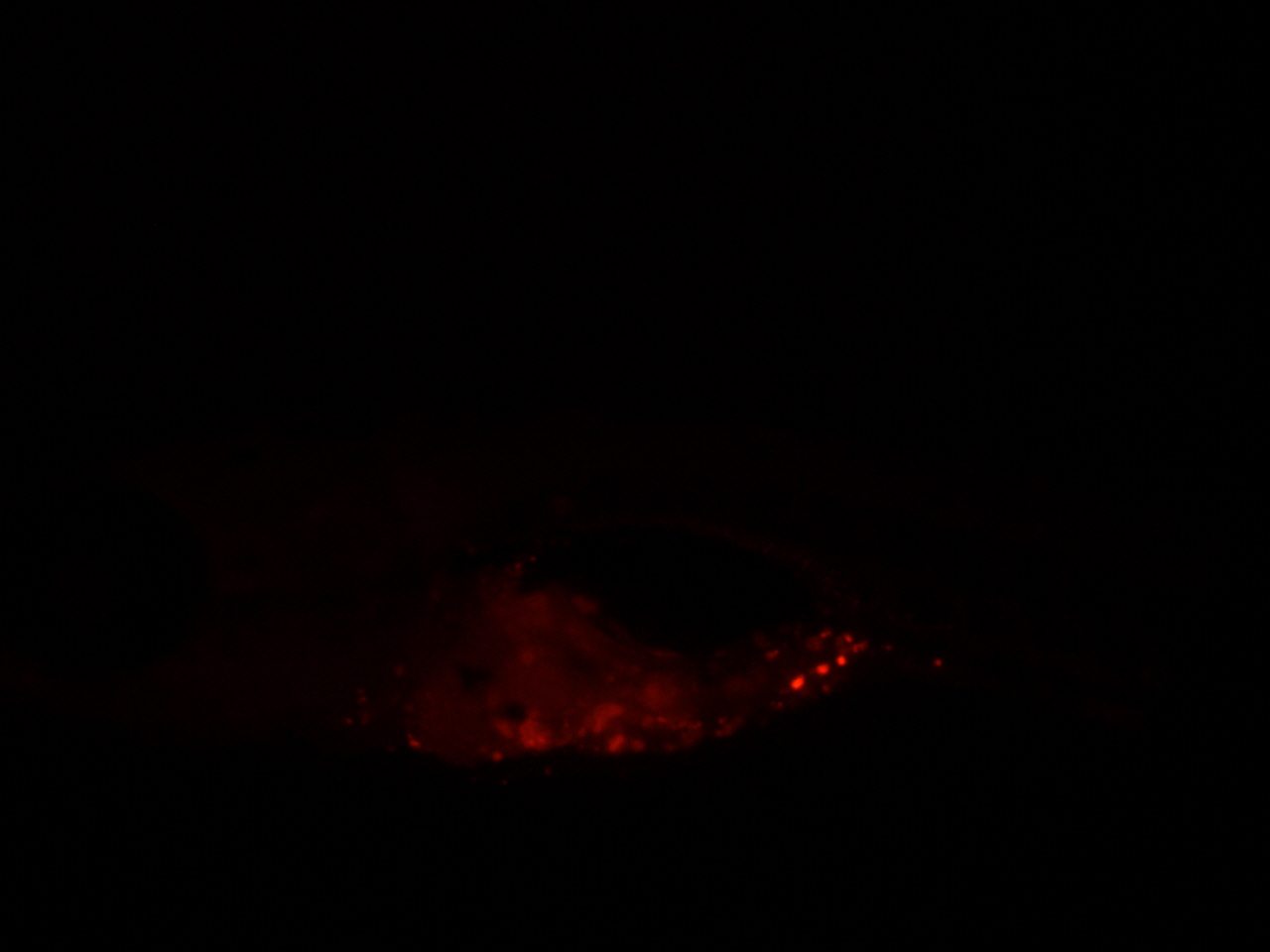

Supplement: Supplementary file 1 [file Data_Sheet_1.ZIP › model/14.jpg]

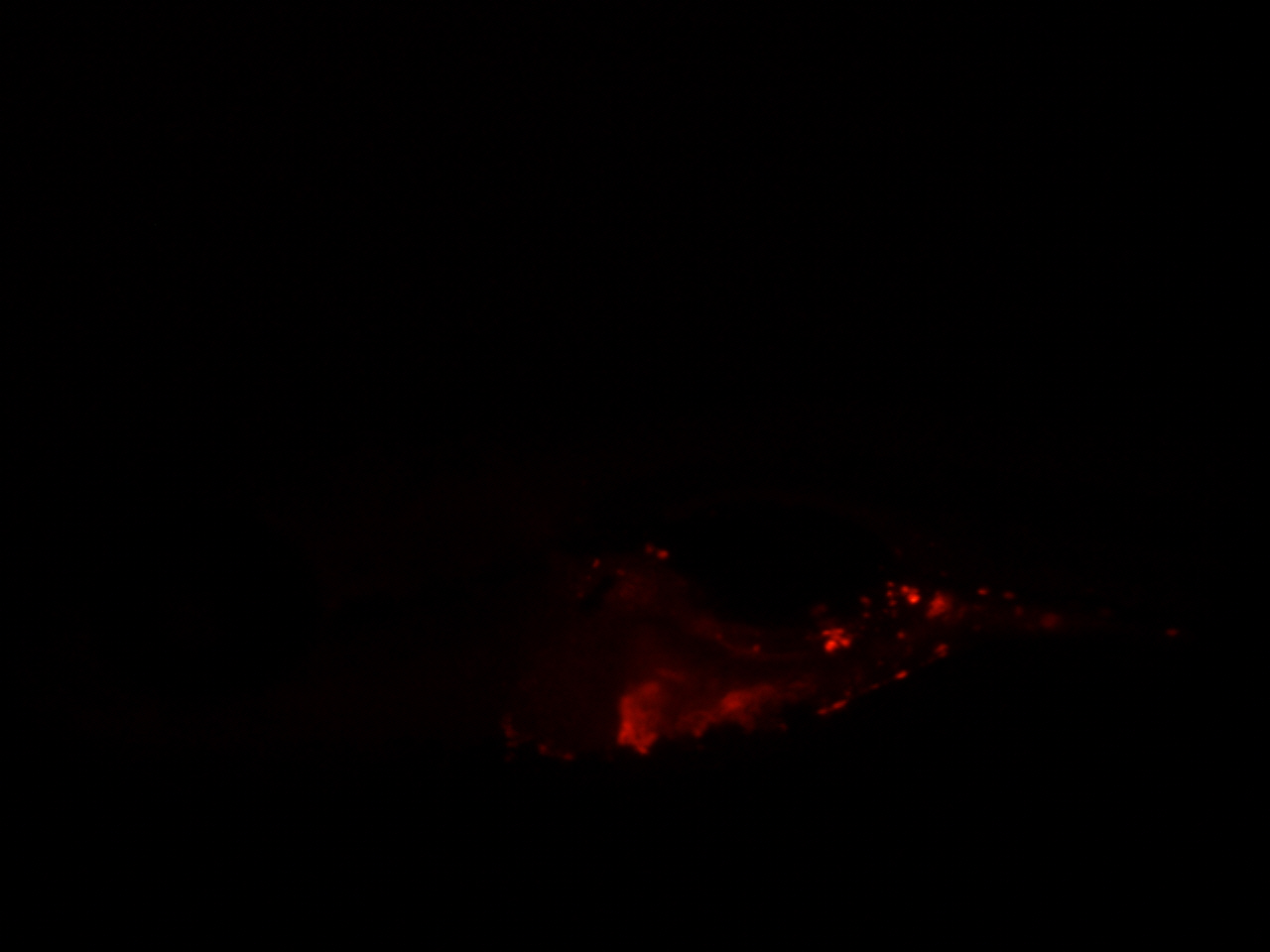

Supplement: Supplementary file 1 [file Data_Sheet_1.ZIP › model/15.jpg]

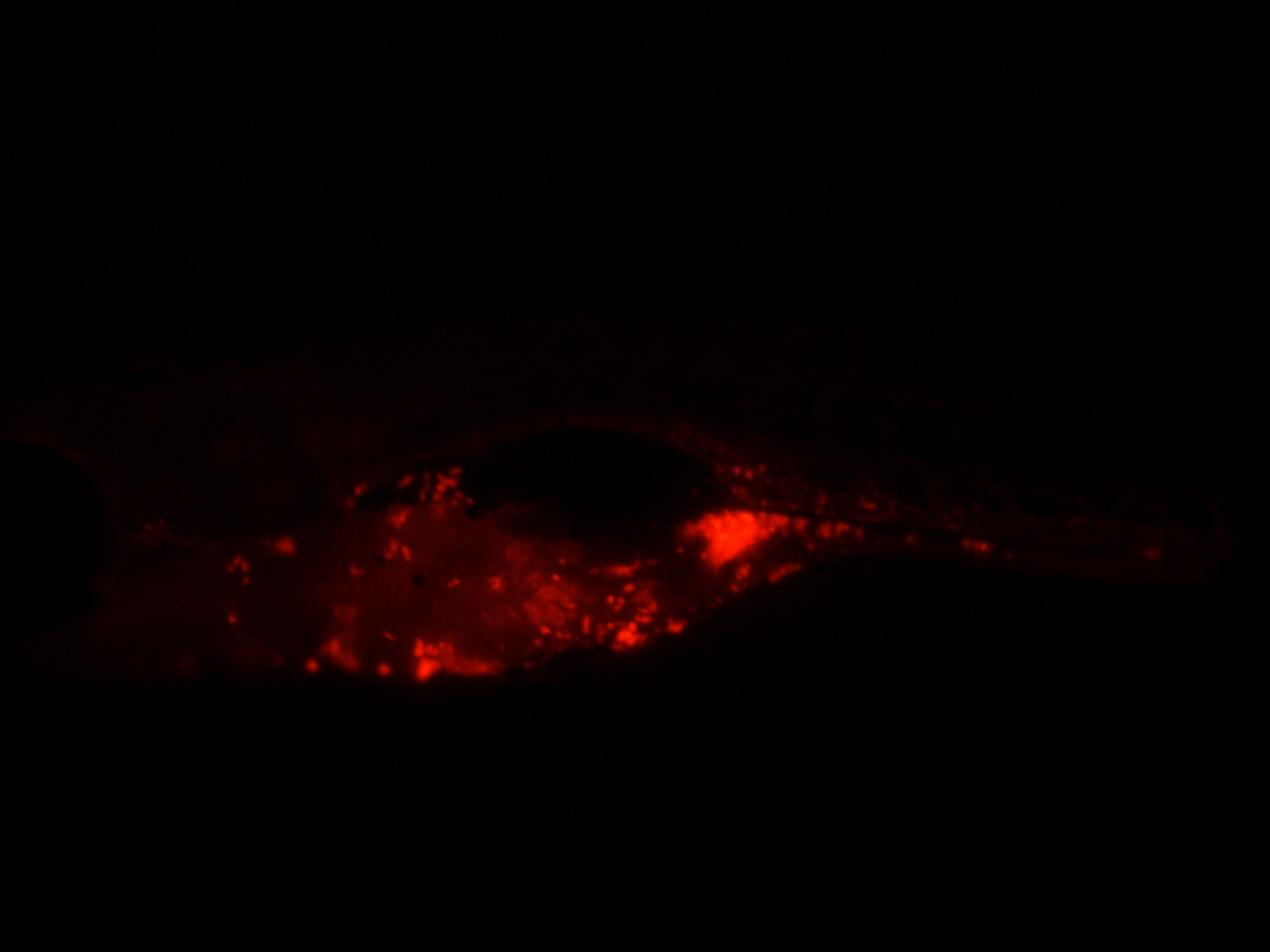

Supplement: Supplementary file 1 [file Data_Sheet_1.ZIP › model/16.jpg]

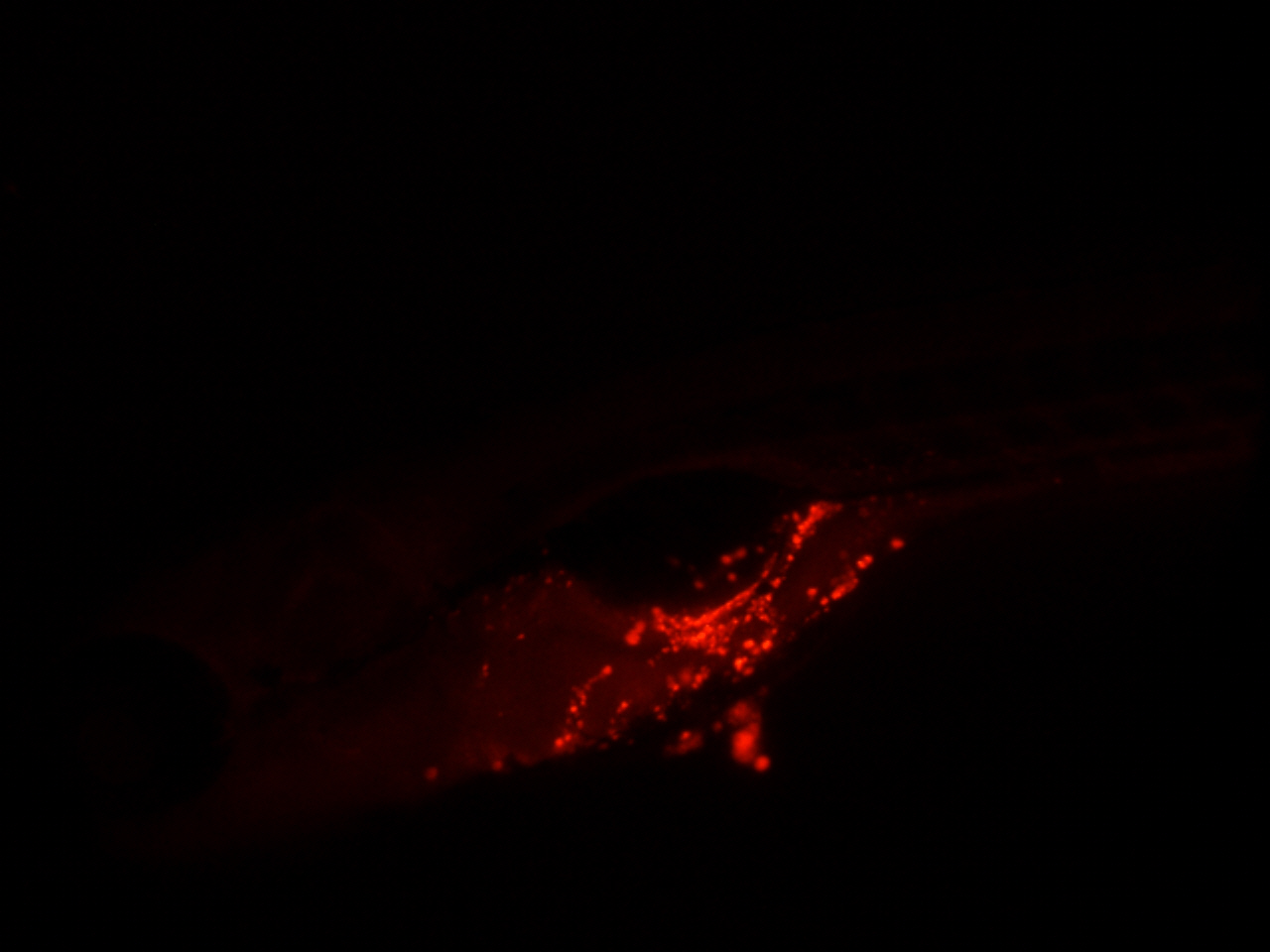

Supplement: Supplementary file 1 [file Data_Sheet_1.ZIP › model/2-1.jpg]

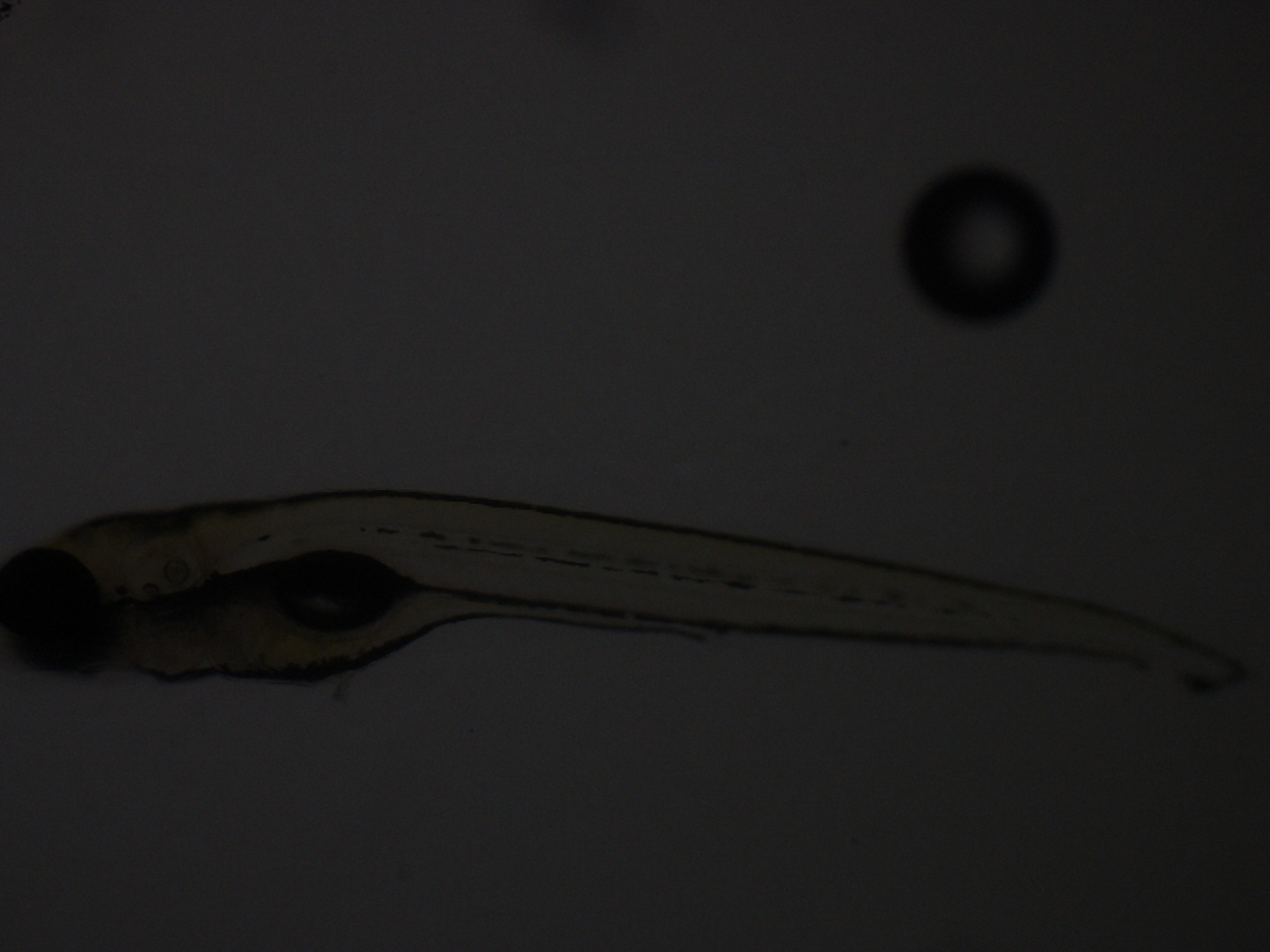

Supplement: Supplementary file 1 [file Data_Sheet_1.ZIP › model/2-2.jpg]

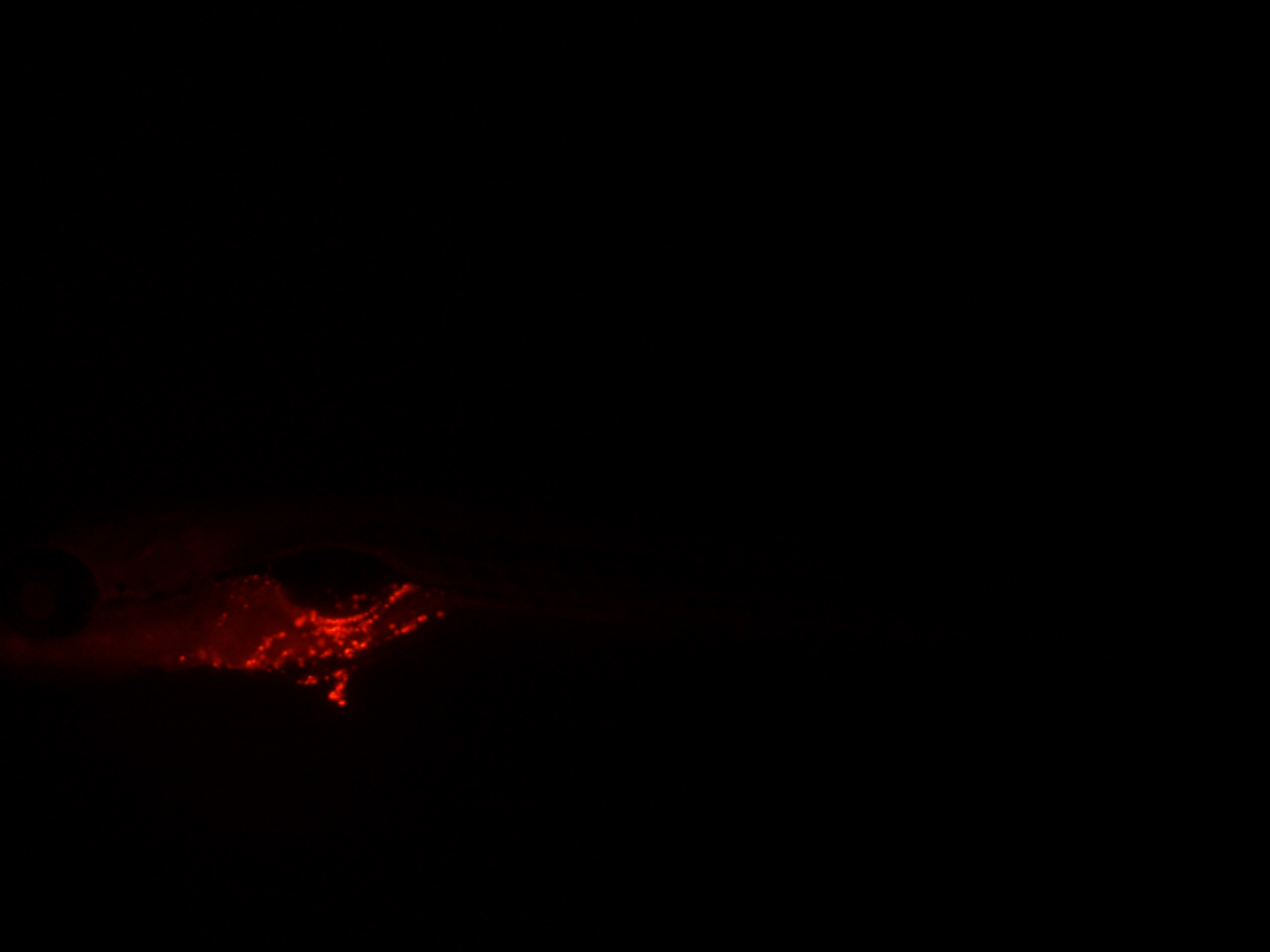

Supplement: Supplementary file 1 [file Data_Sheet_1.ZIP › model/2-3.jpg]

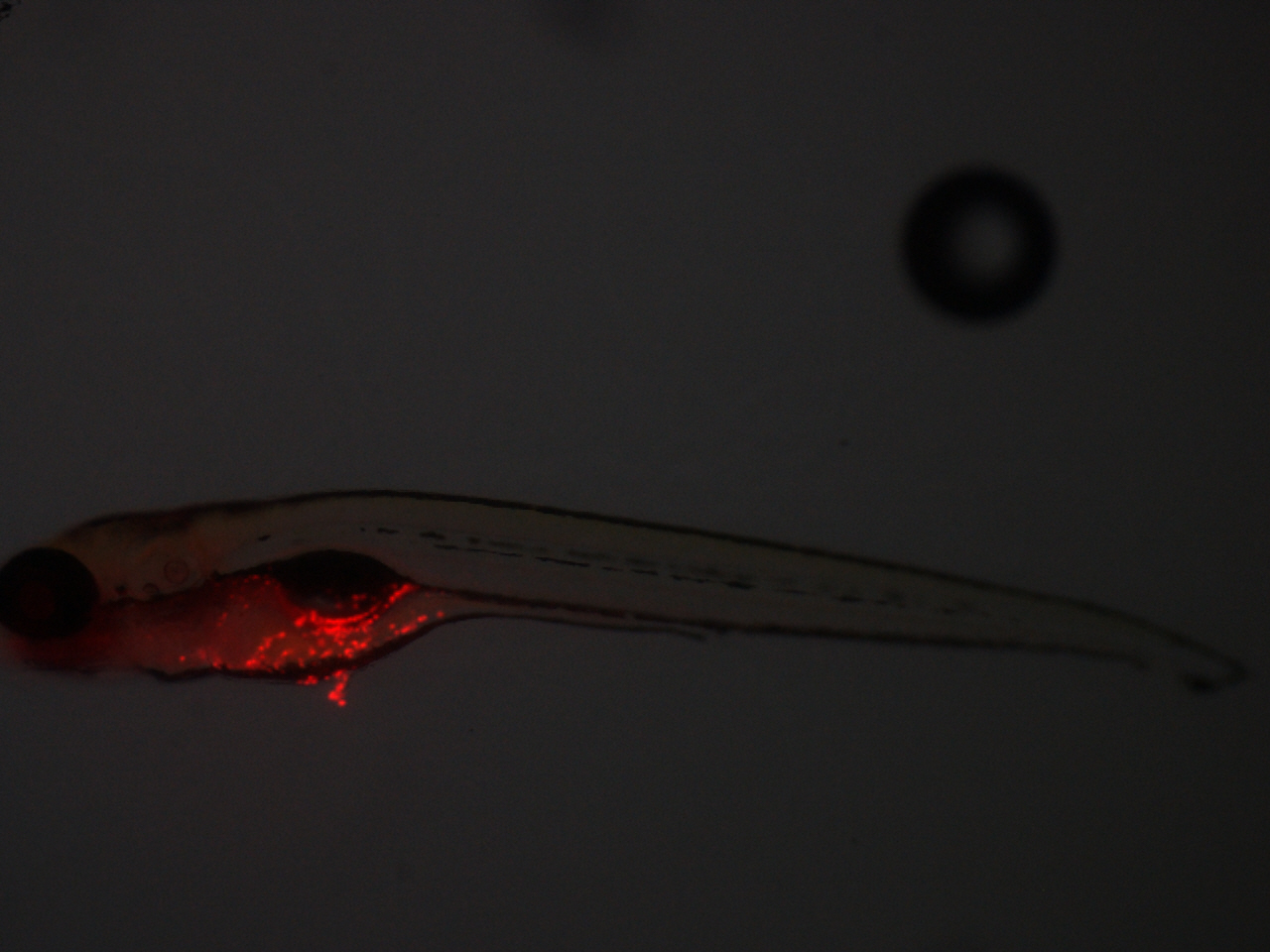

Supplement: Supplementary file 1 [file Data_Sheet_1.ZIP › model/2-4.jpg]

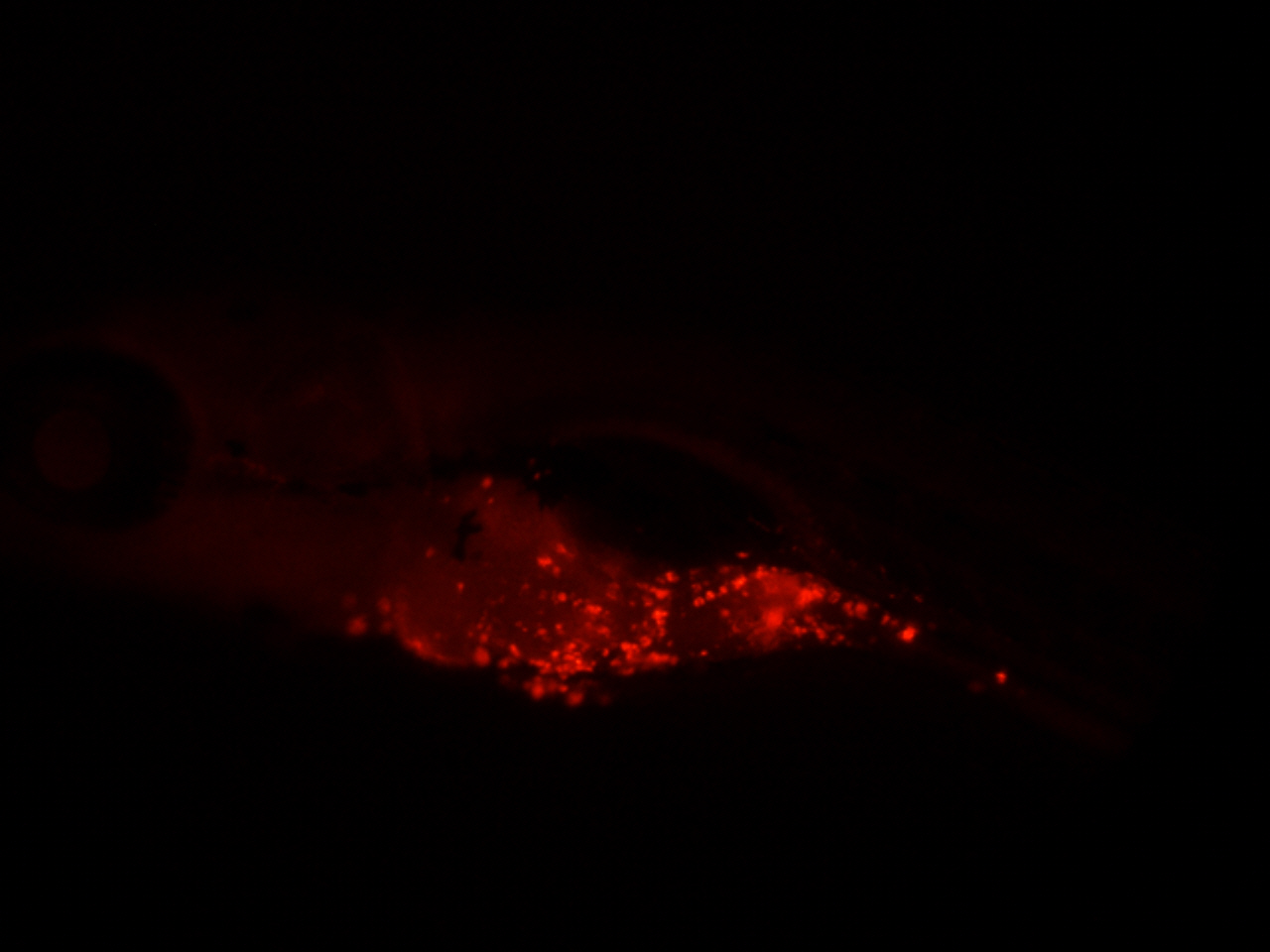

Supplement: Supplementary file 1 [file Data_Sheet_1.ZIP › model/3.jpg]

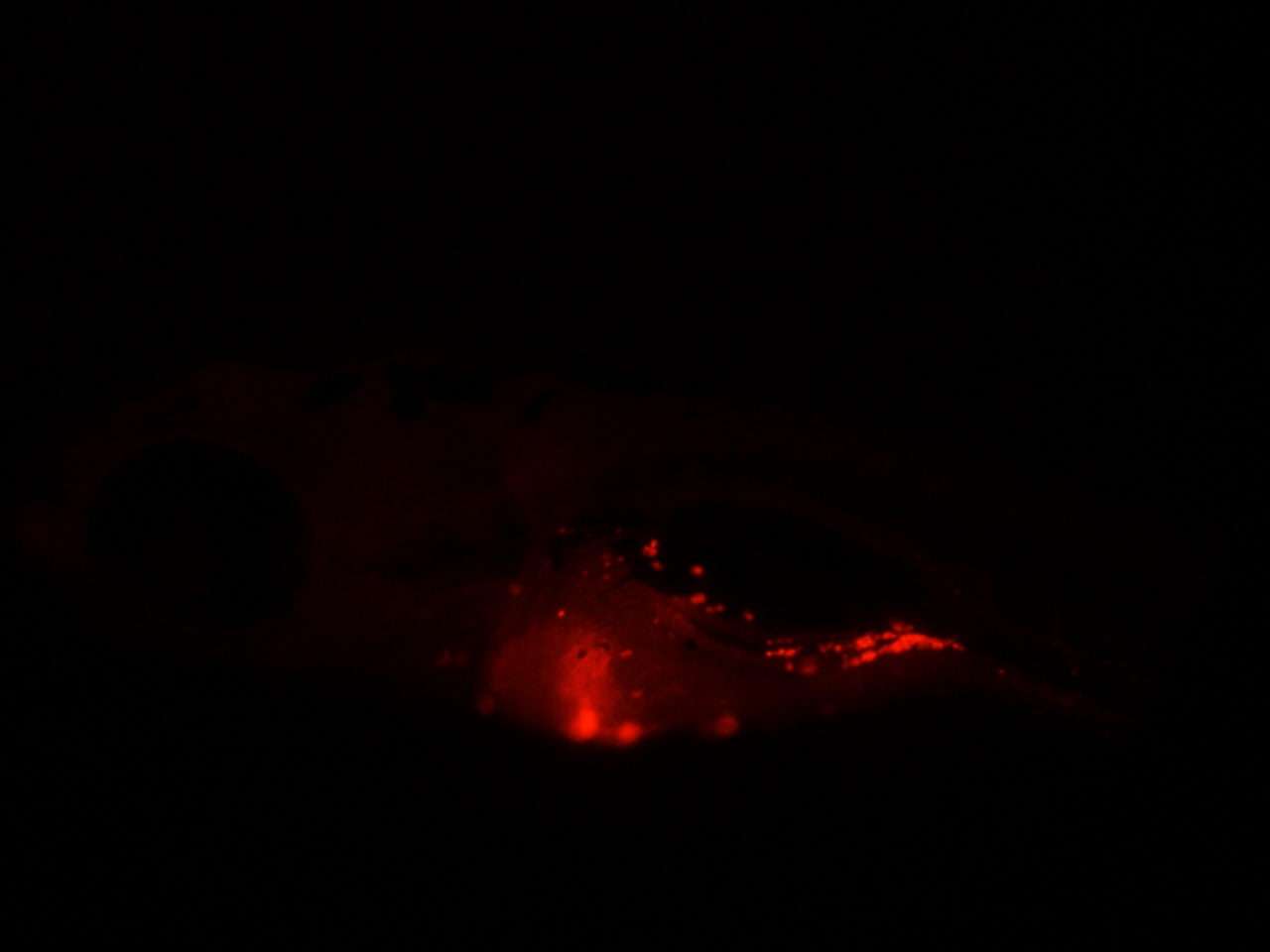

Supplement: Supplementary file 1 [file Data_Sheet_1.ZIP › model/4.jpg]

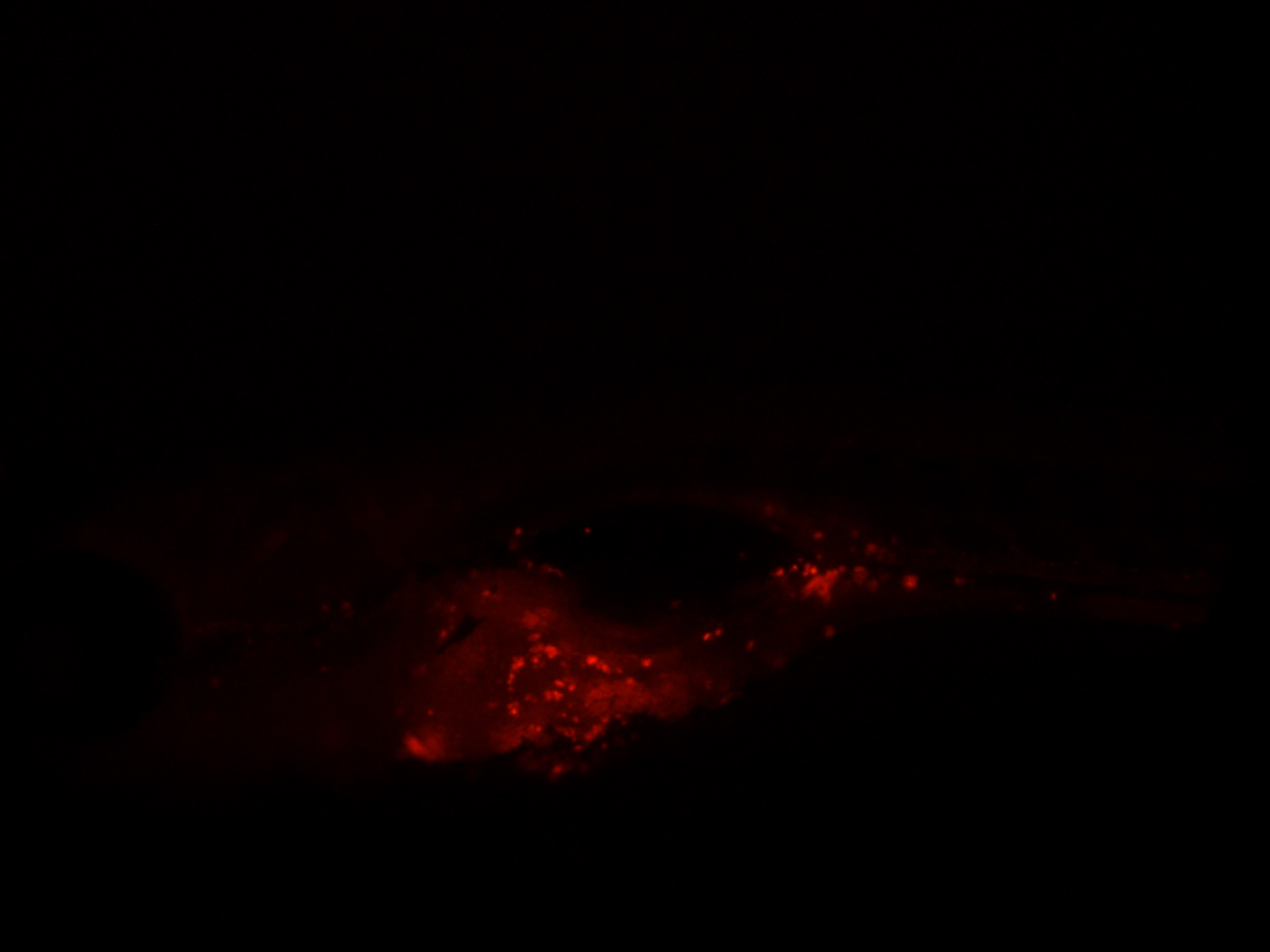

Supplement: Supplementary file 1 [file Data_Sheet_1.ZIP › model/5.jpg]

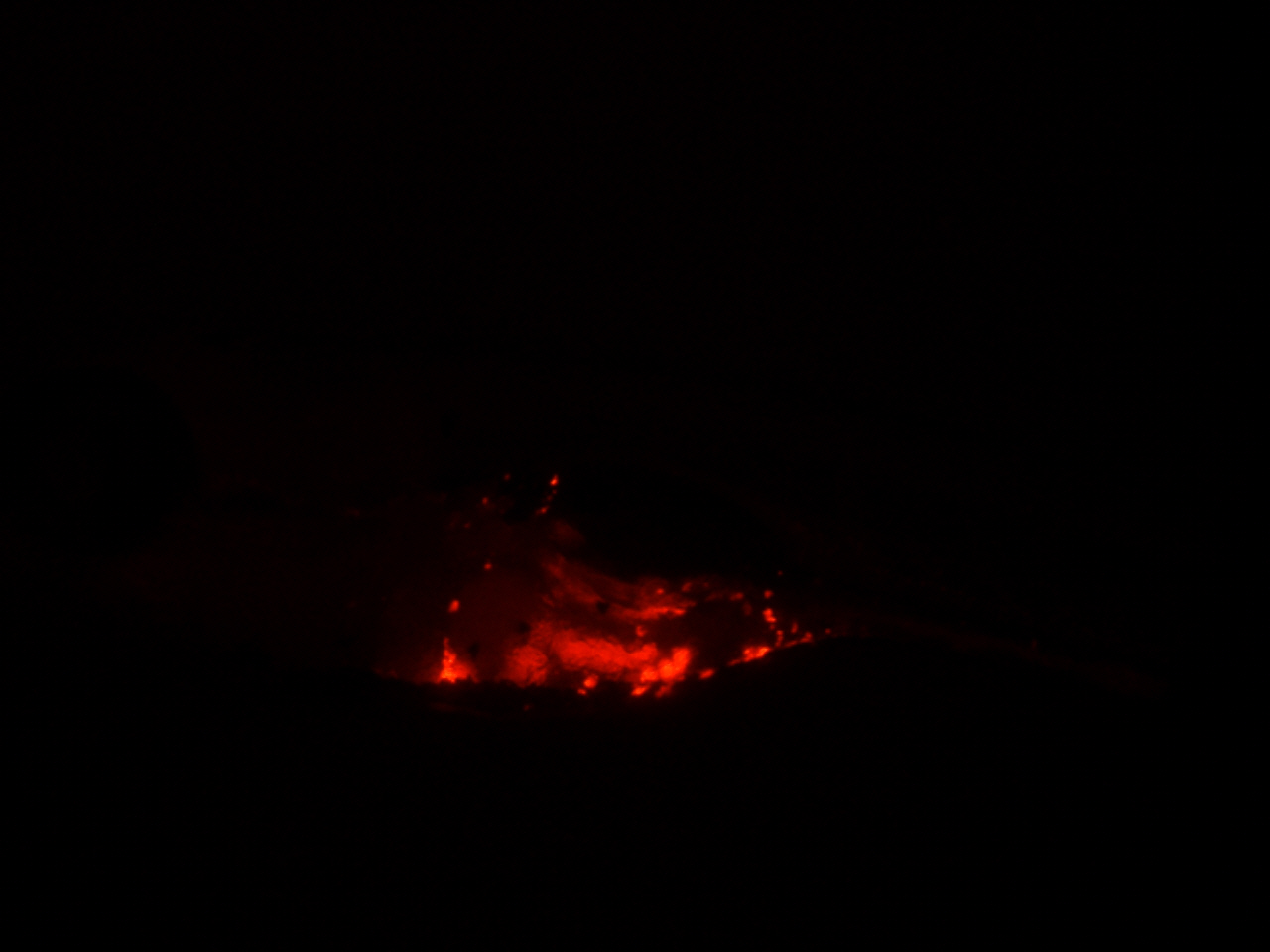

Supplement: Supplementary file 1 [file Data_Sheet_1.ZIP › model/6.jpg]

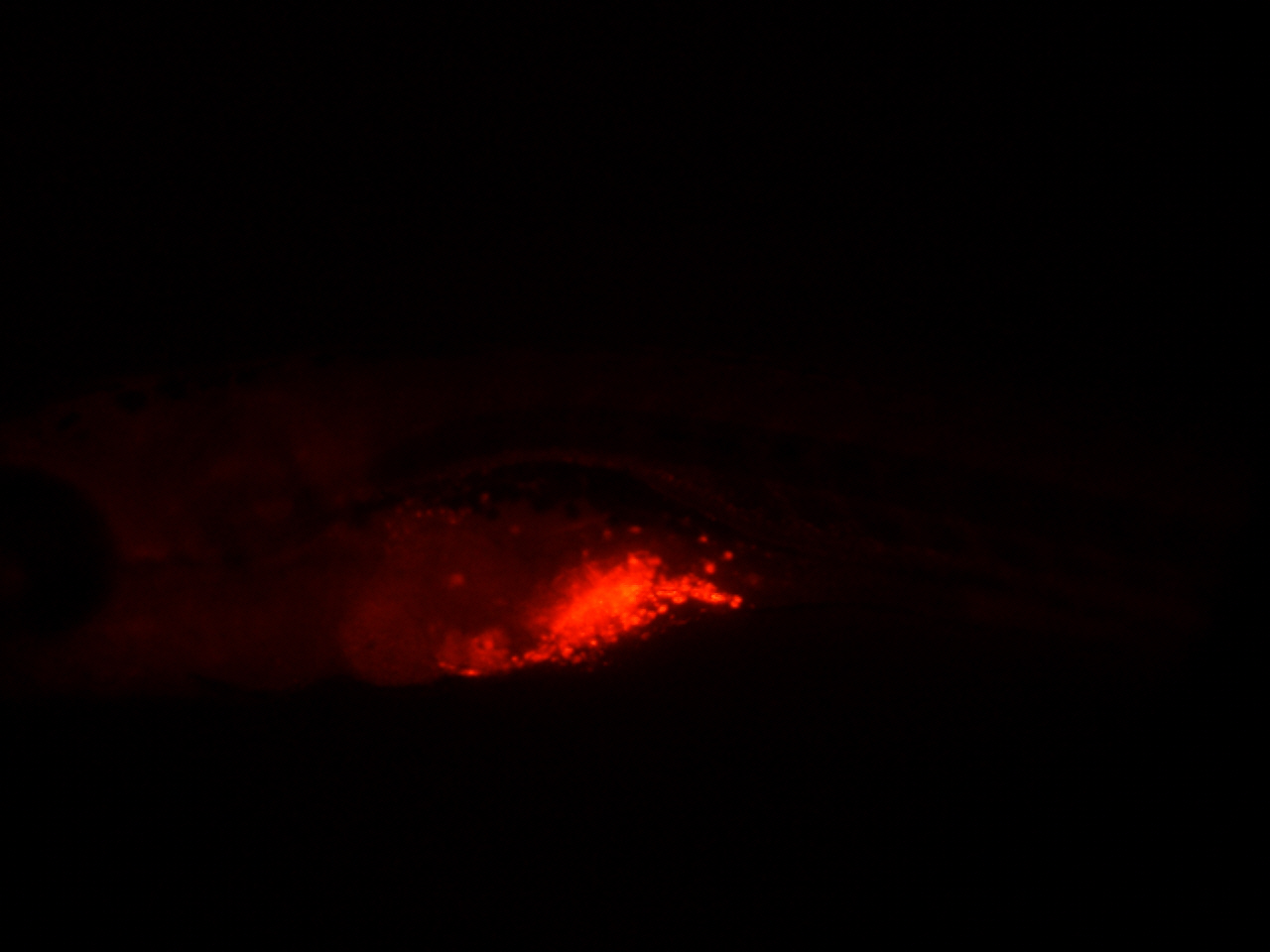

Supplement: Supplementary file 1 [file Data_Sheet_1.ZIP › model/7.jpg]

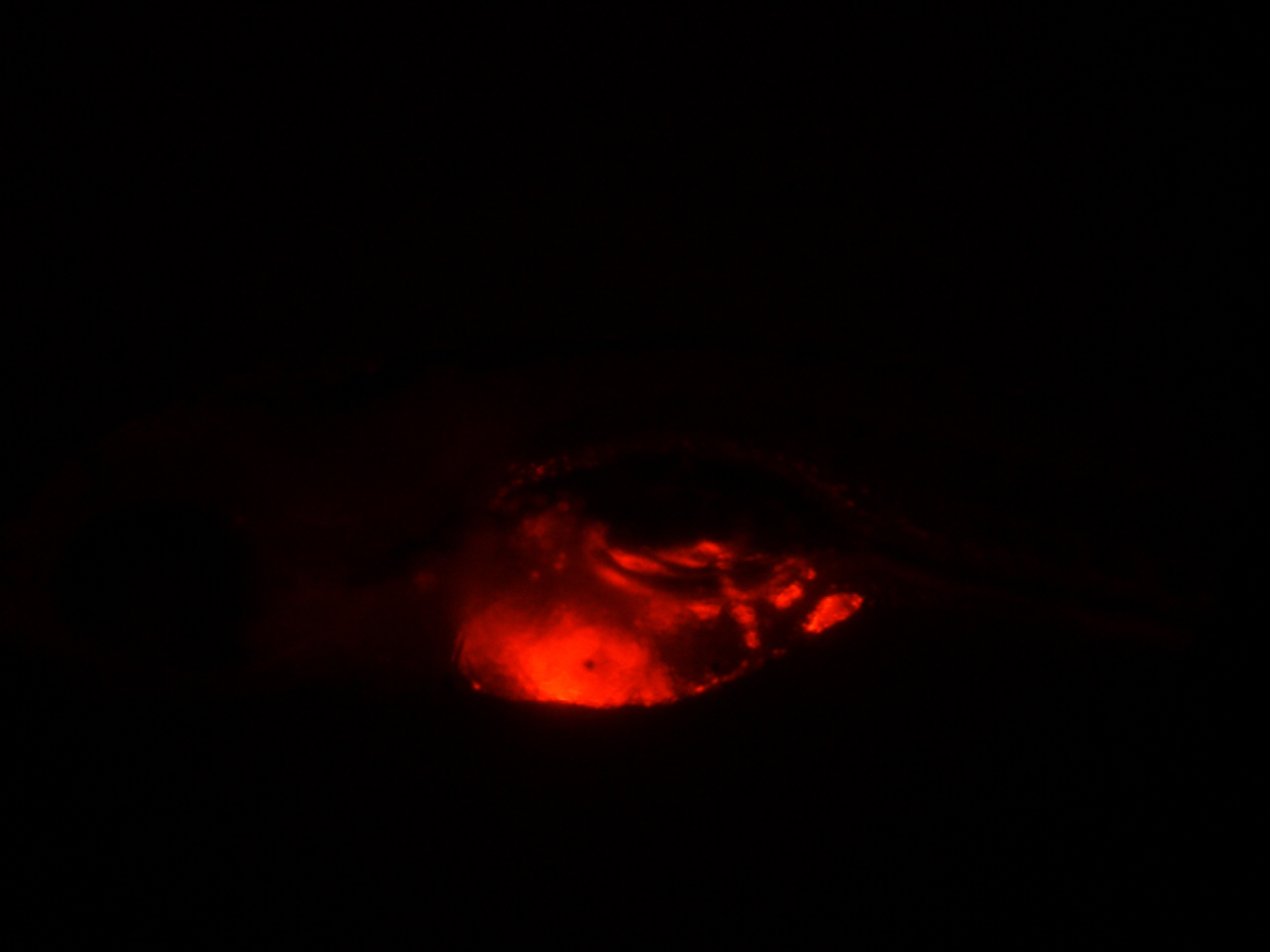

Supplement: Supplementary file 1 [file Data_Sheet_1.ZIP › model/8.jpg]

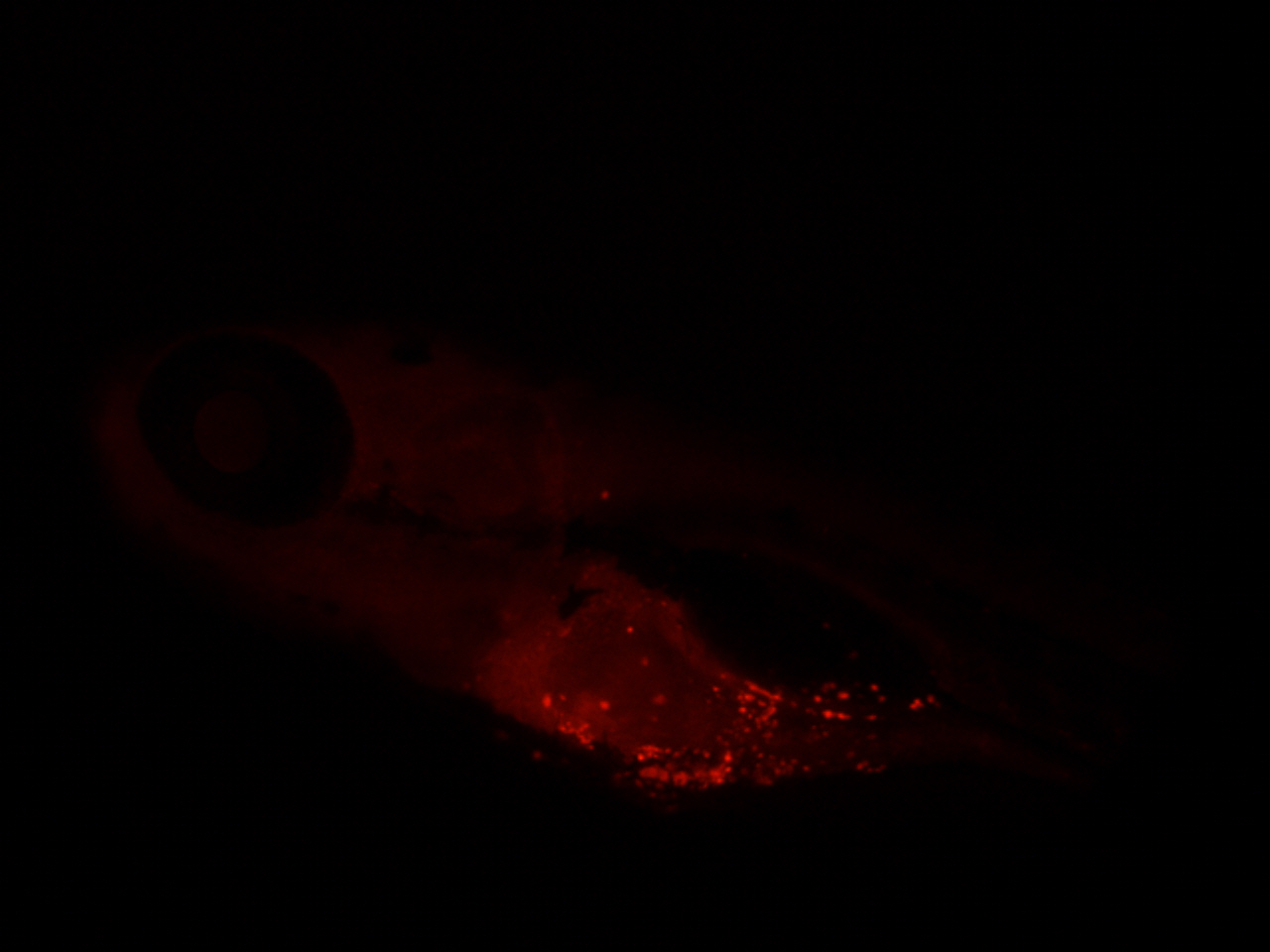

Supplement: Supplementary file 1 [file Data_Sheet_1.ZIP › model/9.jpg]

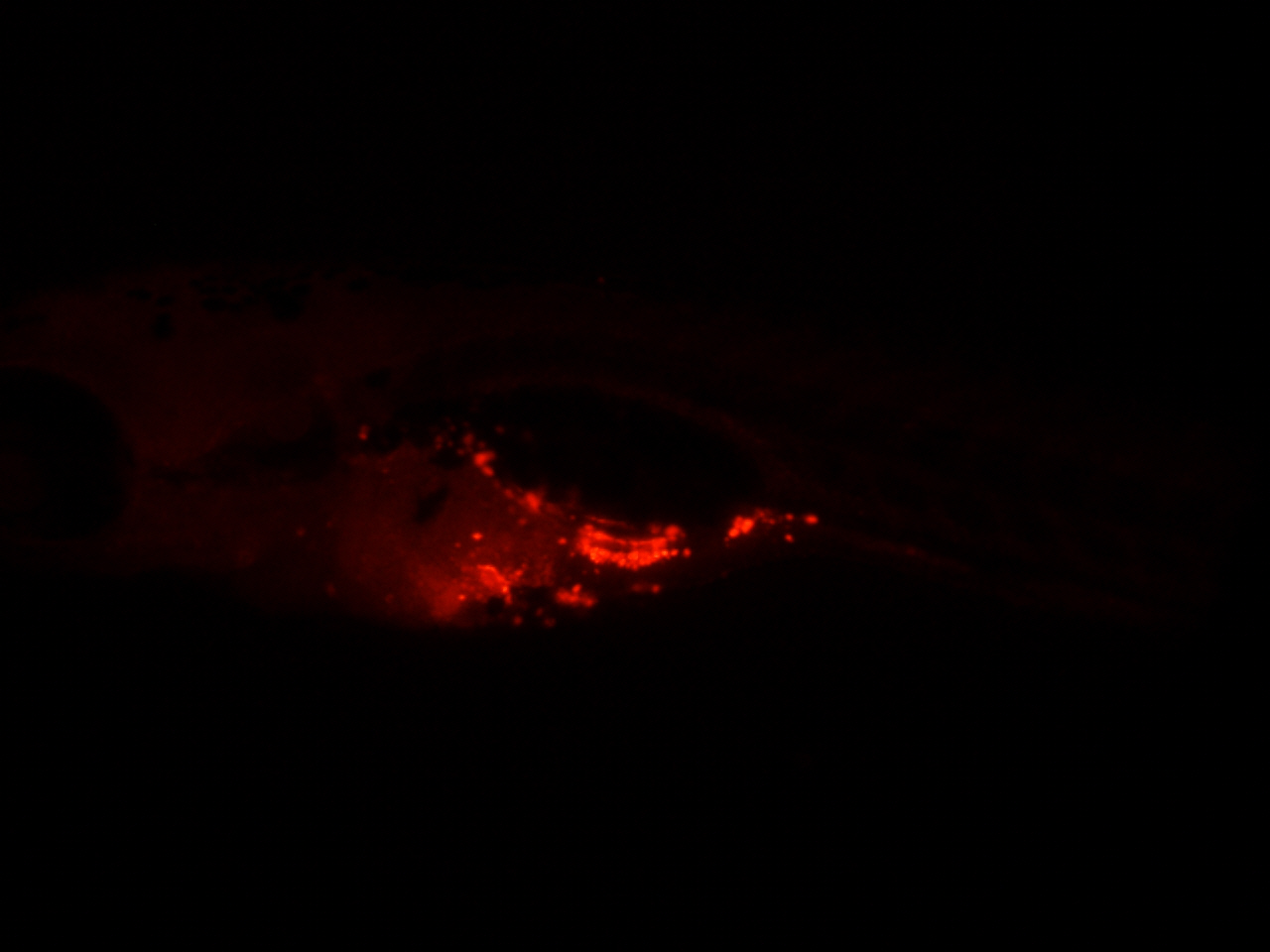

Supplement: Supplementary file 1 [file Data_Sheet_1.ZIP › peptide/1.jpg]

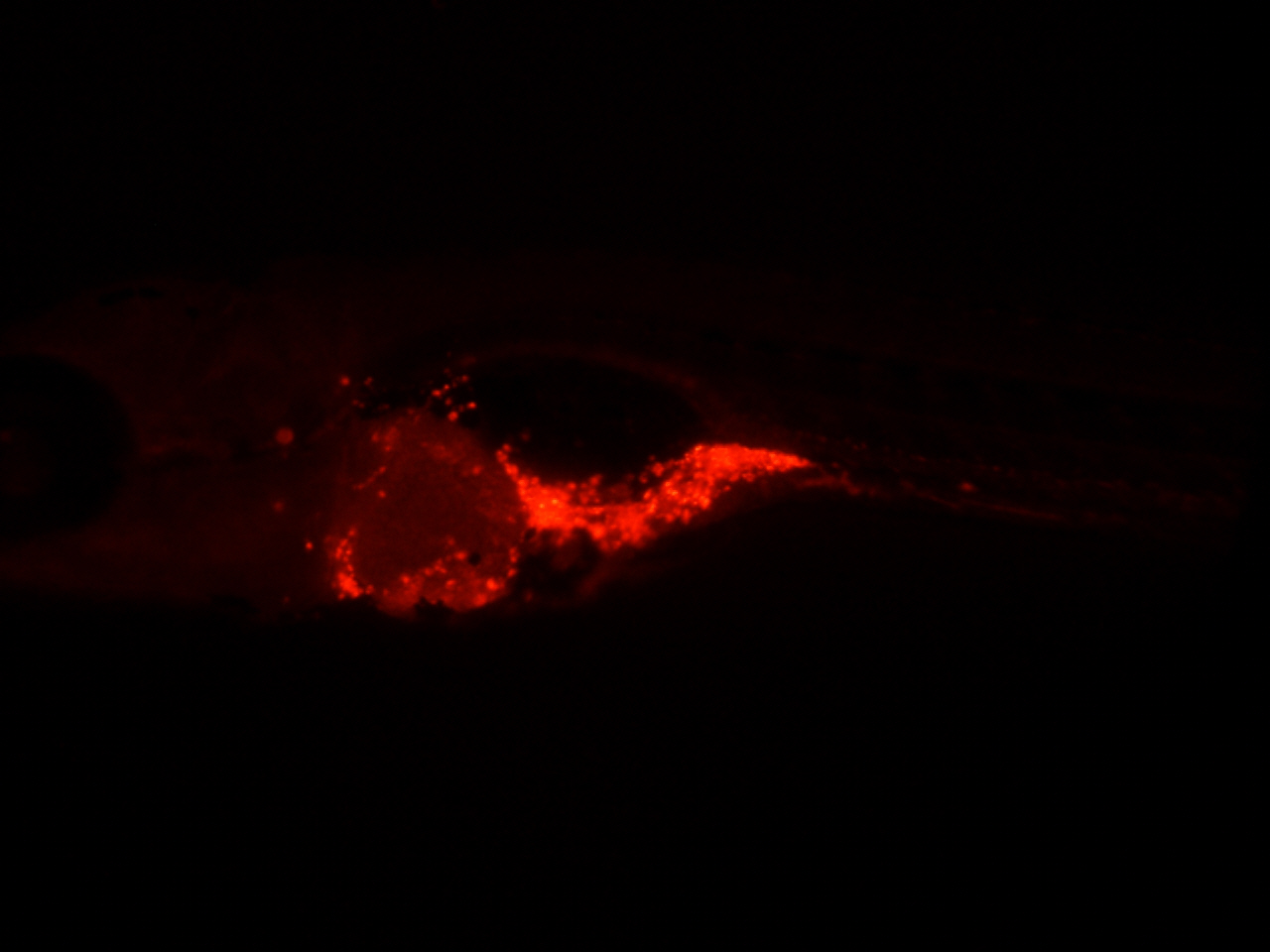

Supplement: Supplementary file 1 [file Data_Sheet_1.ZIP › peptide/10.jpg]

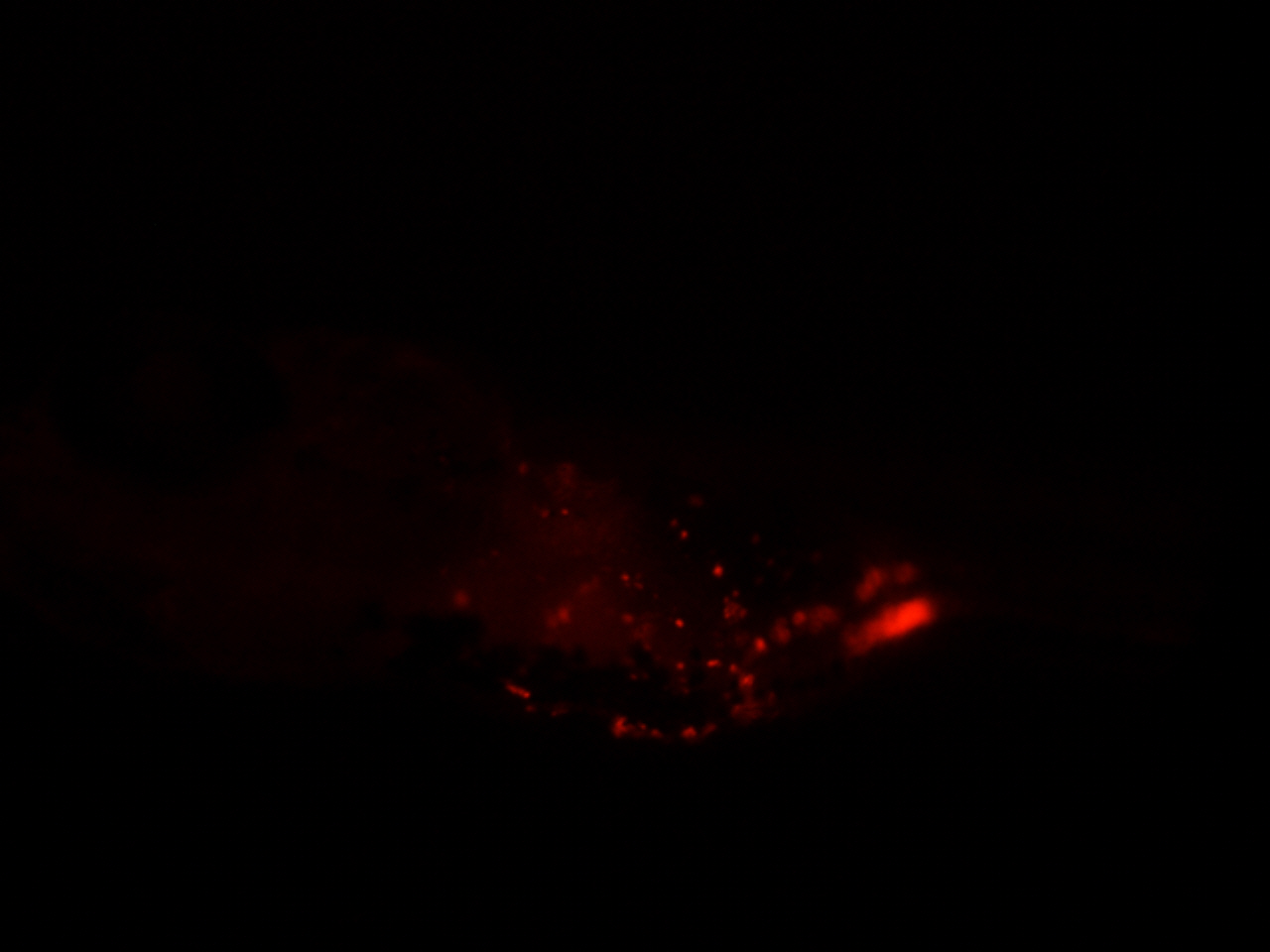

Supplement: Supplementary file 1 [file Data_Sheet_1.ZIP › peptide/11.jpg]

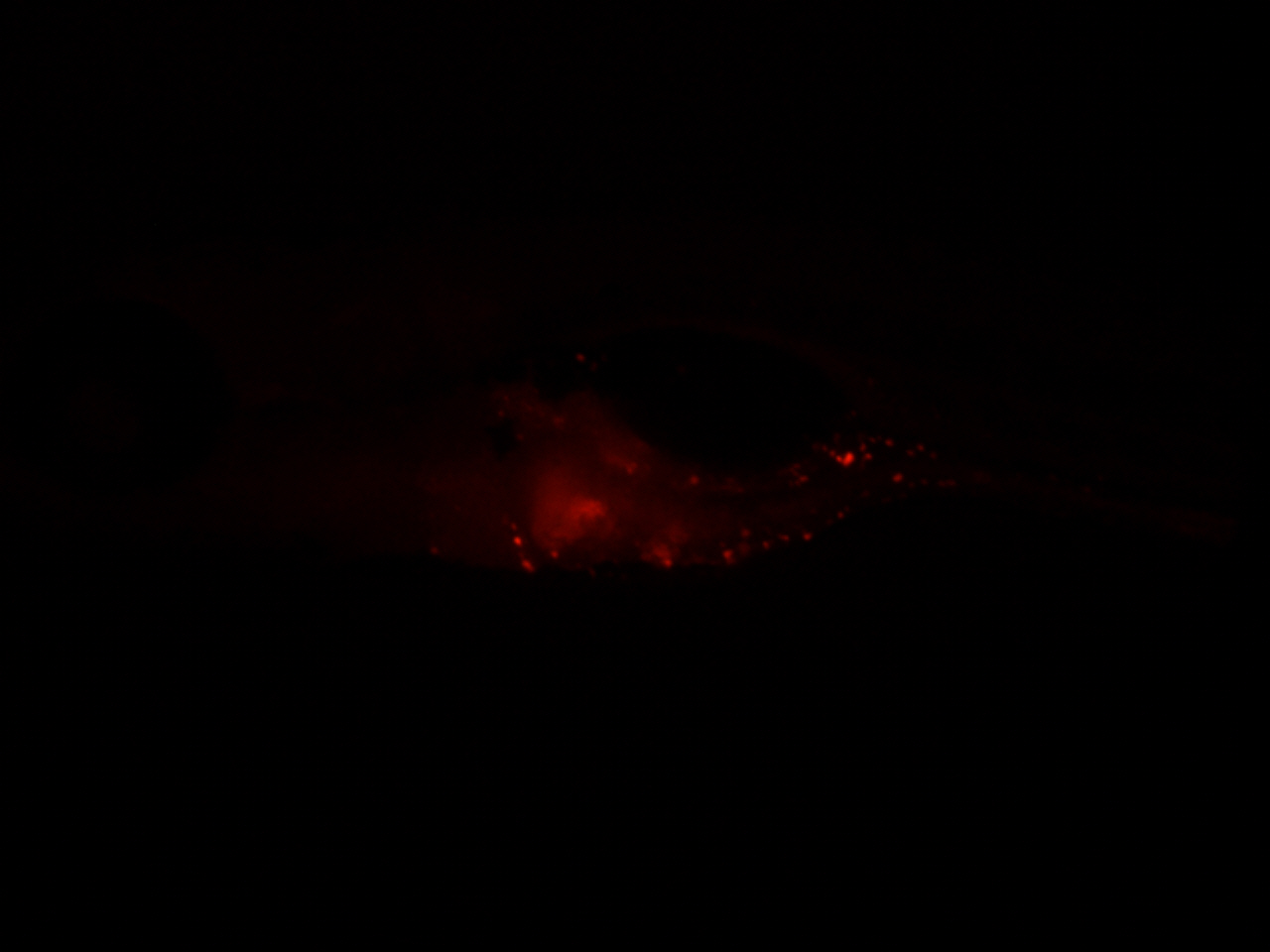

Supplement: Supplementary file 1 [file Data_Sheet_1.ZIP › peptide/12.jpg]

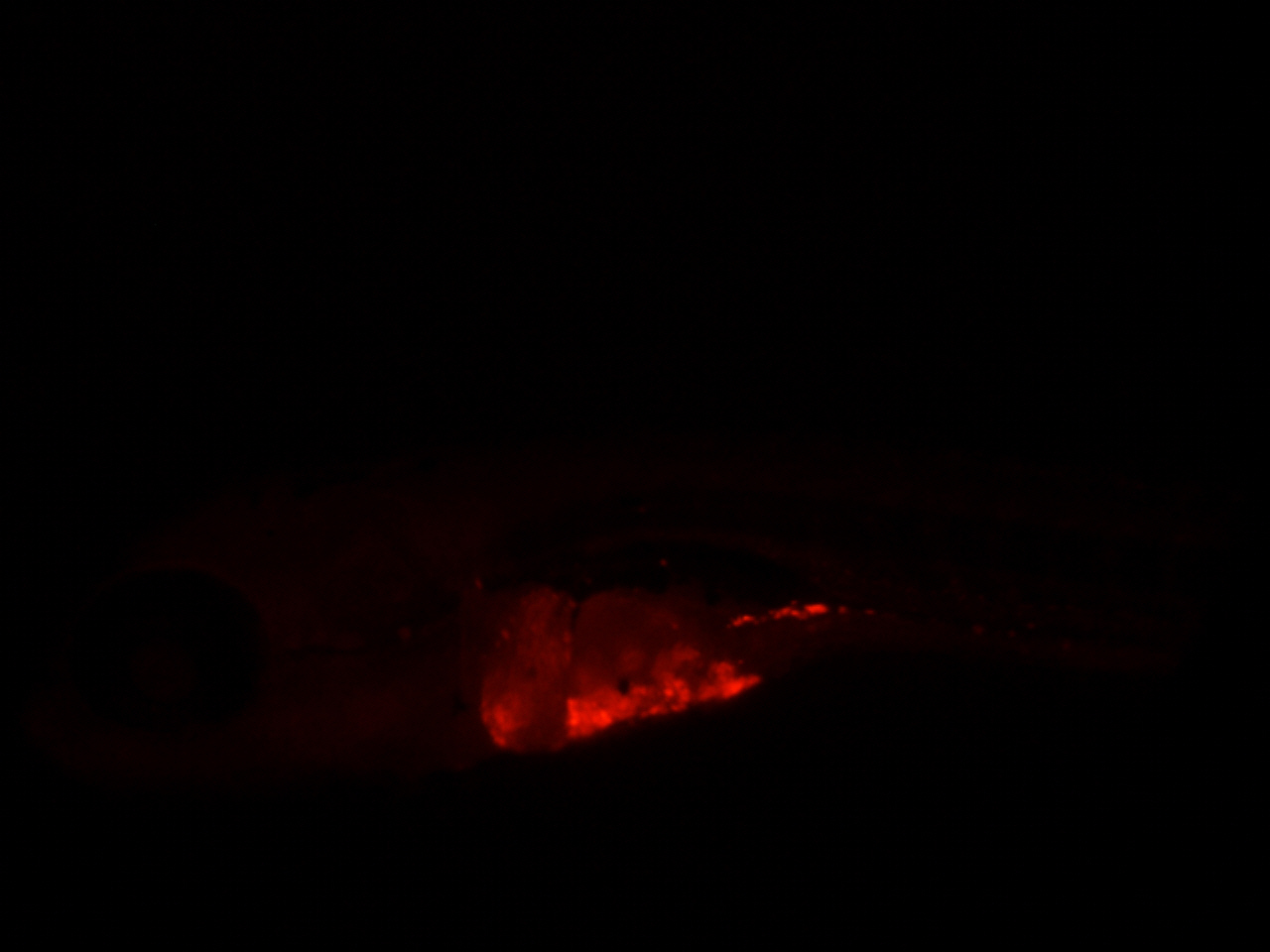

Supplement: Supplementary file 1 [file Data_Sheet_1.ZIP › peptide/13.jpg]

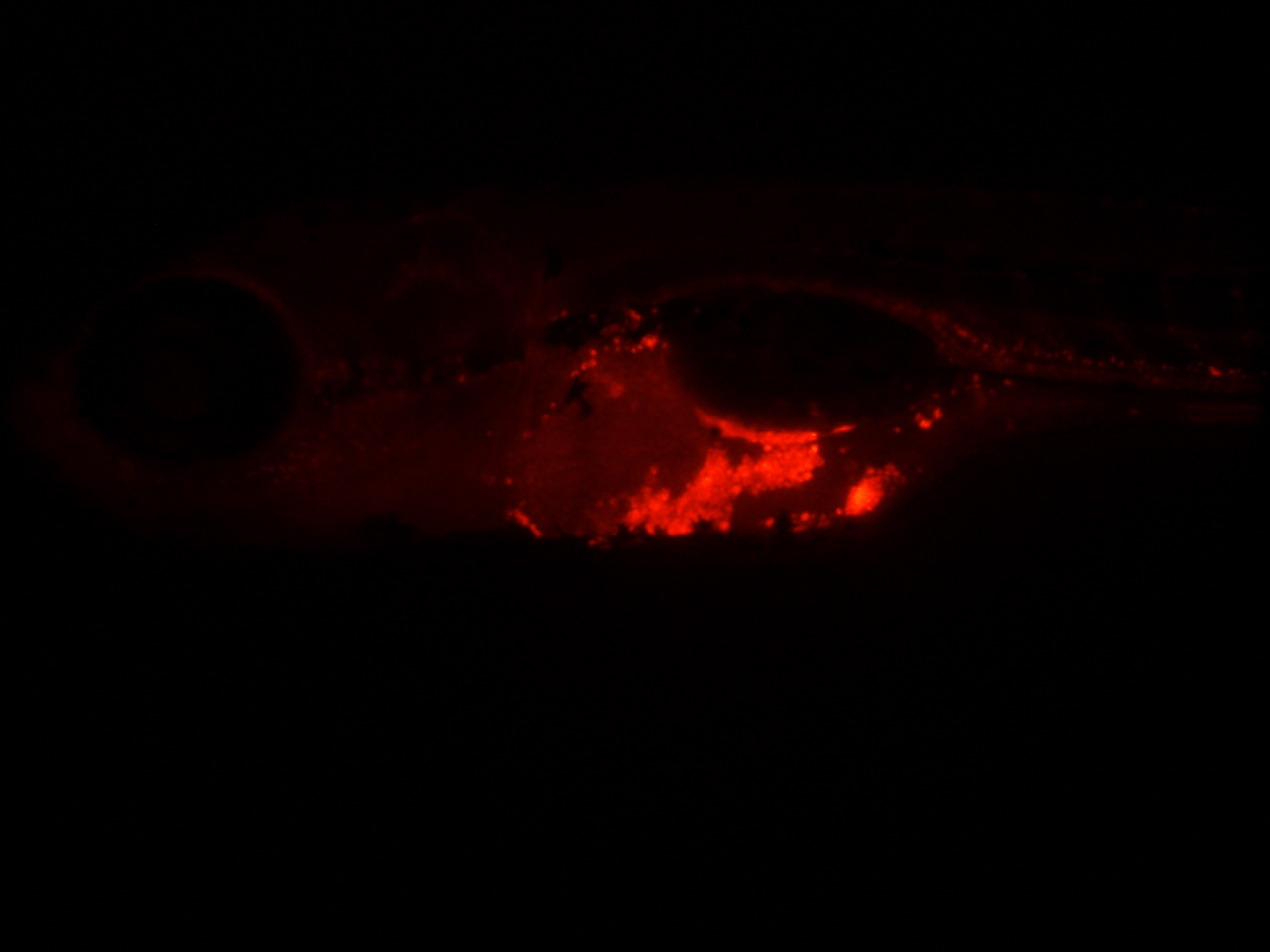

Supplement: Supplementary file 1 [file Data_Sheet_1.ZIP › peptide/14.jpg]

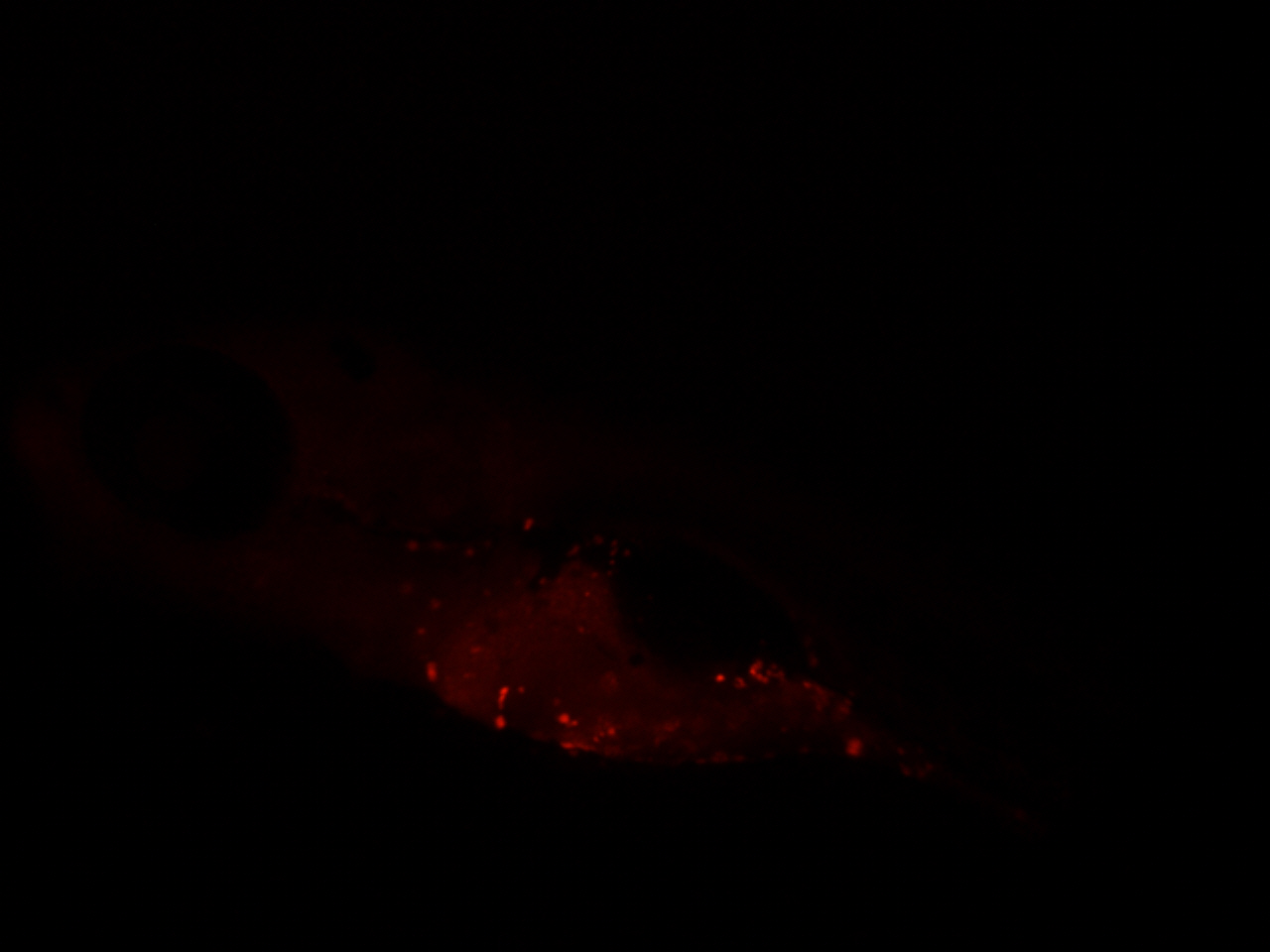

Supplement: Supplementary file 1 [file Data_Sheet_1.ZIP › peptide/2.jpg]

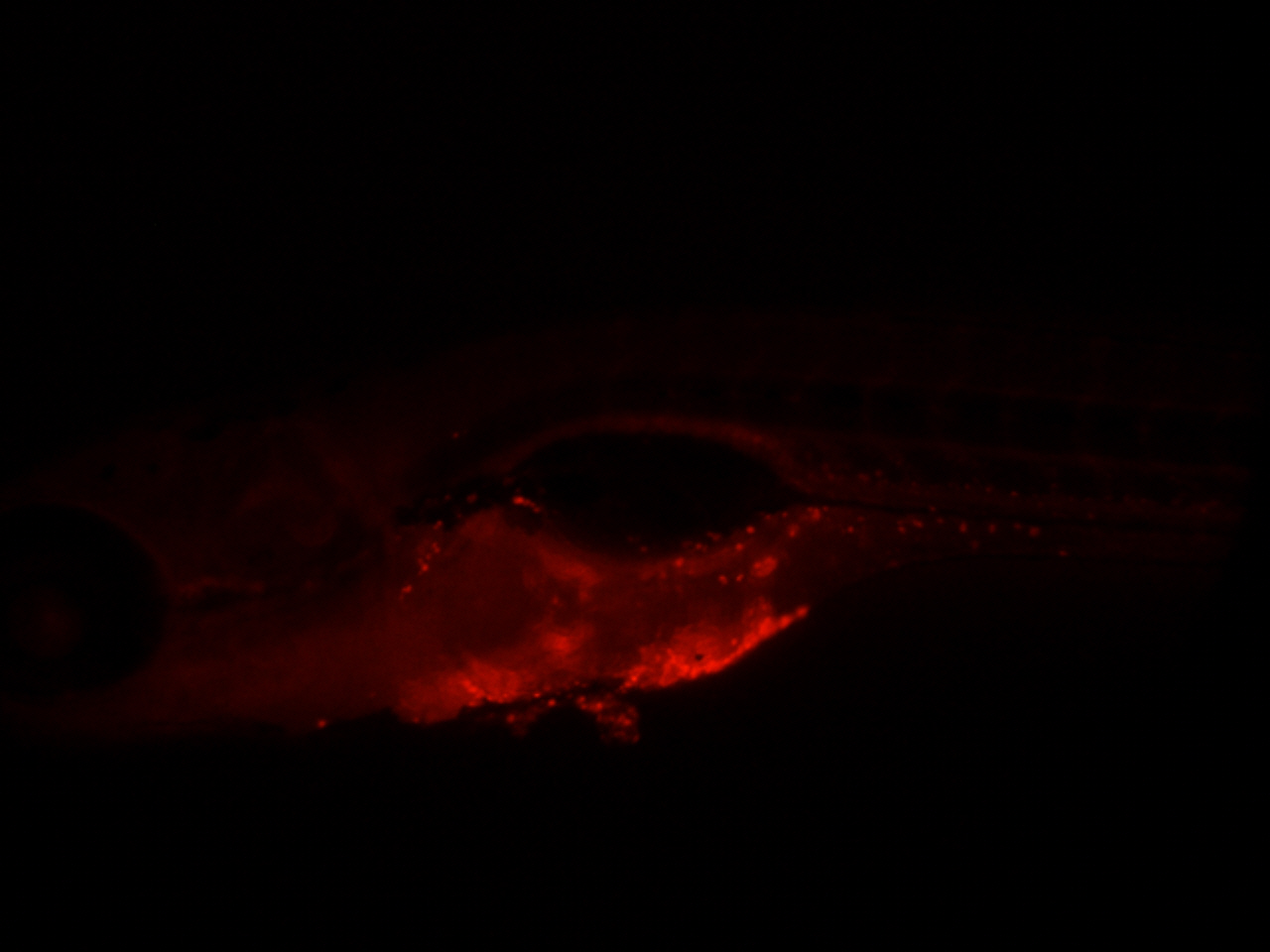

Supplement: Supplementary file 1 [file Data_Sheet_1.ZIP › peptide/4.jpg]

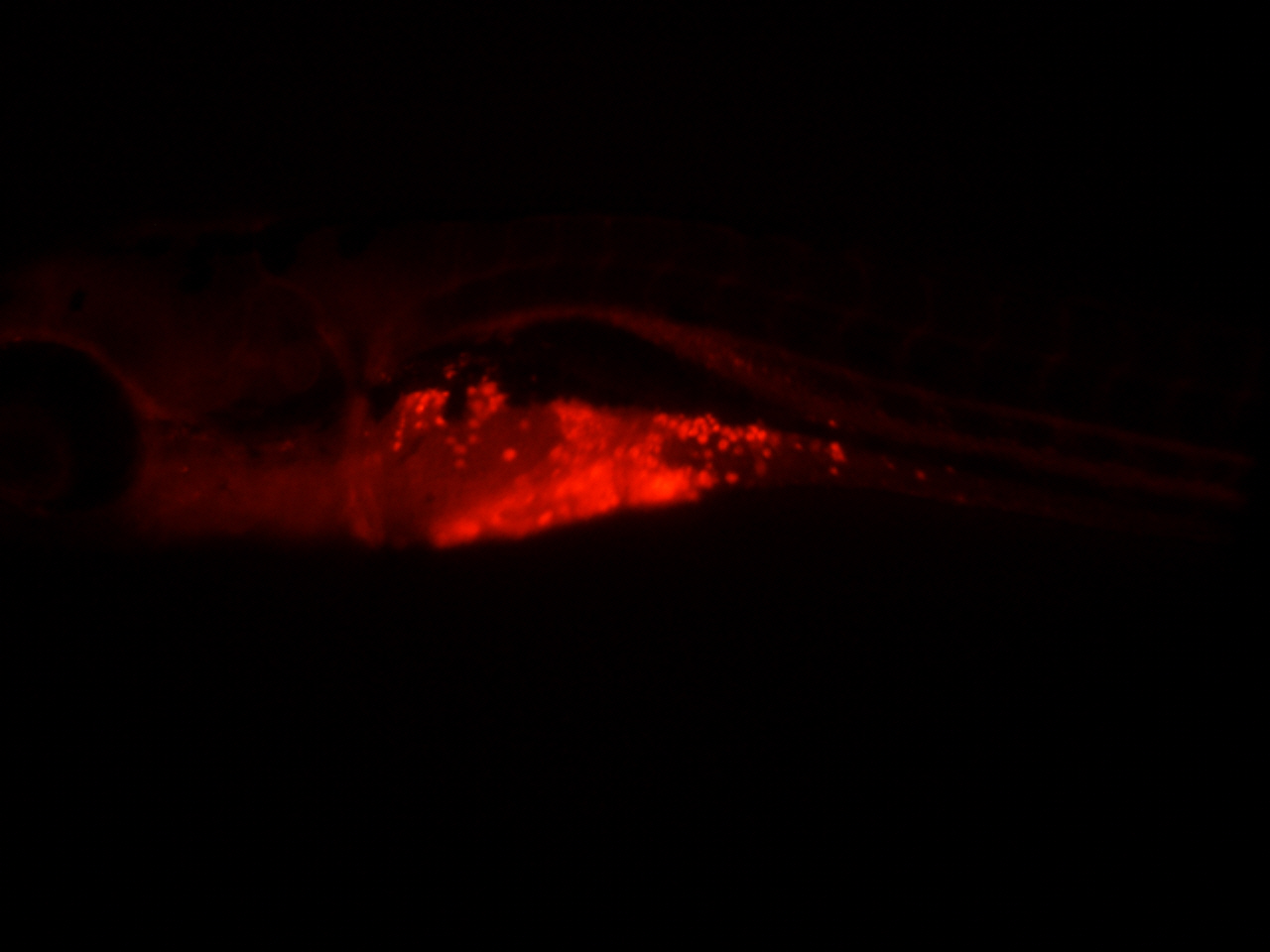

Supplement: Supplementary file 1 [file Data_Sheet_1.ZIP › peptide/5.jpg]

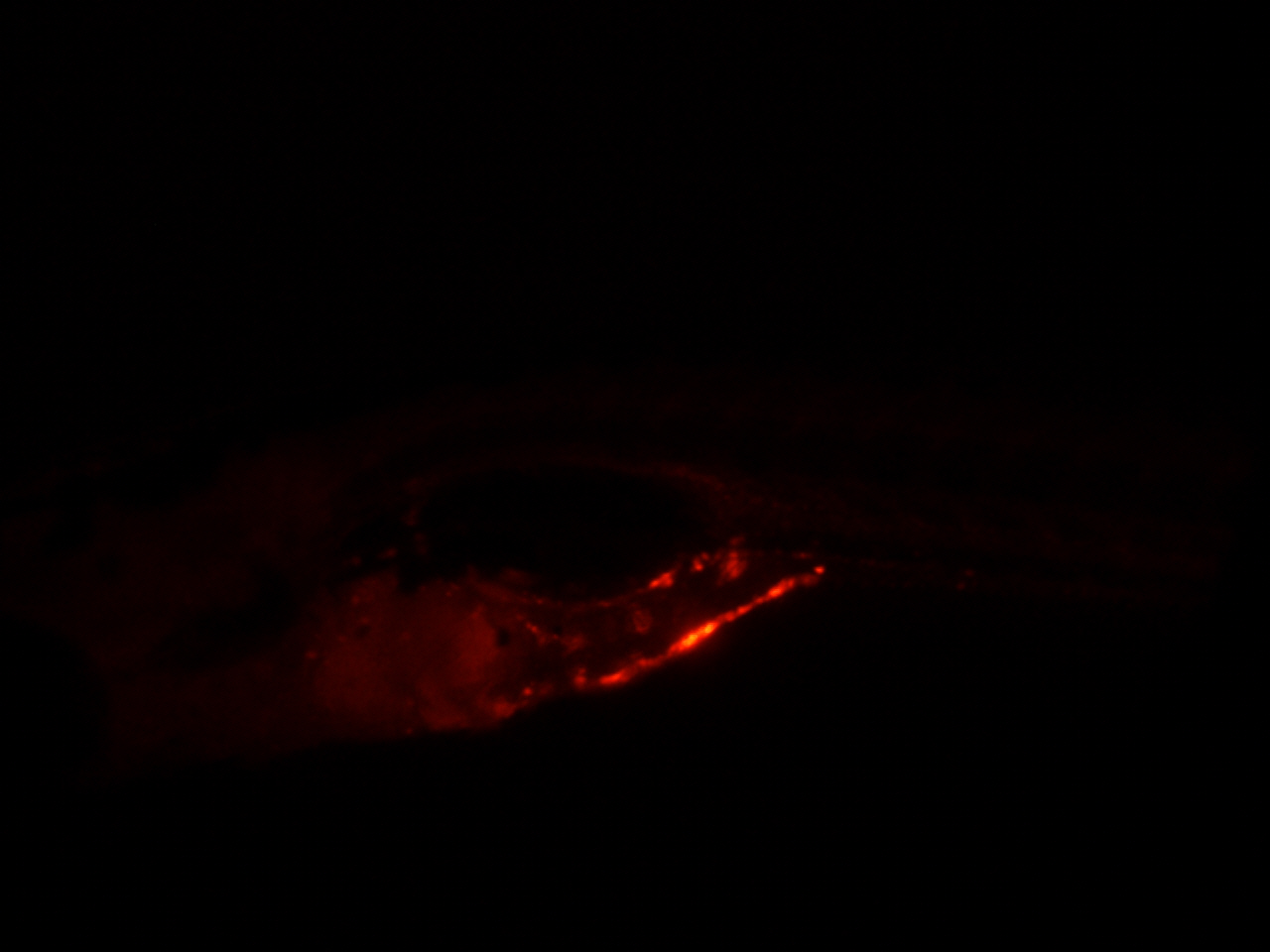

Supplement: Supplementary file 1 [file Data_Sheet_1.ZIP › peptide/6.jpg]

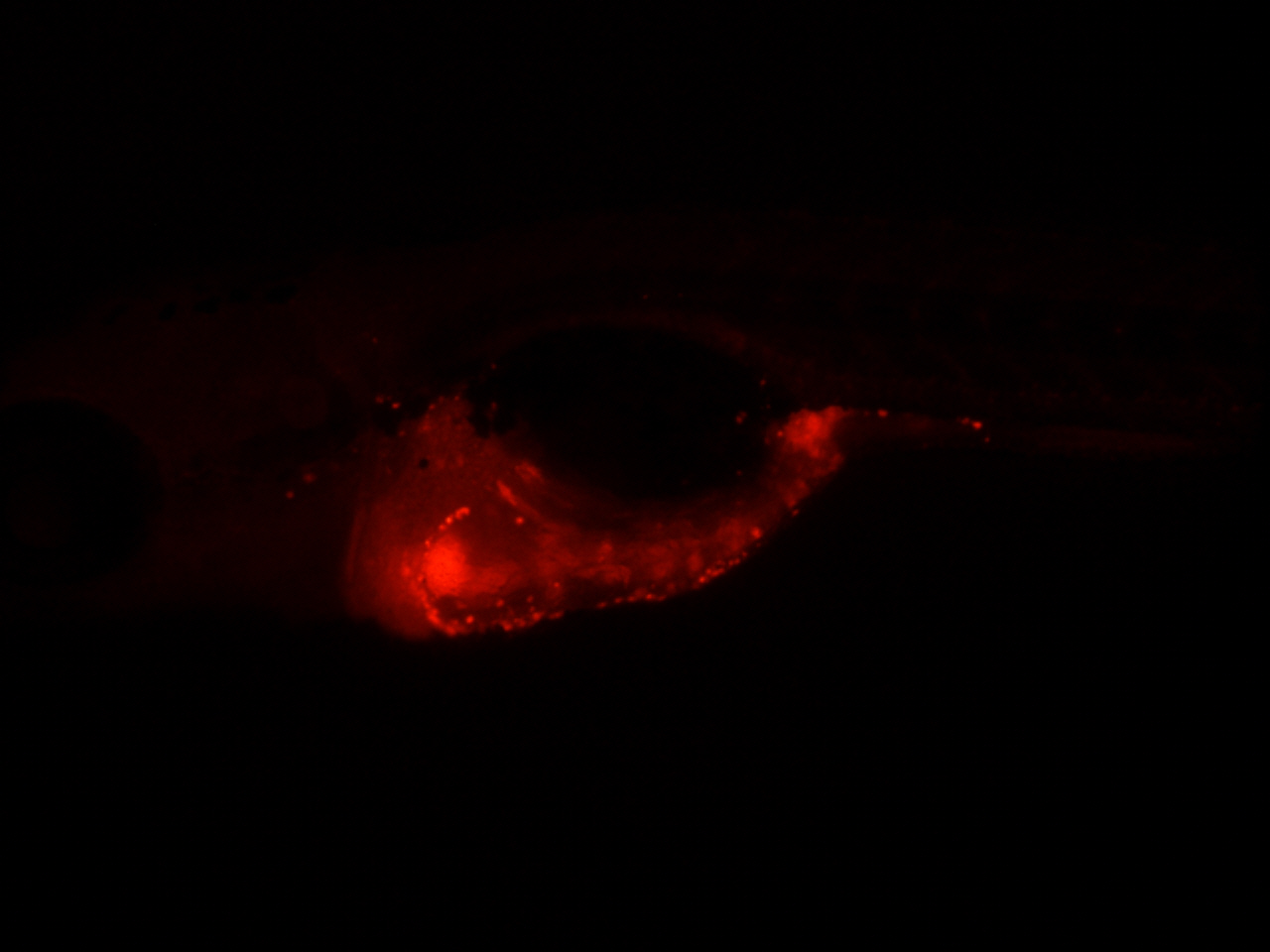

Supplement: Supplementary file 1 [file Data_Sheet_1.ZIP › peptide/7.jpg]

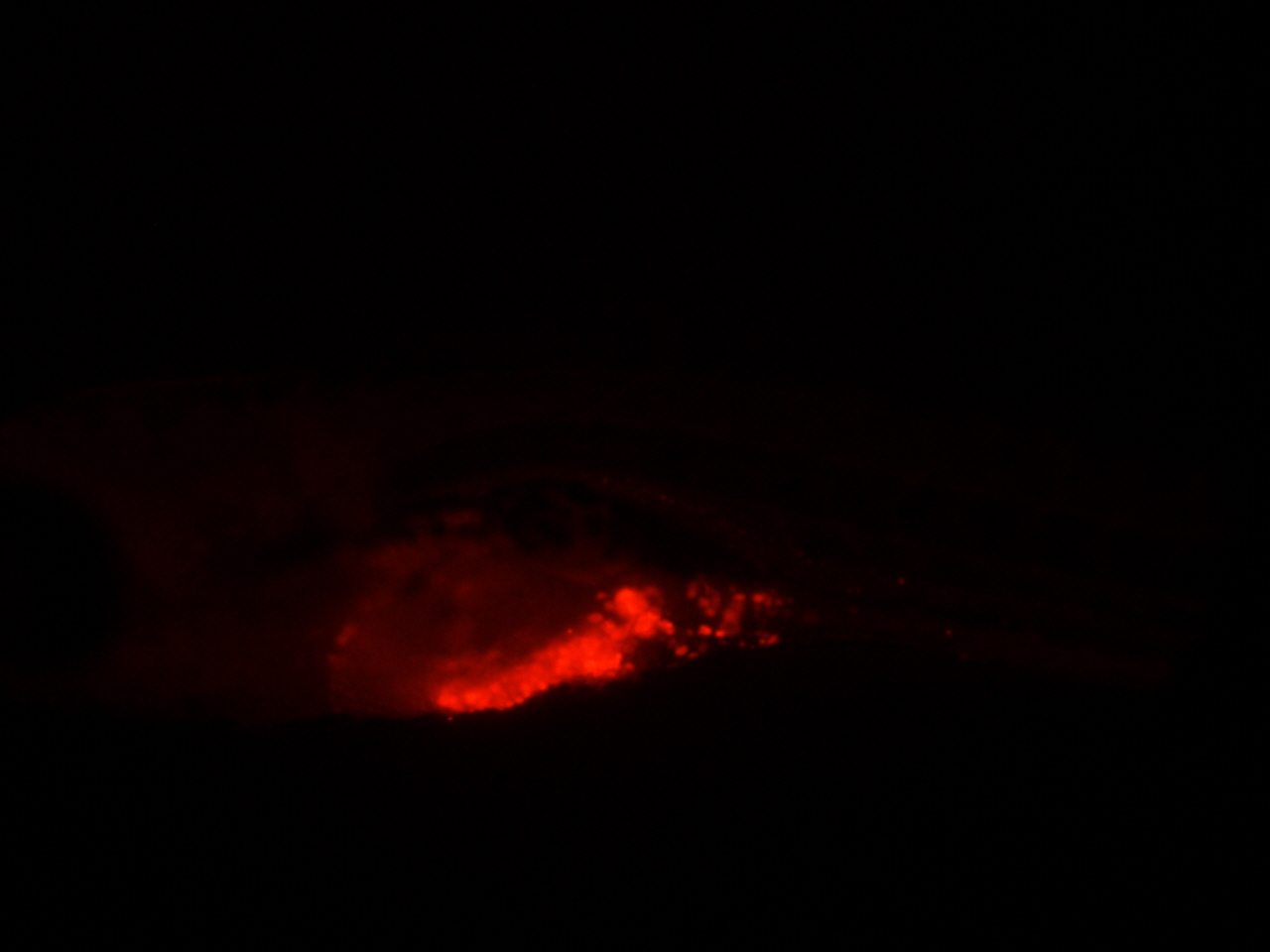

Supplement: Supplementary file 1 [file Data_Sheet_1.ZIP › peptide/8.jpg]

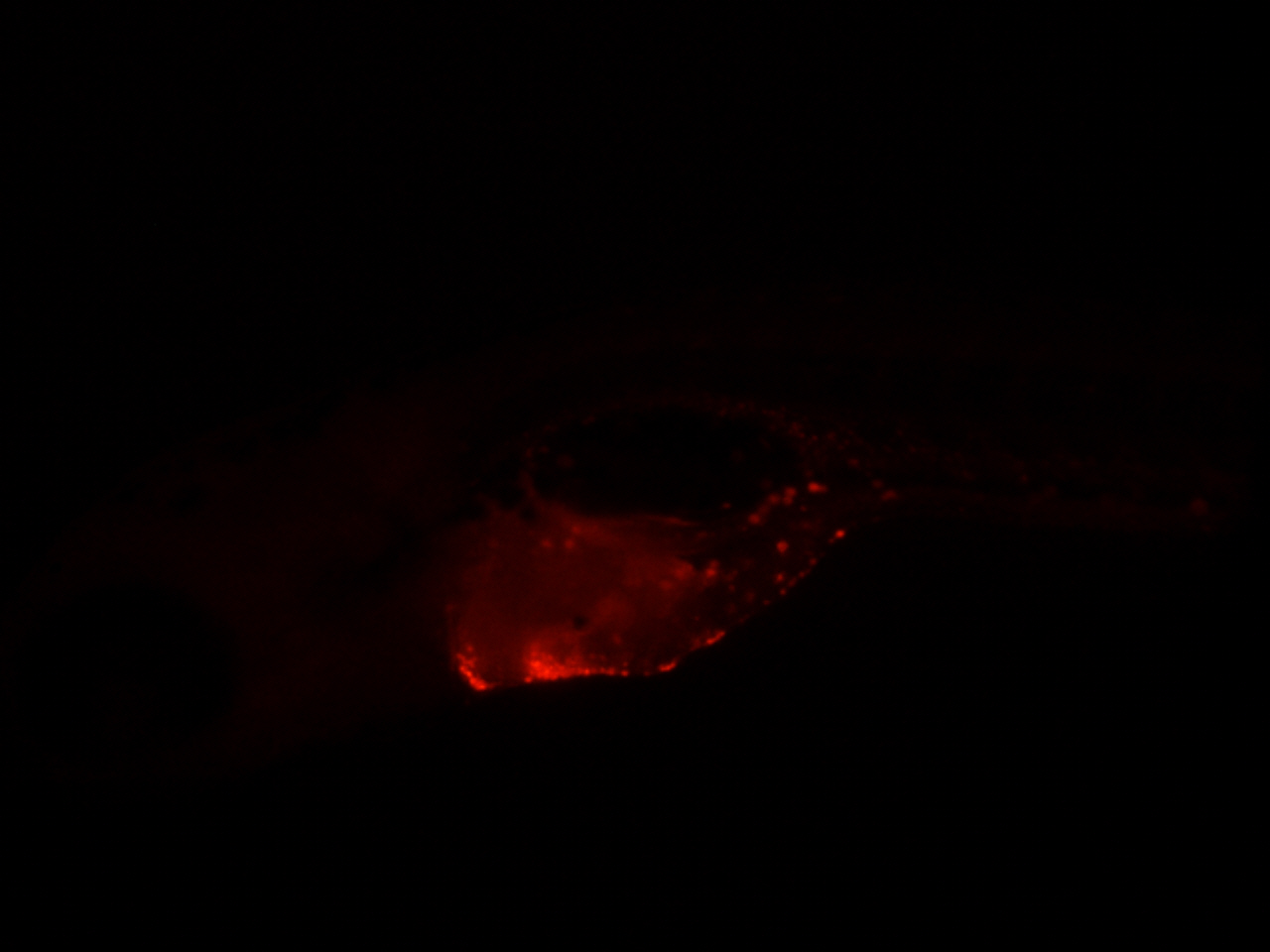

Supplement: Supplementary file 1 [file Data_Sheet_1.ZIP › peptide/9.jpg]

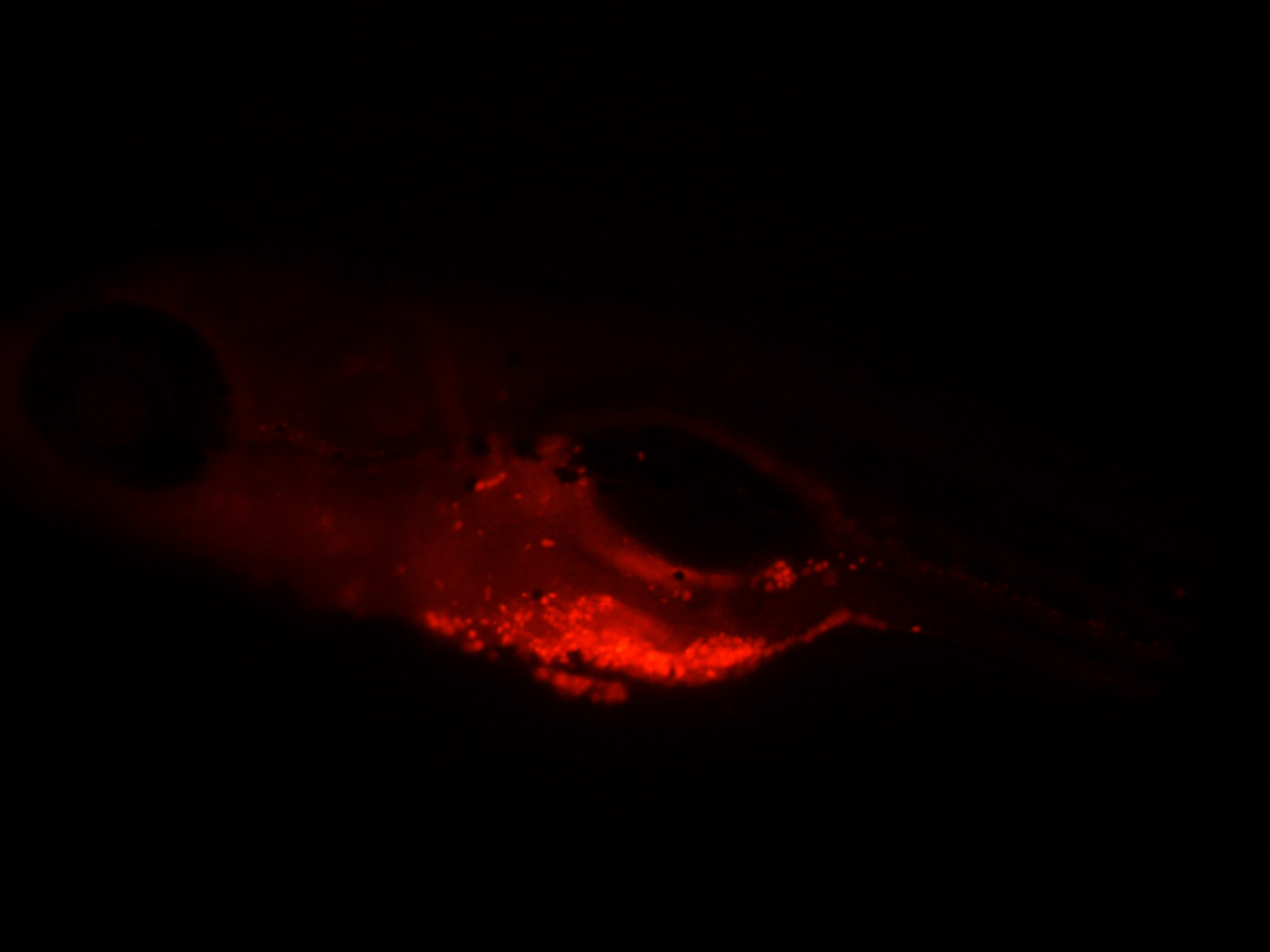

Supplement: Supplementary file 1 [file Data_Sheet_1.ZIP › peptide/拍摄 1.jpg]

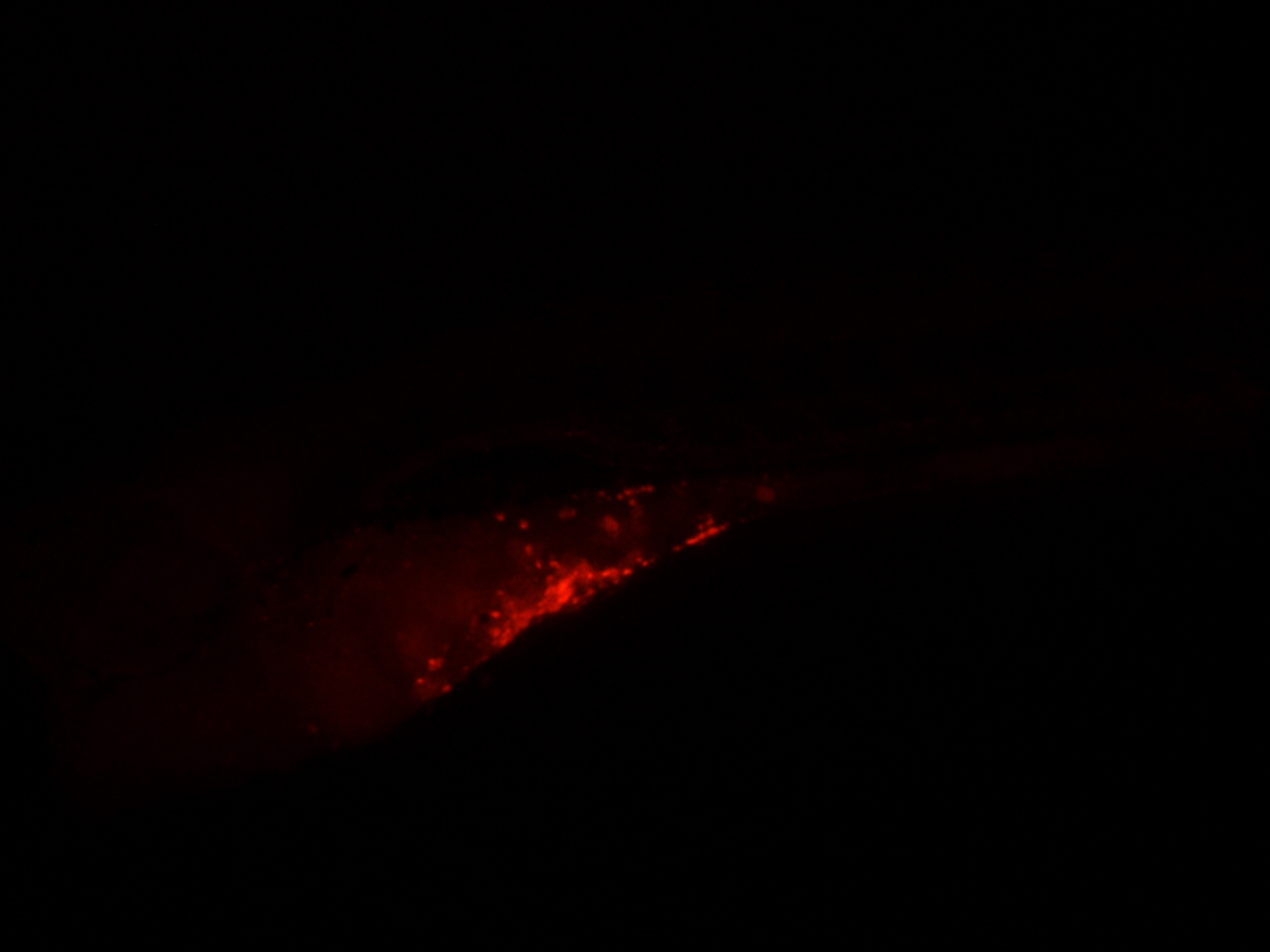

Supplement: Supplementary file 1 [file Data_Sheet_1.ZIP › positive/1.jpg]

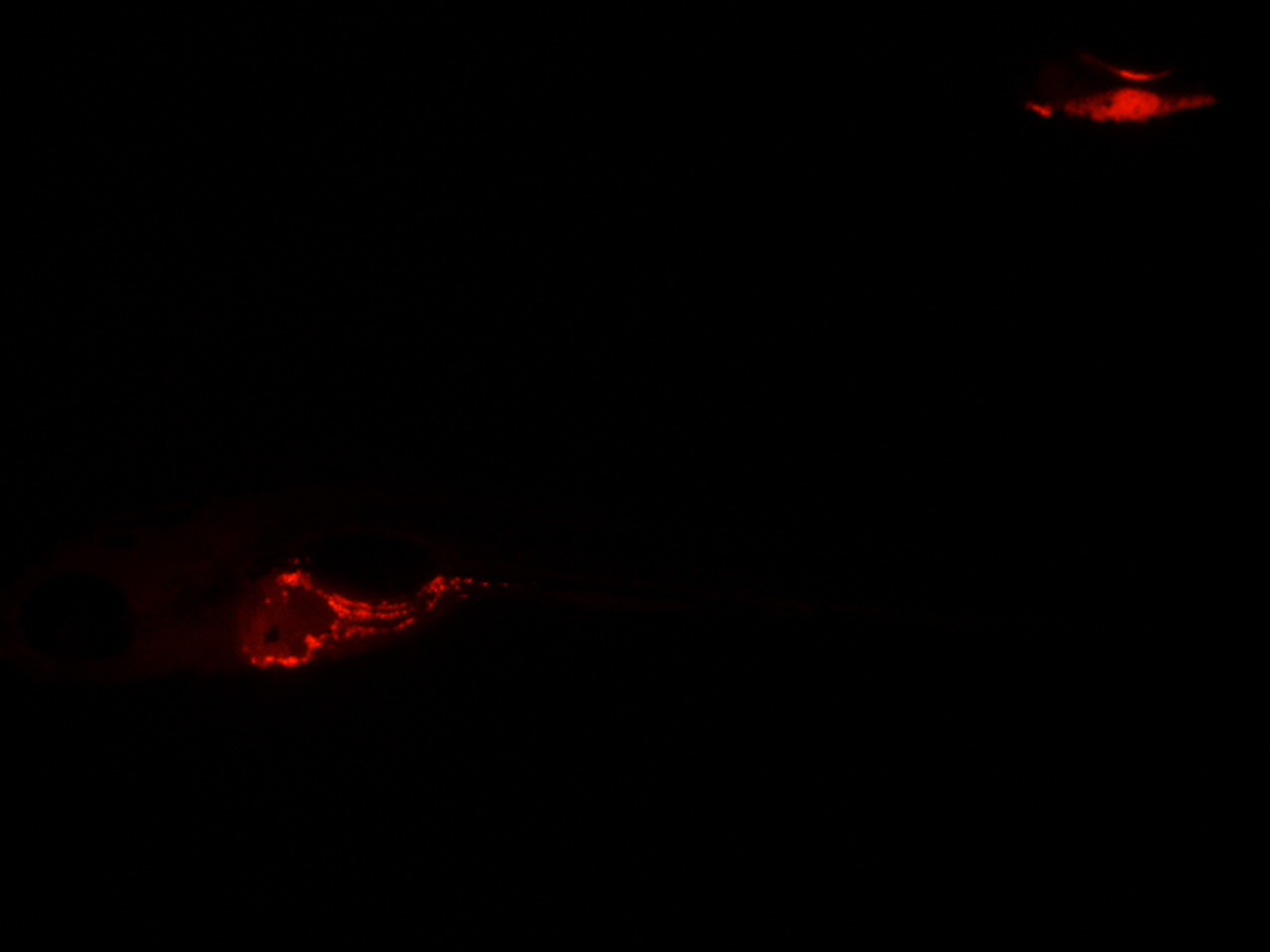

Supplement: Supplementary file 1 [file Data_Sheet_1.ZIP › positive/10-2.jpg]

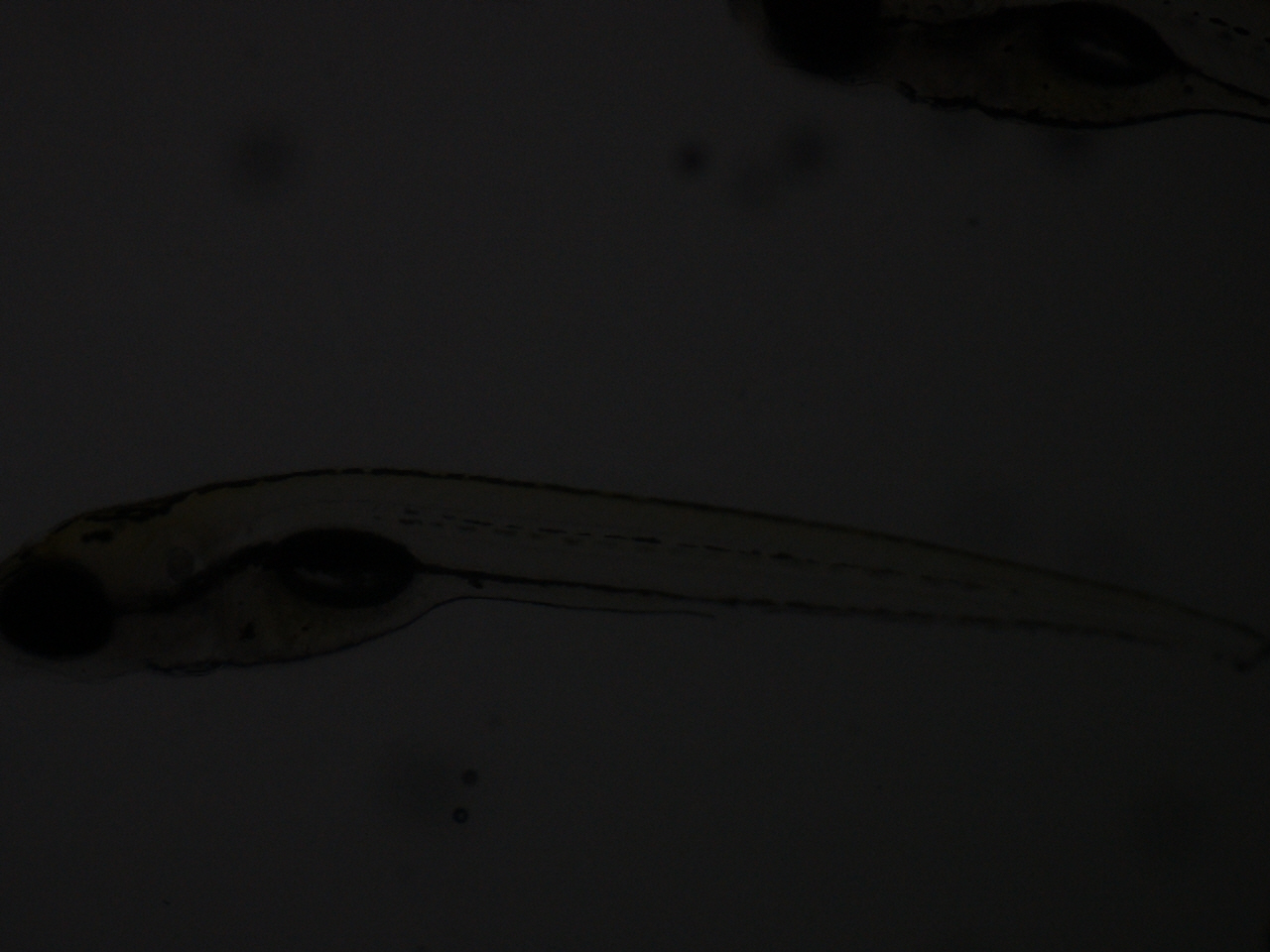

Supplement: Supplementary file 1 [file Data_Sheet_1.ZIP › positive/10-3.jpg]

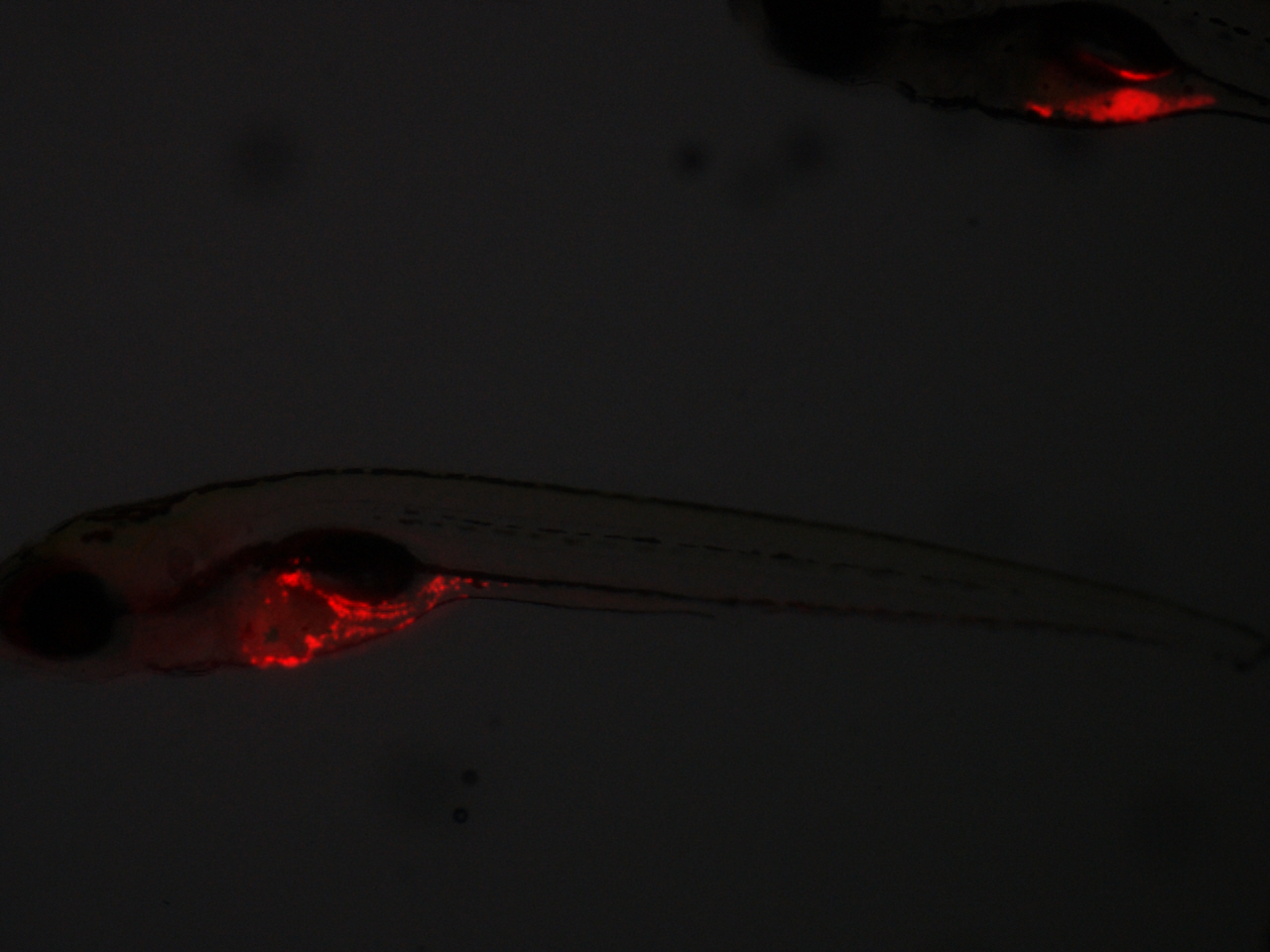

Supplement: Supplementary file 1 [file Data_Sheet_1.ZIP › positive/10-4.jpg]

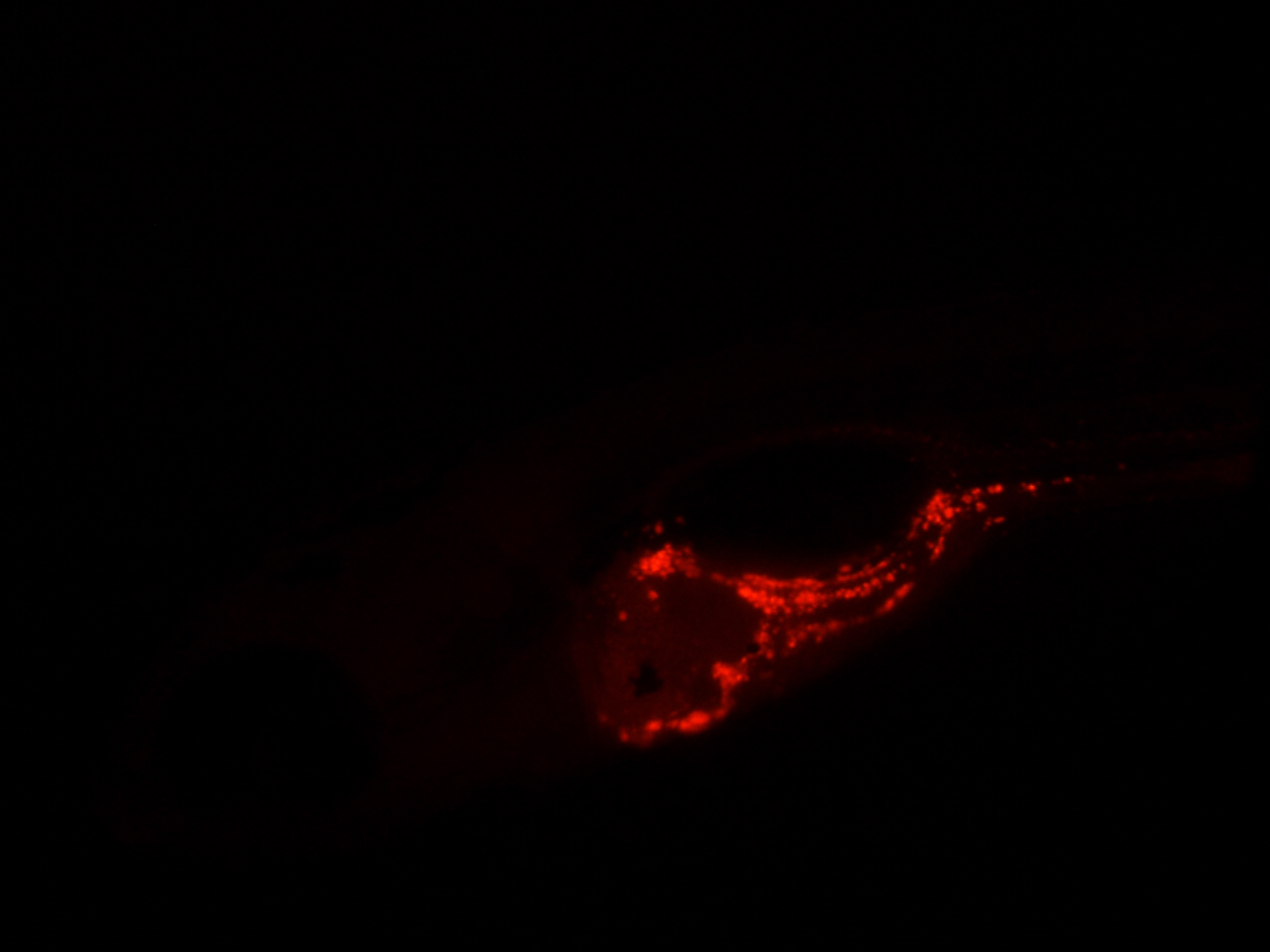

Supplement: Supplementary file 1 [file Data_Sheet_1.ZIP › positive/101.jpg]

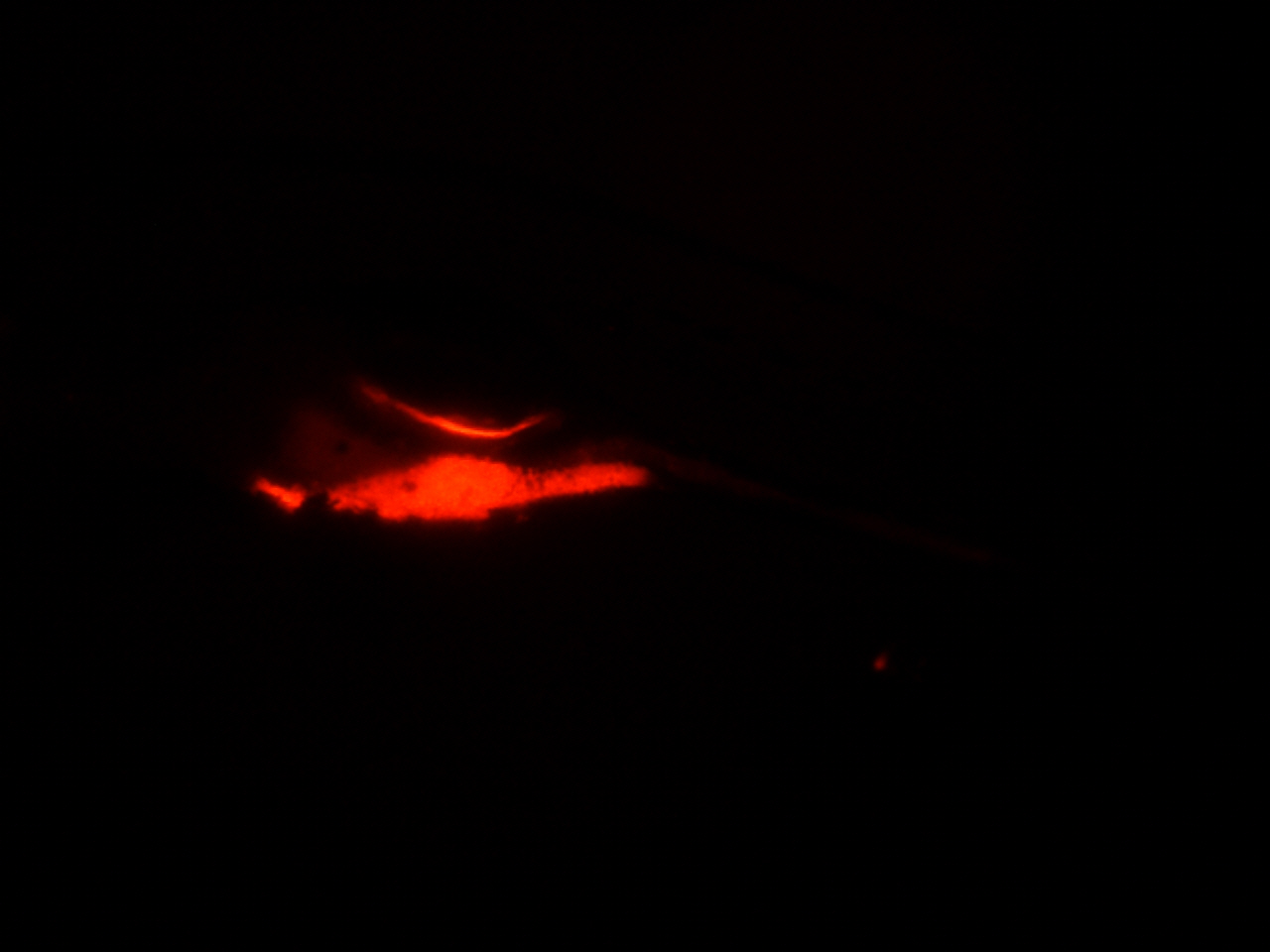

Supplement: Supplementary file 1 [file Data_Sheet_1.ZIP › positive/11.jpg]

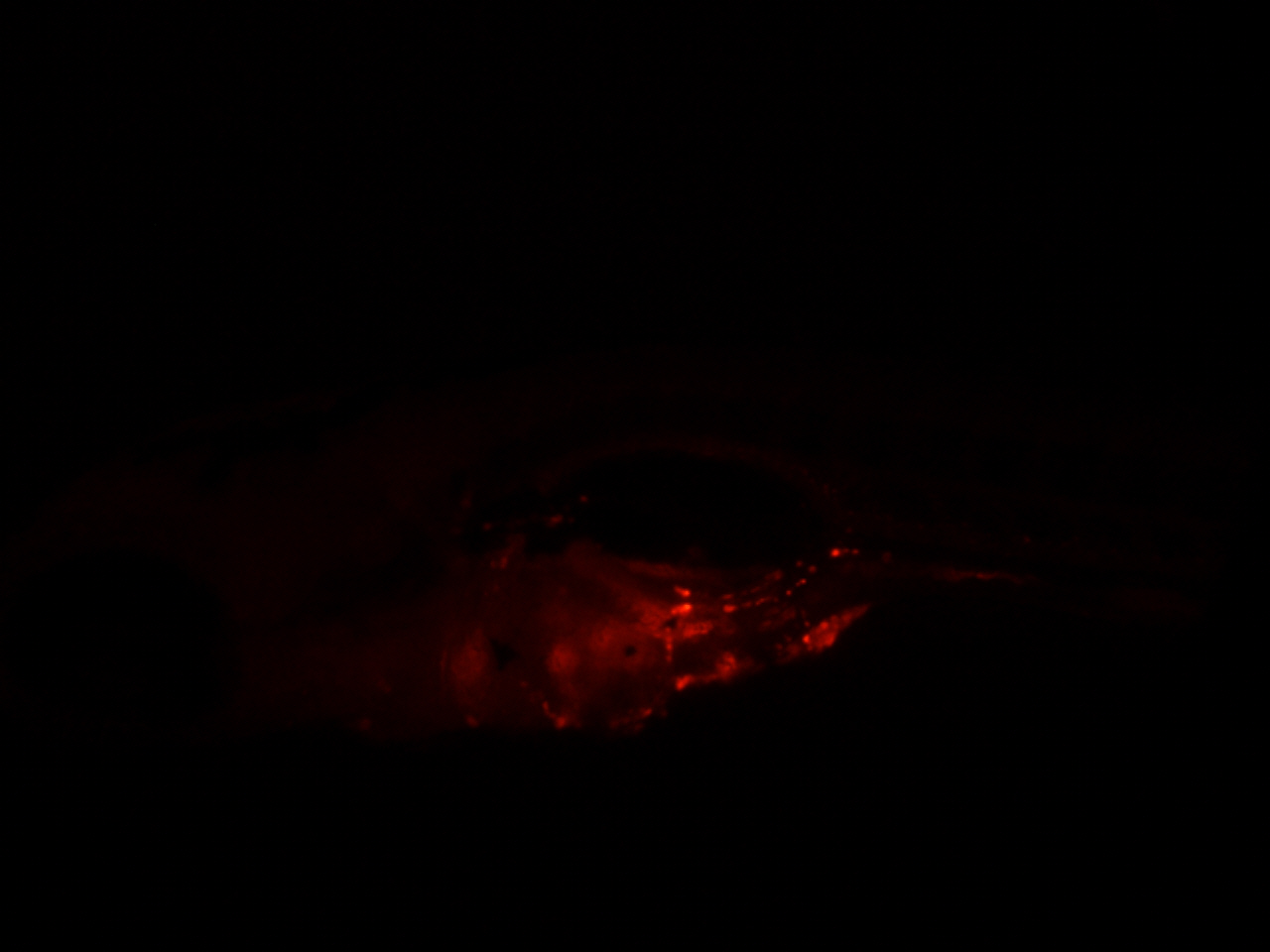

Supplement: Supplementary file 1 [file Data_Sheet_1.ZIP › positive/12.jpg]

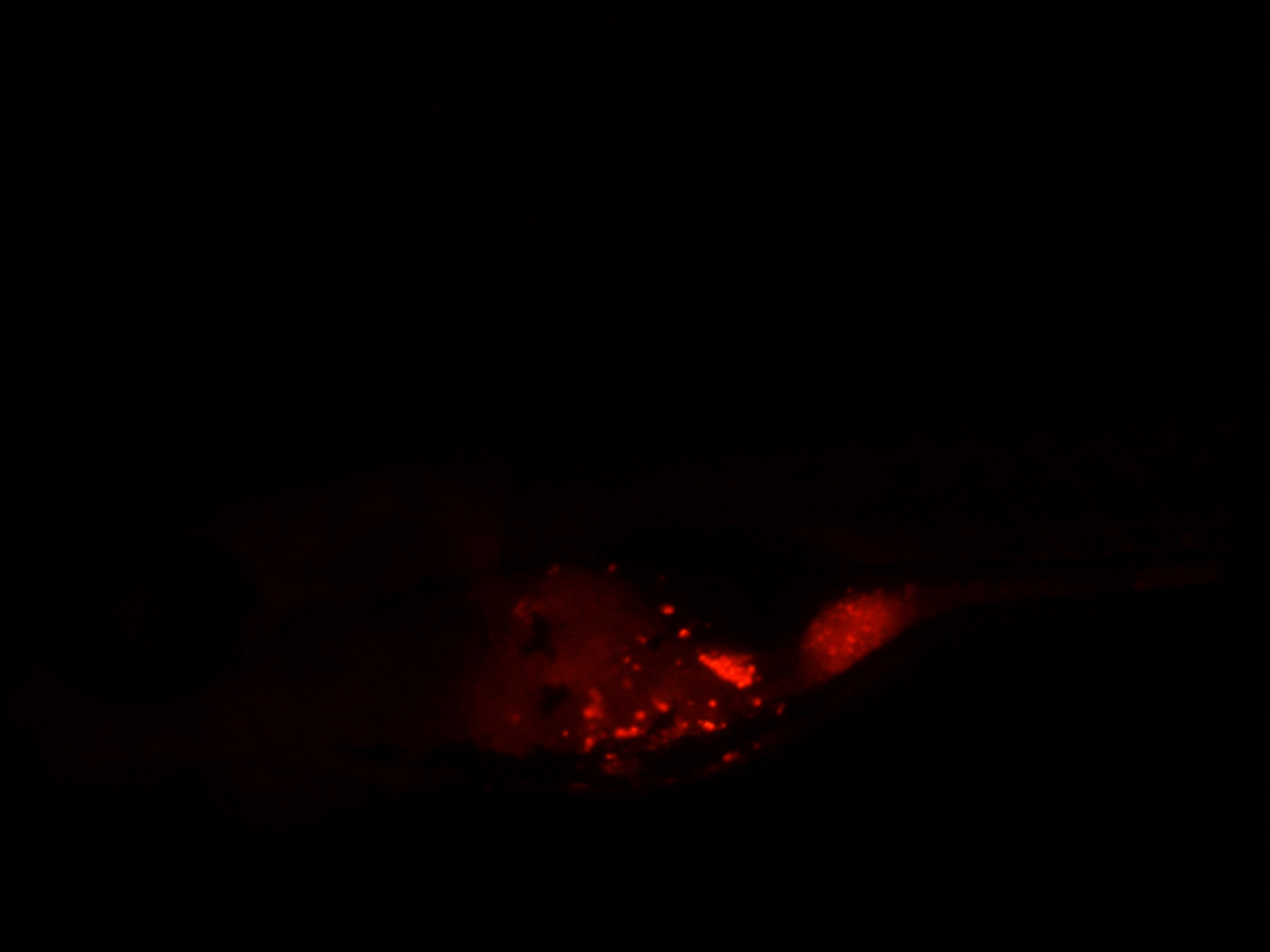

Supplement: Supplementary file 1 [file Data_Sheet_1.ZIP › positive/13.jpg]

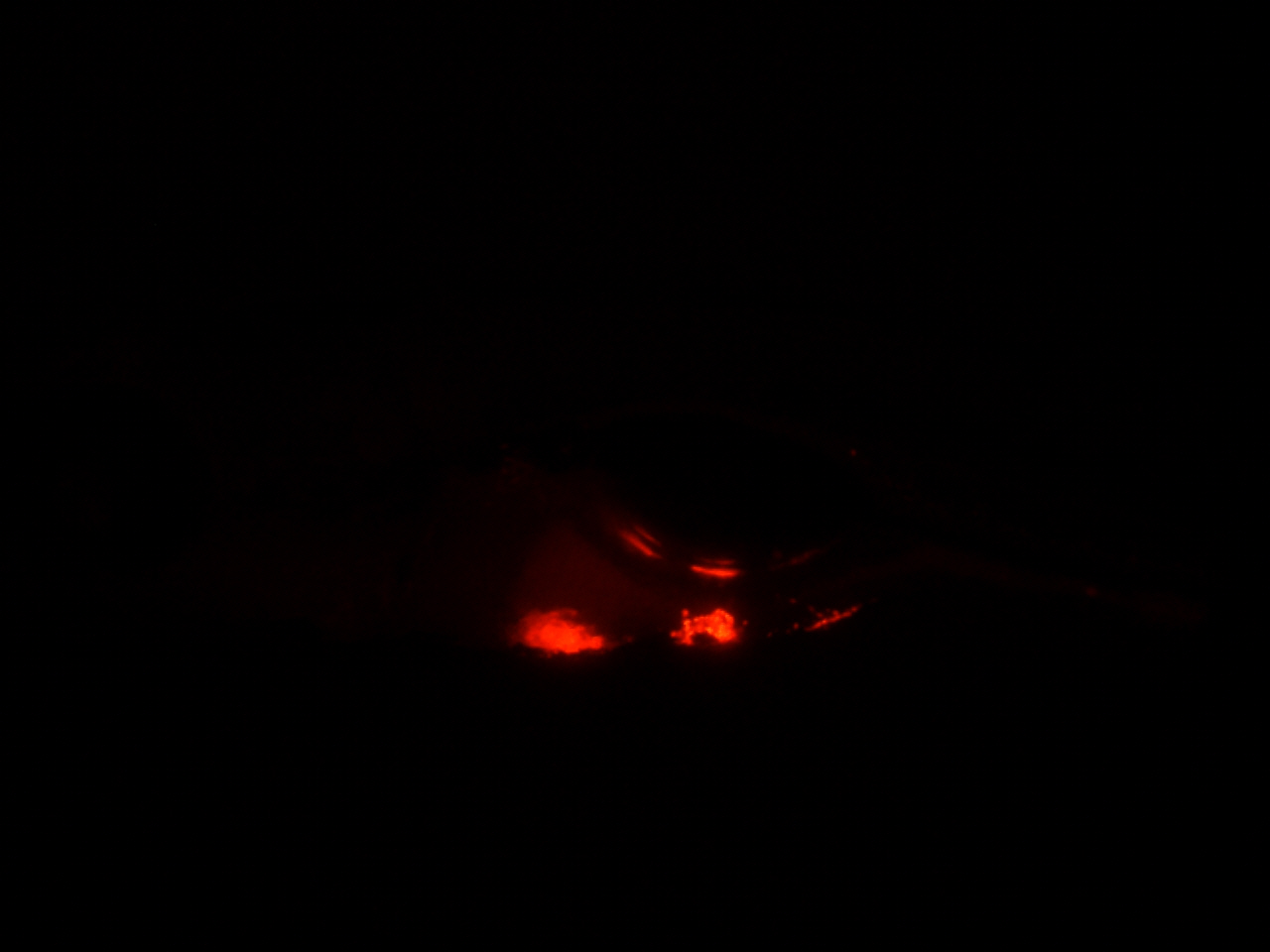

Supplement: Supplementary file 1 [file Data_Sheet_1.ZIP › positive/14.jpg]

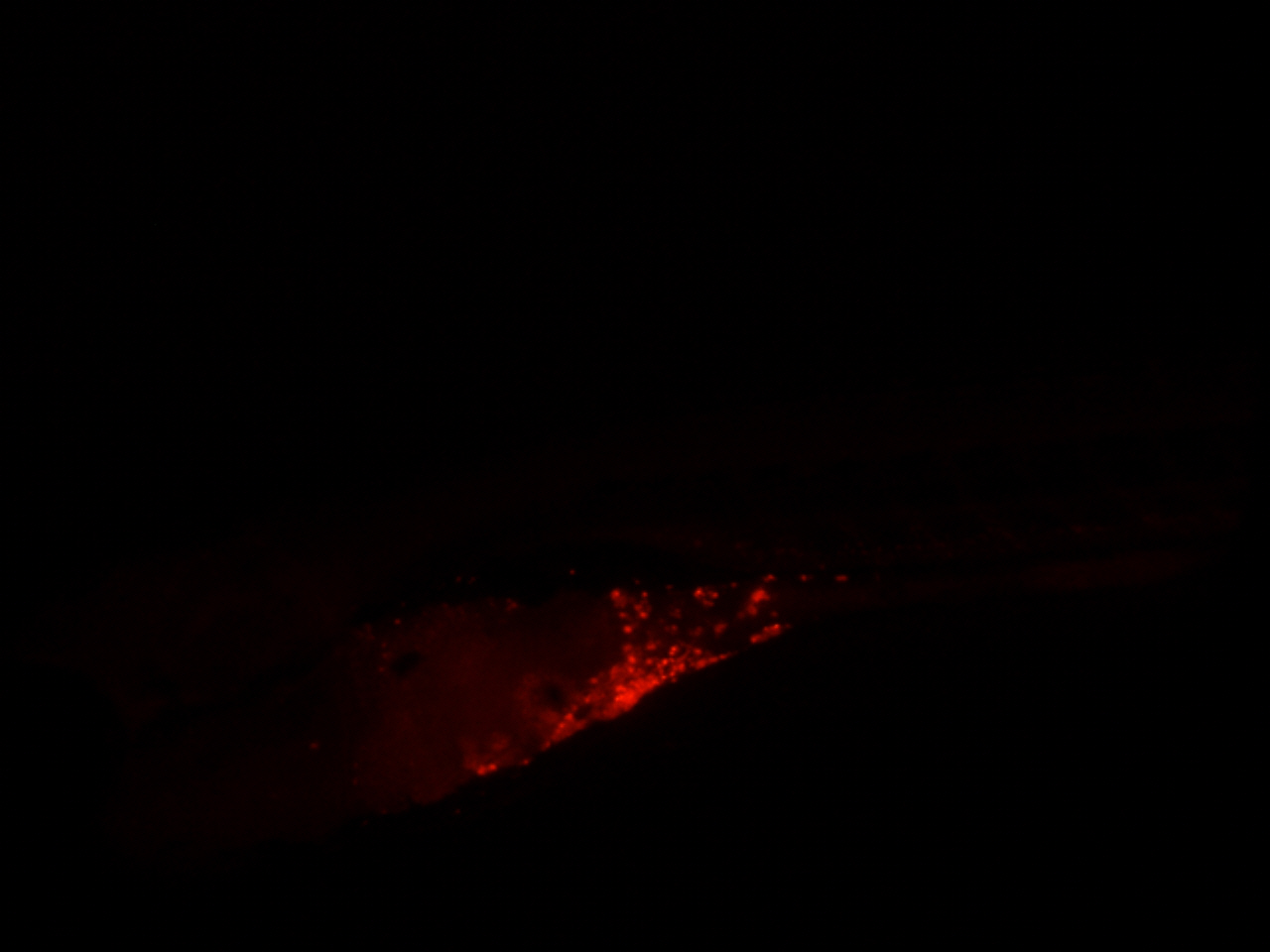

Supplement: Supplementary file 1 [file Data_Sheet_1.ZIP › positive/15.jpg]

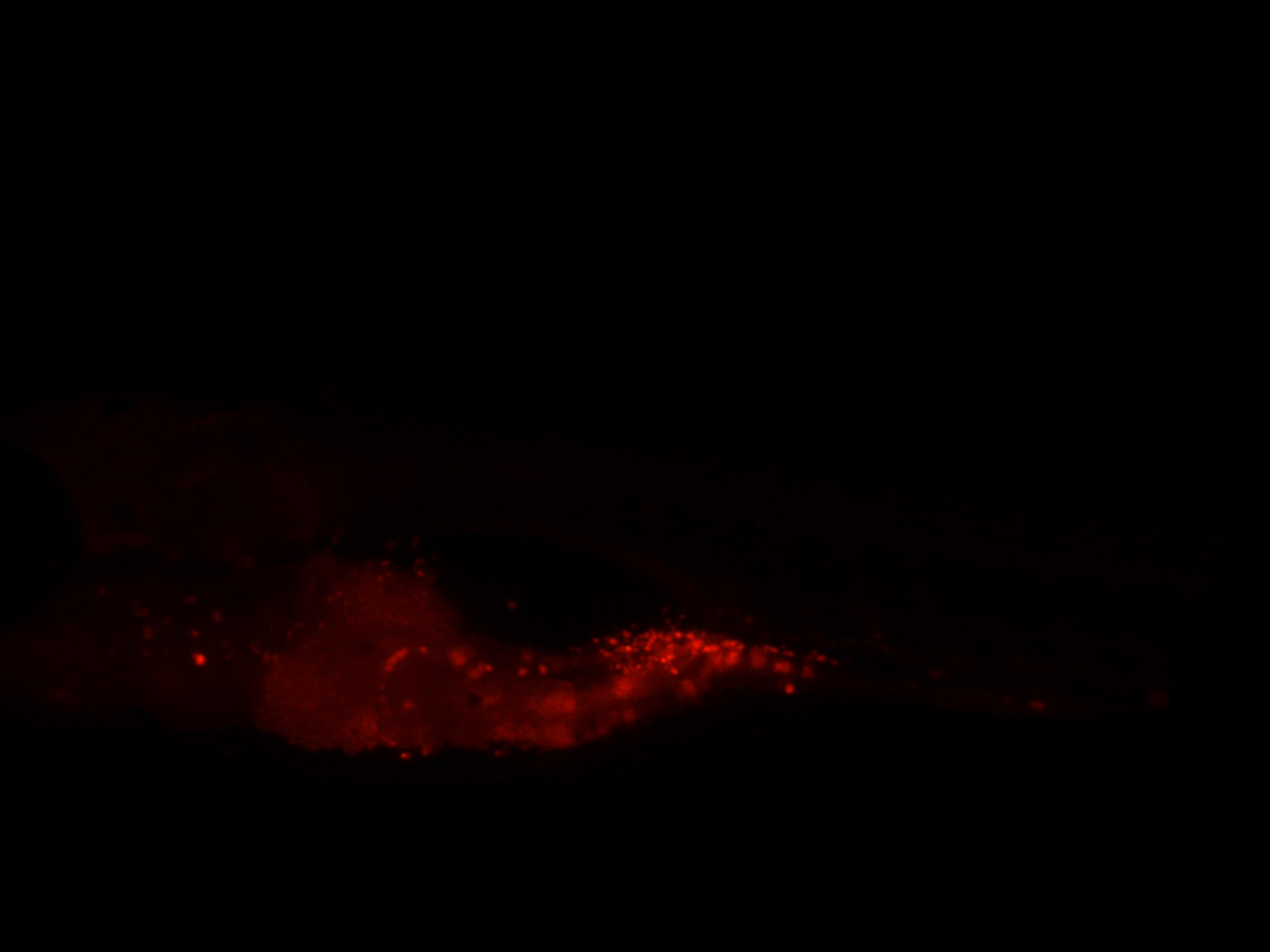

Supplement: Supplementary file 1 [file Data_Sheet_1.ZIP › positive/2.jpg]

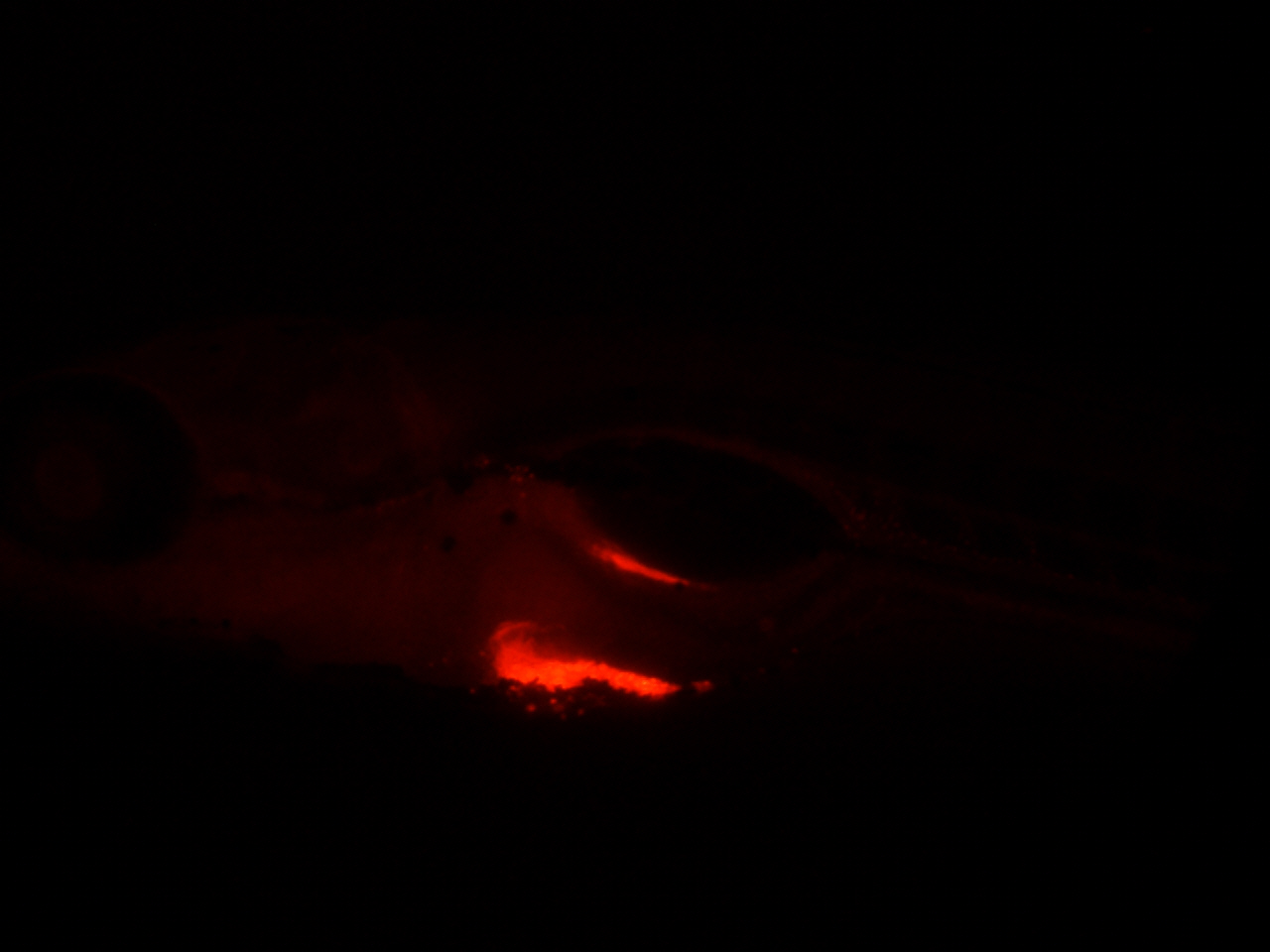

Supplement: Supplementary file 1 [file Data_Sheet_1.ZIP › positive/3.jpg]

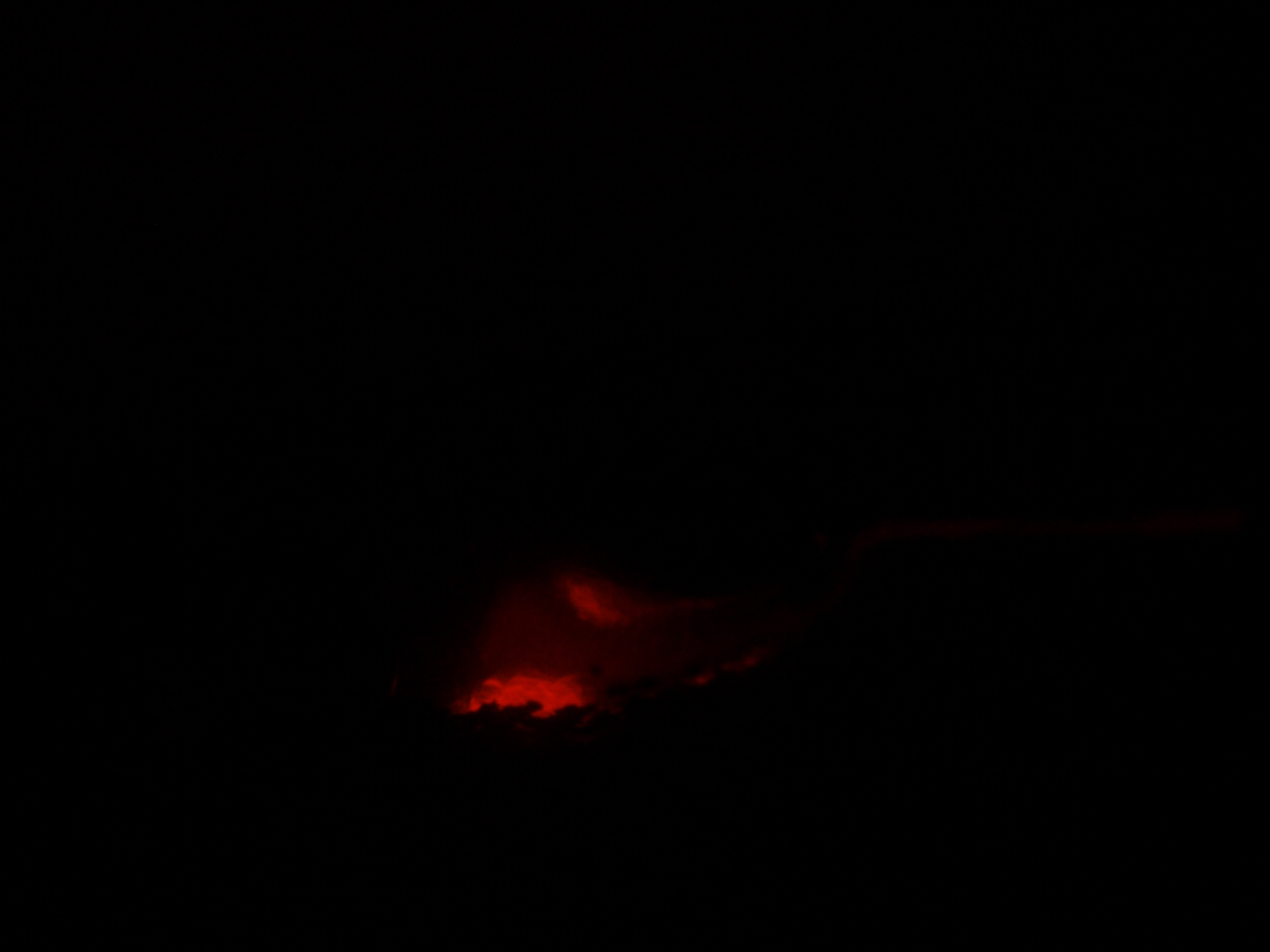

Supplement: Supplementary file 1 [file Data_Sheet_1.ZIP › positive/4.jpg]

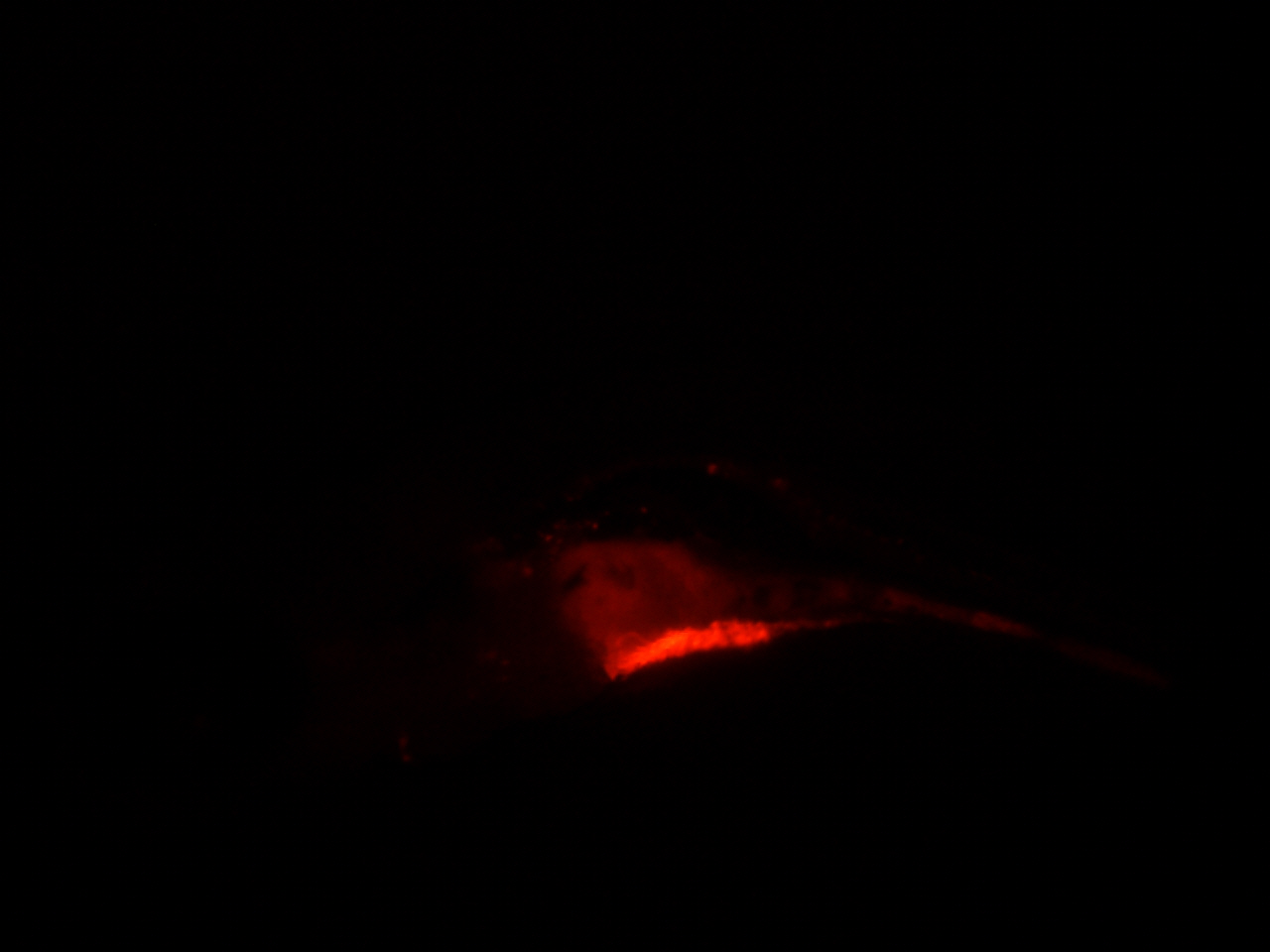

Supplement: Supplementary file 1 [file Data_Sheet_1.ZIP › positive/5.jpg]

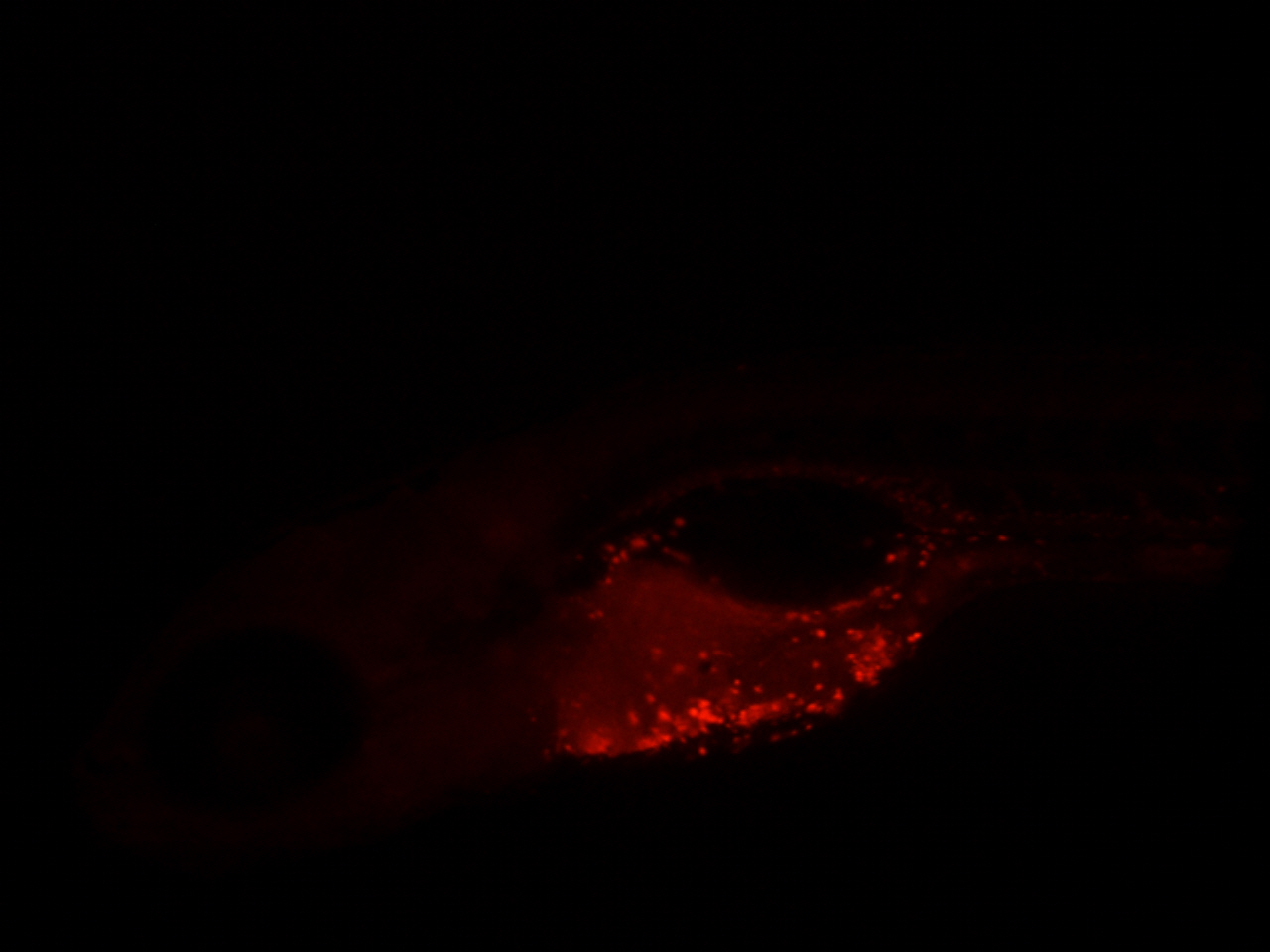

Supplement: Supplementary file 1 [file Data_Sheet_1.ZIP › positive/6.jpg]

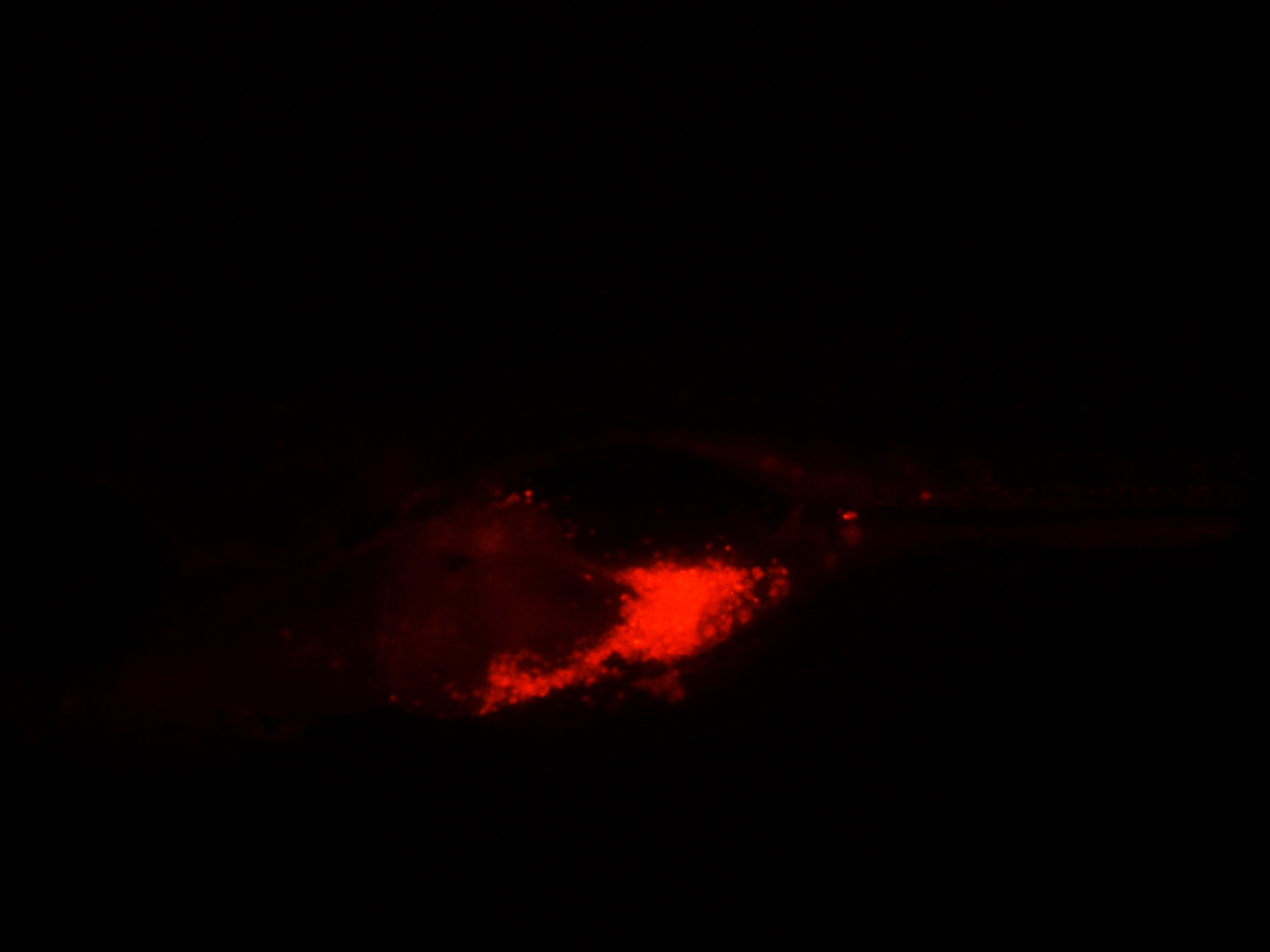

Supplement: Supplementary file 1 [file Data_Sheet_1.ZIP › positive/7.jpg]

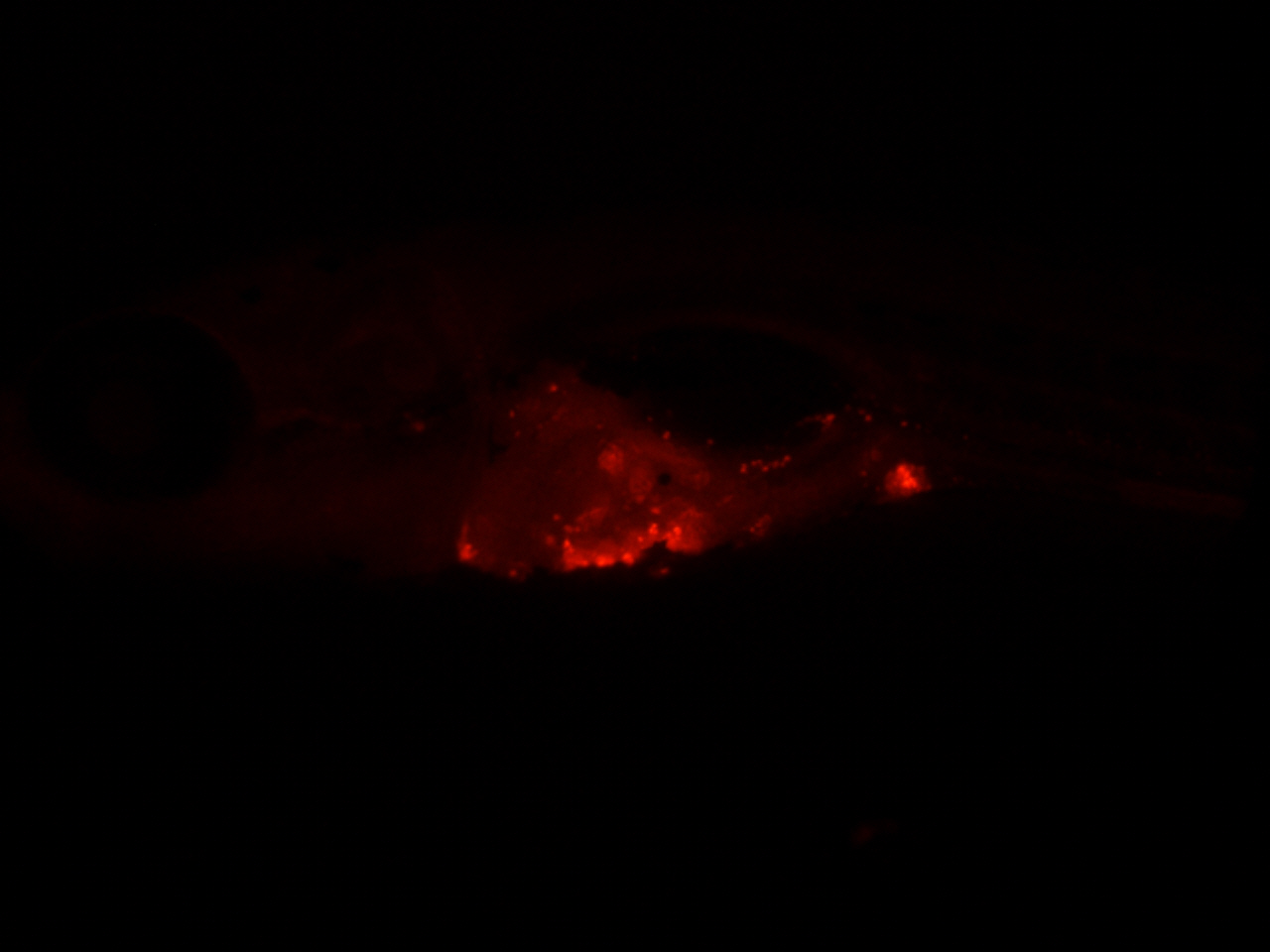

Supplement: Supplementary file 1 [file Data_Sheet_1.ZIP › positive/8.jpg]

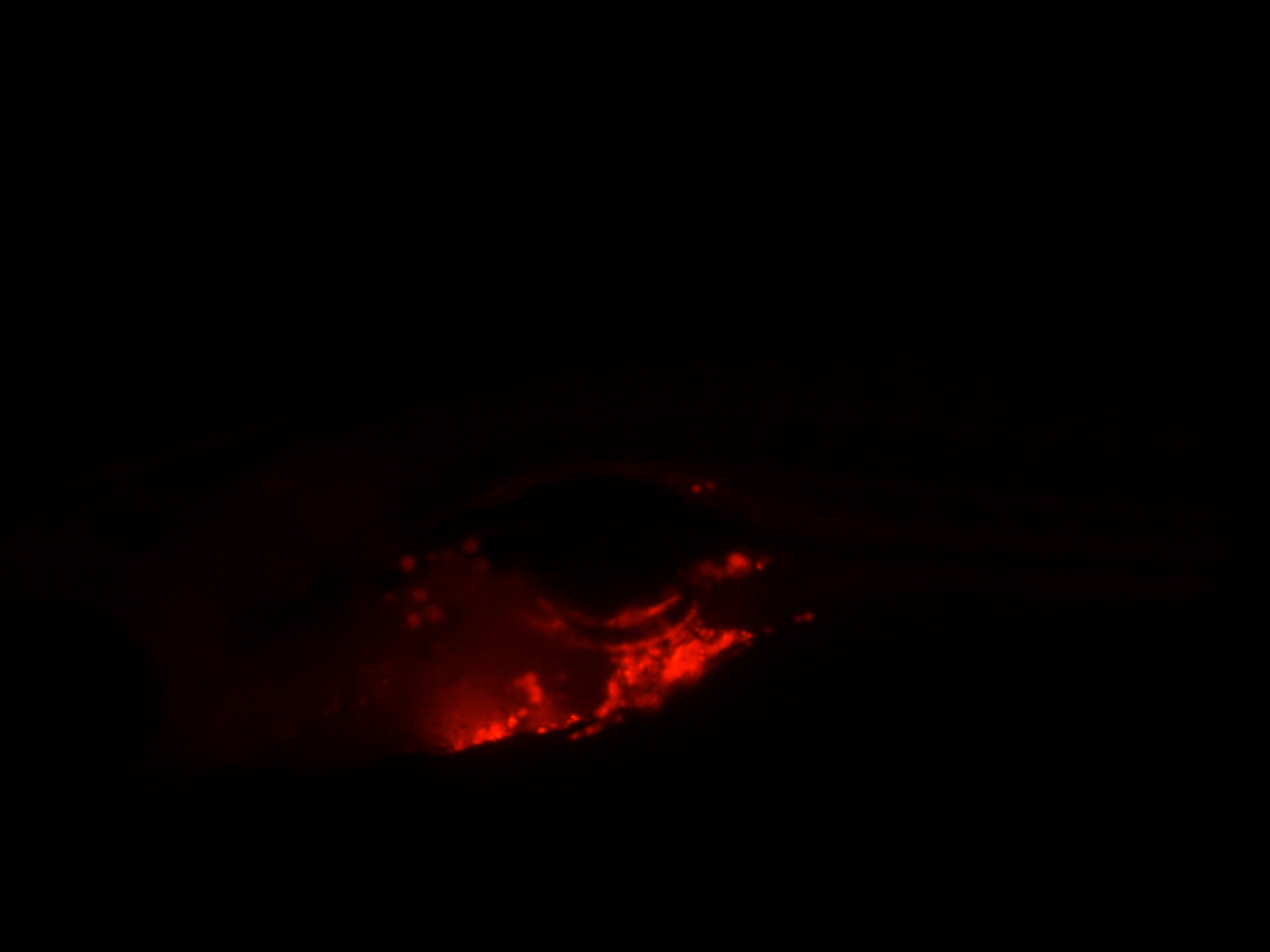

Supplement: Supplementary file 1 [file Data_Sheet_1.ZIP › positive/9.jpg]
